# Local autophagy impairment triggers brain-wide presynaptic remodeling and resilience

**DOI:** 10.1038/s44318-026-00847-4

**Published:** 2026-07-08

**Authors:** David Toppe, Sheng Huang, Janine Lützkendorf, Pin-Lian Jiang, Raquel Suárez-Grimalt, Alexander Neumann, Zhiying Zhao, Péter Lőrincz, Lisa Scheunemann, Gábor Juhász, Fan Liu, Marta Maglione, Stephan J Sigrist

**Affiliations:** 1 Department of Biology, Chemistry, Pharmacy, Institute for Biology/Genetics, Freie Universität Berlin, 14195 Berlin, Germany; 2 NeuroCure Cluster of Excellence, Charité Universitätsmedizin, 10117 Berlin, Germany; 3 Department of Structural Biology, Leibniz Forschungsinstitut für Molekulare Pharmakologie (FMP), 13125 Berlin, Germany; 4 Institute of Genetics, HUN-REN Biological Research Centre Szeged, Szeged, Hungary; 5 Department of Anatomy, Cell and Developmental Biology, Eötvös Loránd University, Budapest, Hungary; 6 Charité Universitätsmedizin Berlin, Charitéplatz 1, 10117 Berlin, Germany; 7 Department of Biology, Chemistry, Pharmacy, Institute for Chemistry and Biochemistry, SupraFAB, 14195 Berlin, Germany

**Keywords:** Autophagy & Cell Death, Neuroscience

## Abstract

Neural circuits must remain functionally stable while adapting to changing demands and levels of stress. While this balance is thought to rely on plasticity programs integrating molecular and activity-dependent signals, mechanistic models of how such adaptations are orchestrated remain limited. Here, we show that impairment of autophagy in the *Drosophila* mushroom body (MB) induces brain-wide, post-transcriptional remodeling of presynaptic active zones, characterized by increased expression levels of active zone scaffold proteins, reduced abundance of calcium channel subunits, and elevated levels of Shaker-type potassium channels. This remodeling promotes organismal resilience, as reflected by increased sleep and extended lifespan. Mechanistically, early-life activation of this program is sufficient to extend lifespan, identifying synaptic remodeling as a causal driver of adaptive responses. MB-specific autophagy disruption further leads to non-cell autonomous accumulation of autophagic substrates across the brain, consistent with a system-level proteostatic imbalance in which degradative pathways remain active, but appear insufficient to match cargo load. Our findings identify autophagy in the mushroom body as a key regulator of brain-wide synaptic architecture and resilience, and establish a genetically tractable model for how local proteostatic impairment can trigger adaptive, system-level circuit remodeling.

## Synopsis

Neuronal networks must maintain functional stability despite fluctuating metabolic demands and diverse cellular stressors, highlighting the importance of circuit-level adaptive resilience mechanisms. This study shows that local autophagy impairment within the *Drosophila* mushroom body (MB) induces system-level proteostatic imbalance, while simultaneously activating a brain-wide presynaptic resilience program (termed PreScale) that promotes sleep and longevity.

- Early-life PreScale activation in response to impaired MB autophagy promotes survival and increases sleep.
- PreScale is characterized by post-transcriptional remodeling of presynaptic active zones, including increased abundance of active zone scaffold proteins, reduced levels of calcium channel subunits, and changes in Shaker-type potassium channels.
- Local impairment of MB autophagy results in non-cell autonomous accumulation of autophagic substrates across the brain, potentially involving partial redistribution of Ref(2)P/P62 protein aggregates from MB neurons to other brain regions.
- Ref(2)P/P62 aggregates detected outside the MB co-localize with ATG8a and are found in degradative lysosomal compartments, indicating active degradative processing rather than complete proteostasis failure in these brain regions.

## Abstract (web summary)

Local autophagy impairment in the mushroom body further causes system-level proteostatic imbalance.

## Graphical abstract

**Figure:**
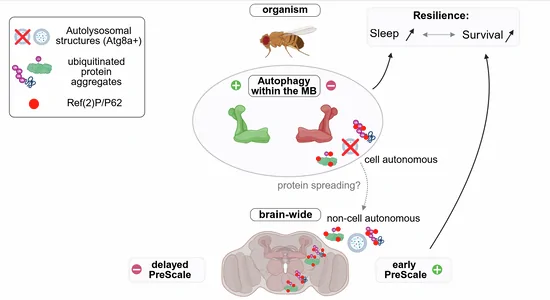

**Figure:**
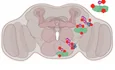

## Introduction

Neuronal networks are continuously challenged by fluctuating metabolic conditions (De Felice and Lourenco, 2015; Maiese, 2023), insufficient sleep (Bringmann, 2019; Havekes and Abel, 2017; Krause et al, 2017), and the gradual loss of proteostasis during aging (Ben-Zvi et al, 2009; Hipp et al, 2019; Mattson and Arumugam, 2018; Rao et al, 2024). While these stressors threaten cellular and circuit stability, the brain has evolved mechanisms to maintain function in the face of adversity. Indeed, many organisms display a remarkable capacity for brain resilience well into advanced mid-life, suggesting that adaptive responses are engaged prior to overt degeneration (Bocancea et al, 2021; Feder et al, 2009; Huang et al, 2022; Mattson and Arumugam, 2018; Taddei and Duff, 2025). Such early acting programs may actively preserve circuit function and behavioral output through regulated synaptic plasticity and organization.

Autophagy, a lysosome-mediated degradation pathway that clears damaged or superfluous cytoplasmic components, is essential to neuronal health and lifespan (Glick et al, 2010; Karpova et al, 2025; Levine and Kroemer, 2019; Maruzs et al, 2019; Tabibzadeh, 2023). Autophagic efficiency declines with age (Aman et al, 2021; Gupta et al, 2013; Lipinski et al, 2010; Ott et al, 2016; Stavoe et al, 2019) and is impaired in numerous neurodegenerative (Guo et al, 2018; Menzies et al, 2017; Nixon, 2013; Nixon and Rubinsztein, 2024) and neurodevelopmental conditions (Deng et al, 2021; Fassio et al, 2020). In neurons, autophagy is not only essential for maintaining local proteostasis, particularly at long-lived presynaptic terminals (Andres-Alonso et al, 2021; Decet and Verstreken, 2021; Karpova et al, 2025), but may also participate in broader plasticity processes (Chang et al, 2024). Yet, how local autophagy impairment might influence global circuit remodeling and resilience remains poorly understood.

Sleep has emerged as a powerful modulator of neuronal plasticity and resilience. Sleep deprivation perturbs cellular homeostasis and impairs cognitive performance (Bringmann, 2019; Cirelli, 2006), whereas restorative sleep promotes synaptic tuning (Cirelli and Tononi, 2022; Huang et al, 2020; Tononi and Cirelli, 2006, 2014) and metabolic waste clearance from the brain (Xie et al, 2013). In *Drosophila*, the mushroom body (MB), a higher brain center for integration of multisensory information and metabolic signals (Suárez-Grimalt et al, 2024), has been identified as a key regulator of sleep homeostasis (Joiner et al, 2006; Pitman et al, 2006), integrating both sleep-promoting and arousal-related inputs (Sitaraman et al, 2015). The MB is thus well positioned to coordinate brain-wide responses to internal stress.

We previously showed that local autophagy impairment within the MB-intrinsic Kenyon cells (KCs) is sufficient to trigger a brain-wide increase of the presynaptic active zone (AZ) protein Bruchpilot (BRP) (Bhukel et al, 2019), a phenotype which also represents a key feature of the brain-wide presynaptic remodeling program termed PreScale (Huang et al, 2022). PreScale exhibits features of a resilience-promoting plasticity state that is induced in response to sleep deprivation (Gilestro et al, 2009; Huang et al, 2020; Weiss et al, 2024) or emerges during early aging (Gupta et al, 2016; Huang et al, 2022), and its activation has been linked to improved stress tolerance and survival (Huang et al, 2022). Protective interventions such as dietary spermidine supplementation (Gupta et al, 2016) or enhanced sleep (Huang et al, 2022) delay or reduce PreScale activation, supporting the idea that it represents a compensatory response to accumulating physiological stress. In the present study, we build on these observations and provide a detailed molecular and functional characterization of PreScale, identifying its structural logic, temporal dynamics, and mechanistic links to resilience under conditions of local autophagy impairment.

Using RNAi-based approaches to impair autophagy specifically within MB KCs, we show that local disruption of protein degradation is sufficient to accelerate the activation of PreScale across the brain during early aging. Despite this localized autophagy impairment, animals exhibit preserved or even enhanced sleep and extended lifespan, indicating that this remodeling response is not merely compensatory but functionally protective. Quantitative proteomic analysis reveals that PreScale is characterized by a coordinated increase in AZ scaffold proteins, including BRP, RIM, Liprin-α, and Unc13A, accompanied by reduced levels of voltage-gated calcium channels (VGCCs) and increased abundance of the voltage-gated potassium channels Shaker and Shaw. These changes are consistent with a shift in presynaptic excitability and neurotransmitter release properties that may stabilize circuit function under conditions of proteostatic stress. Moreover, we find that early-life activation of this remodeling program is sufficient to promote longevity, establishing PreScale as a causal driver of adaptive responses. In parallel, MB-specific autophagy impairment induces a brain-wide accumulation of autophagic substrates, consistent with a system-level proteostatic imbalance that is actively engaged by degradative pathways rather than reflecting a global collapse of proteostasis.

Together, our findings identify PreScale as a resilience-promoting synaptic plasticity program that can be locally initiated by autophagy impairment in the MB and deployed across the brain to preserve circuit function in the face of proteostatic challenge.

## Results

A transient, brain-wide form of presynaptic plasticity, marked by increased abundance of BRP and associated active zone (AZ) components, was previously described and termed PreScale (Huang et al, 2022). PreScale is induced in response to sleep deprivation (Gilestro et al, 2009; Huang et al, 2020; Weiss et al, 2024; Weiss and Donlea, 2021), and its genetic mimicry through increased *brp* gene copy number has been shown to be sufficient to enhance daily sleep (Huang et al, 2020). In parallel, the early phase of *Drosophila* aging (up to ~20–30 days) is also characterized by a PreScale-like AZ-remodeling process, and again, genetic mimicry through increased *brp* gene copy number suggested that this state contributes to organismal resilience and survival (Gupta et al, 2016; Huang et al, 2022). Supporting this view, interventions that promote anti-aging resilience, such as dietary spermidine supplementation or pharmacologically enhanced sleep, were found to delay or reduce the engagement of PreScale during early brain aging (Gupta et al, 2016; Huang et al, 2022). Notably, the difference in PreScale expression and sleep modulation between protected and control animals peaked at around 15 to 20 days of age, highlighting this early time window as a critical phase for resilience programming (Huang et al, 2022).

### MB autophagy bidirectionally regulates brain-wide PreScale during early aging

In terms of brain regions that may be critical for regulating brain-wide PreScale, the *Drosophila* mushroom body (MB) has emerged as a central regulator of sleep homeostasis (Joiner et al, 2006; Pitman et al, 2006), integrating both sleep-promoting and wake-promoting circuits and signals (Sitaraman et al, 2015), and is therefore well positioned to coordinate brain-wide PreScale responses. But what kinds of molecular signals does the MB process to fulfill this integrative role? In this regard, we previously showed that impairment of autophagy specifically within MB-intrinsic Kenyon cells (KCs) triggers a brain-wide increase in BRP levels, a hallmark of PreScale, during early aging (10 days of age) (Bhukel et al, 2019).

**Figure 1:**
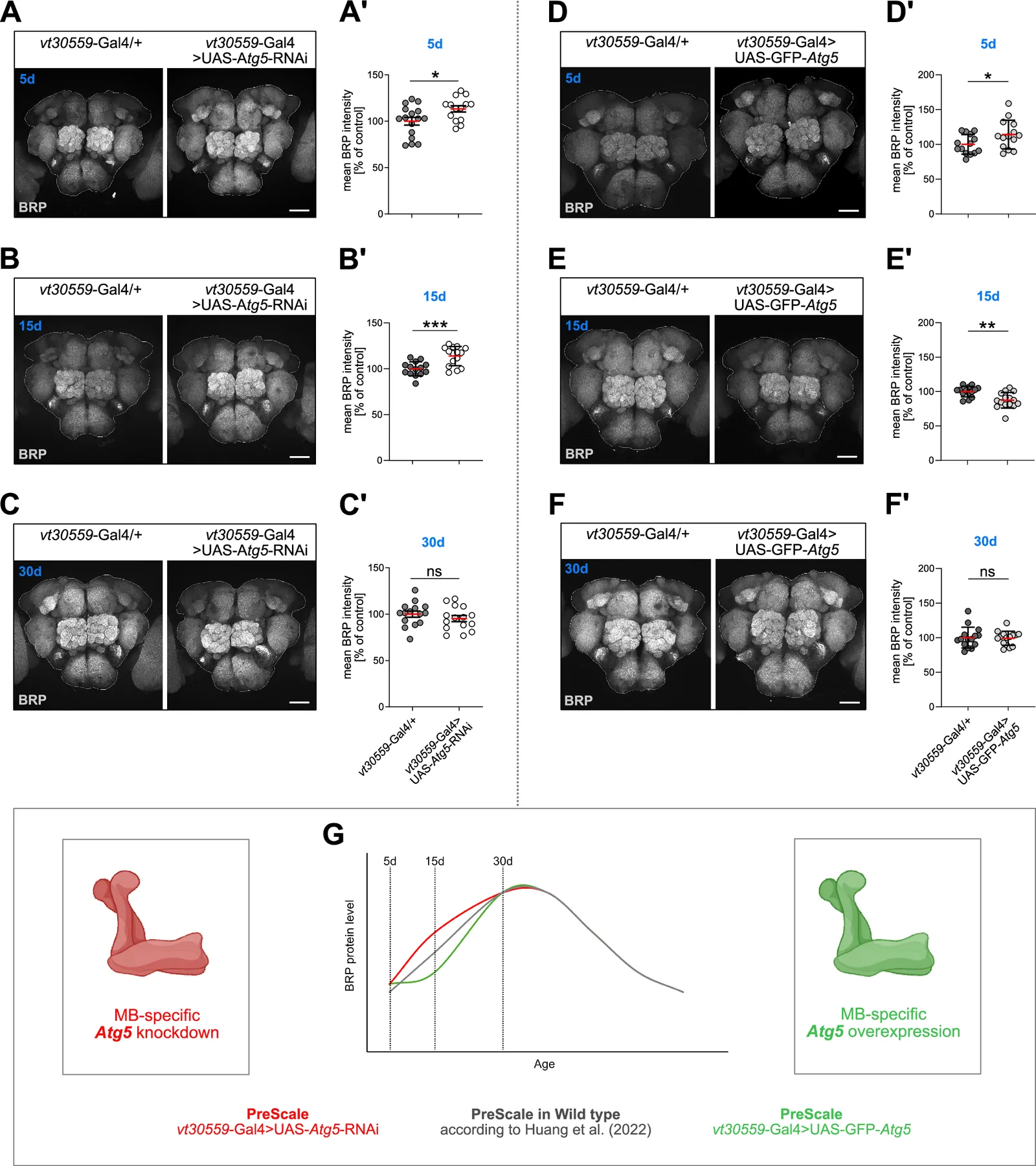
Autophagy within the MB controls brain-wide presynaptic plasticity (PreScale) during early aging. (**A**–**C**) Representative confocal max projections of adult brains of *vt30559*-Gal4>UAS-*Atg5*-RNAi and control *vt30559*-Gal4/+ female flies at 5, 15, or 30 days of age. Brains were immunostained against BRP^Nc82^, and measured whole brain mean BRP intensities for all groups of a specific age were normalized to the respective *vt30559*-Gal4/+ control group. (**A**) Representative confocal max projections and (**A’**) quantification of BRP intensities for 5-day-old female flies. Scale bar = 50 μm; *n* = 14–16 adult brains per genotype pooled from two independent experiments; unpaired two-tailed *t*-test; *p = 0.0227. (**B**) Representative confocal max projections and (**B’**) quantification of BRP intensities for 15-day-old female flies. Scale bar = 50 μm; *n* = 14–15 adult brains per genotype pooled from two independent experiments; unpaired two-tailed *t*-test; ***p = 0.0004. (**C**) Representative confocal max projections and (**C’**) quantification of BRP intensities for 30-day-old female flies. Scale bar = 50 μm; *n* = 15 adult brains per genotype pooled from two independent experiments; unpaired two-tailed *t*-test; ns not significant. (**D**–**F**) Representative confocal max projections of adult brains of *vt30559*-Gal4>UAS-GFP-*Atg5* and control *vt30559*-Gal4/+ female flies at 5, 15, or 30 days of age. Brains were immunostained against BRP^Nc82^, and measured whole brain mean BRP intensities for all groups of a specific age were normalized to the respective *vt30559*-Gal4/+ control group. (**D**) representative confocal max projections and (**D’**) quantification of BRP intensities for 5-day-old female flies. Scale bar = 50 µm; *n* = 14 adult brains per genotype pooled from two independent experiments; unpaired two-tailed *t*-test; *p = 0.0462. (**E**) Representative confocal max projections and (**E’**) quantification of BRP intensities for 15-day old female flies. Scale bar = 50 µm; *n* = 14–15 adult brains per genotype pooled from two independent experiments; unpaired two-tailed *t*-test; **p = 0.0015. (**F**) Representative confocal max projections and (**F’**) quantification of BRP intensities for 30-day-old female flies. Scale bar = 50 µm; *n *= 15 adult brains per genotype pooled from two independent experiments; unpaired two-tailed *t*-test; ns not significant. (**G**) Model of age-dependent increase of brain-wide BRP levels as a surrogate of PreScale activation in wildtype flies and upon genetic manipulation of MB autophagy. Data presented as mean ± SD. Source data are available online for this figure.

Given the progressive and dynamically modulatable build-up of PreScale during early aging in wild-type flies (Gupta et al, 2016; Huang et al, 2022), we systematically investigated the kinetics of PreScale activation in response to MB-specific autophagy manipulation across the early aging period between day 5 and day 30 of age (Fig. 1). In animals with early-onset MB-specific *Atg5* knockdown under control of the strong and KC-specific *vt30559*-Gal4 driver (Plaçais et al, 2017), BRP levels were significantly elevated compared to wildtype *vt30559*-Gal4/+ controls both at 5 (Fig. 1A,A’) and 15 days (Fig. 1B,B’) after eclosion. Notably, by 30 days of age—marking the end of the early aging period—BRP levels in MB-autophagy-impaired animals and control flies were indistinguishable (Fig. 1C,C’). This early-onset BRP upregulation suggests that PreScale, which typically builds up gradually during aging (Huang et al, 2022), exhibits accelerated activation when autophagy is impaired within the MB, before reaching a normal age-matched end-state by 30 days. Importantly, early activation of PreScale seemed to be specific to impaired autophagy within the MB. Knockdown of *Atg5* in sleep-regulating neurons of the ellipsoid body (*R58H05*-Gal4>UAS-*Atg5*-RNAi; Appendix Fig. S1A,B), of the dorsal fan-shaped body (*R23E10*-Gal4>UAS-*Atg5*-RNAi; Appendix Fig. S1C,D), or of helicon cells (*R24B11*-Gal4>UAS-*Atg5*-RNAi; Appendix Fig. S1E,F) did not result in similar brain-wide BRP increases at 15 days of age, but rather decreased BRP levels in the case of *R58H05*-Gal4 (Appendix Fig. S1B) and *R24B11*-Gal4 (Appendix Fig. S1F).

Consistent with the idea that MB autophagy regulates PreScale activation, genetic overexpression of *Atg5* specifically within the MB KCs (*vt30559*-Gal4>UAS-*Atg5*-GFP; Fig. 1D–F’) led to a specific, significant reduction in brain-wide BRP levels once again at 15 days (Fig. 1E,E’), but not at 5 days (Fig. 1D,D’) or at 30 days of age (Fig. 1F,F’). These findings support a model in which the functional status of MB autophagy bidirectionally modulates the timing of activation of brain-wide PreScale-related AZ-remodeling during early *Drosophila* aging (Fig. 1G), highlighting its instructive role in resilience-linked synaptic plasticity.

### MB autophagy bidirectionally controls resilience-associated sleep patterns during early aging

**Figure 2:**
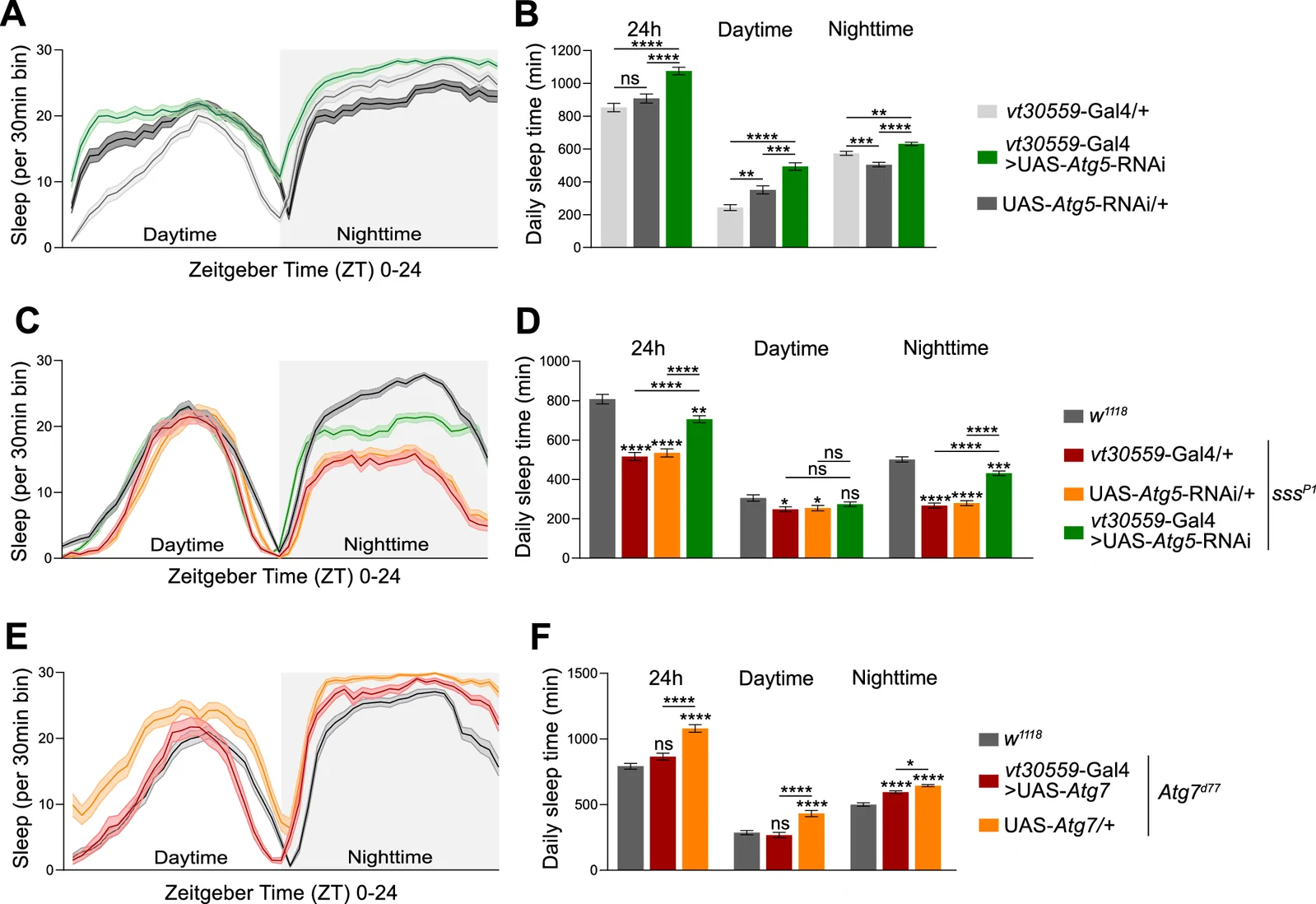
The functional status of MB-intrinsic autophagy bidirectionally controls sleep homeostasis. (**A**,** B**) Sleep parameters of 15-day-old female flies with MB-specific autophagy impairment (*vt30559*-Gal4>UAS-*Atg5*-RNAi) compared to two age- and sex-matched controls carrying only one of the two components of the binary Gal4/UAS system (*vt30559*-Gal4/+ and UAS-*Atg5*-RNAi/+). Sleep data was acquired and averaged over 3 consecutive days. (**A**) Average sleep profiles over 24 h (Zeitgeber Time 0-24) plotted in 30 min bins, including 12 h light (ZT0-ZT12, Daytime) and 12 h darkness (ZT12-ZT24, Nighttime). (**B**) Averaged daily sleep amounts in mins over 24 h, during Daytime and Nighttime. *n* = 45–46 flies per genotype pooled from two independent experiments. 24 h ****p < 0.0001; Daytime **p = 0.0034, ***p = 0.0002, ****p < 0.0001; Nighttime **p = 0.0035, ***p = 0.0003, ****p < 0.0001; ns not significant. (**C**,** D**) Sleep parameters of 5-day-old female *sss*^*P1*^ mutants with MB-restricted knockdown of *Atg5* gene expression (*sss*^*P1*^*;vt30559*-Gal4>UAS-*Atg5*-RNAi) were compared to age- and sex-matched controls in the same *sss*^*P1*^ genetic background (*sss*^*P1*^;UAS-*Atg5*-RNAi/+ and *sss*^*P1*^*;vt30559*-Gal4/+) as well as to a *w*^*1118*^ wild-type control. Sleep data were acquired and averaged over 3 consecutive days. (**C**) Average sleep profiles over 24 h (Zeitgeber time 0–24) plotted in 30 min bins, including 12 h light (ZT0-ZT12, Daytime) and 12 h darkness (ZT12-ZT24, nighttime). (**D**) Averaged daily sleep amounts in mins over 24 h, during daytime and nighttime. *n* = 46–58 flies per genotype pooled from two independent experiments. 24 h **p = 0.0028, ****p < 0.0001; daytime *w*^*1118*^ vs. *sss*^*P1*^*;vt30559-*Gal4/+ *p = 0.0227, *w*^*1118*^ vs. *sss*^*P1*^*;*UAS-*Atg5*-RNAi/+ *p = 0.0336; nighttime ***p = 0.0007, ****p < 0.0001; ns not significant. (**E**,** F**) Sleep parameters of 5-day-old female *Atg7*^*d77*^ mutant flies with MB-specific expression of *Atg7* (*Atg7*^*d77*^*;vt30559*-Gal4>UAS-*Atg7*), as well as of *Atg7*^*d77*^,UAS-*Atg7*/+ genetic controls and *w*^*1118*^ wild-type flies. Sleep data was acquired and averaged over 3 consecutive days. (**E**) Average sleep profiles over 24 h (Zeitgeber time 0–24) plotted in 30 min bins, including 12 h light (ZT0-ZT12, daytime) and 12 h darkness (ZT12-ZT24, nighttime). (**F**) Averaged daily sleep amounts in mins over 24 h, during daytime and nighttime. *n* = 22–45 flies per genotype pooled from two independent experiments. 24 h ****p < 0.0001; Daytime ****p < 0.0001; Nighttime *p = 0.0348, ****p < 0.0001; ns not significant. One-way ANOVA with Tukey’s post hoc test for multiple comparisons. Data presented as mean ± SEM. Source data are available online for this figure.

Considering the MBs central role in sleep regulation (Joiner et al, 2006; Pitman et al, 2006; Sitaraman et al, 2015), and our observation that early MB-specific autophagy impairment triggers accelerated brain-wide PreScale-like synaptic remodeling (Fig. 1A–C’), we asked whether sleep homeostasis during early aging is directly modulated by MB-intrinsic proteostatic disruptions. Indeed, 15 days old female flies with MB-specific *Atg5* knockdown (*vt30559*-Gal4>UAS-*Atg5*-RNAi) exhibited significantly increased total amounts of daily sleep, including both daytime and nighttime sleep, compared to age- and sex-matched controls (Fig. 2A,B).

Moreover, *Atg5* knockdown in the MB also enhanced nighttime sleep in the context of the chronic short-sleeping mutant sleepless (Koh et al, 2008) (*sss*^*P1*^; *vt30559*-Gal4>UAS-*Atg5*-RNAi), which we used as a sensitized genetic background (Fig. 2C,D). This result places MB autophagy function upstream of a known core sleep regulator and suggests that proteostasis within the MB contributes to sleep control at a high hierarchical level of the sleep homeostat.

To test whether restoring autophagy selectively within the MB is sufficient to influence sleep behavior, we examined isogenized *Atg7* null mutants (*Atg7*^*d77*^), which systemically lack autophagy (Juhász et al, 2007). Consistent with prior reports showing increased sleep upon global loss of neuronal autophagy (Bedont et al, 2021), *Atg7*^*d77*^ null mutants showed robust sleep elevation relative to wildtype *w*^*1118*^ controls (Fig. 2E,F). Notably, re-expression of *Atg7* specifically within the 4000 MB KCs (*Atg7*^*d77*^;*vt30559*-Gal4>UAS-*Atg7*) significantly reduced both daytime and nighttime sleep towards wild-type sleep levels (Fig. 2E,F).

Together, these findings demonstrate that MB autophagy exerts bidirectional control over sleep regulation during early aging and highlight its instructive role in a resilience-associated behavioral program.

### MB-specific knockdown of *Atg5* modestly enhances longevity and preserves oxidative stress resistance

**Figure 3:**
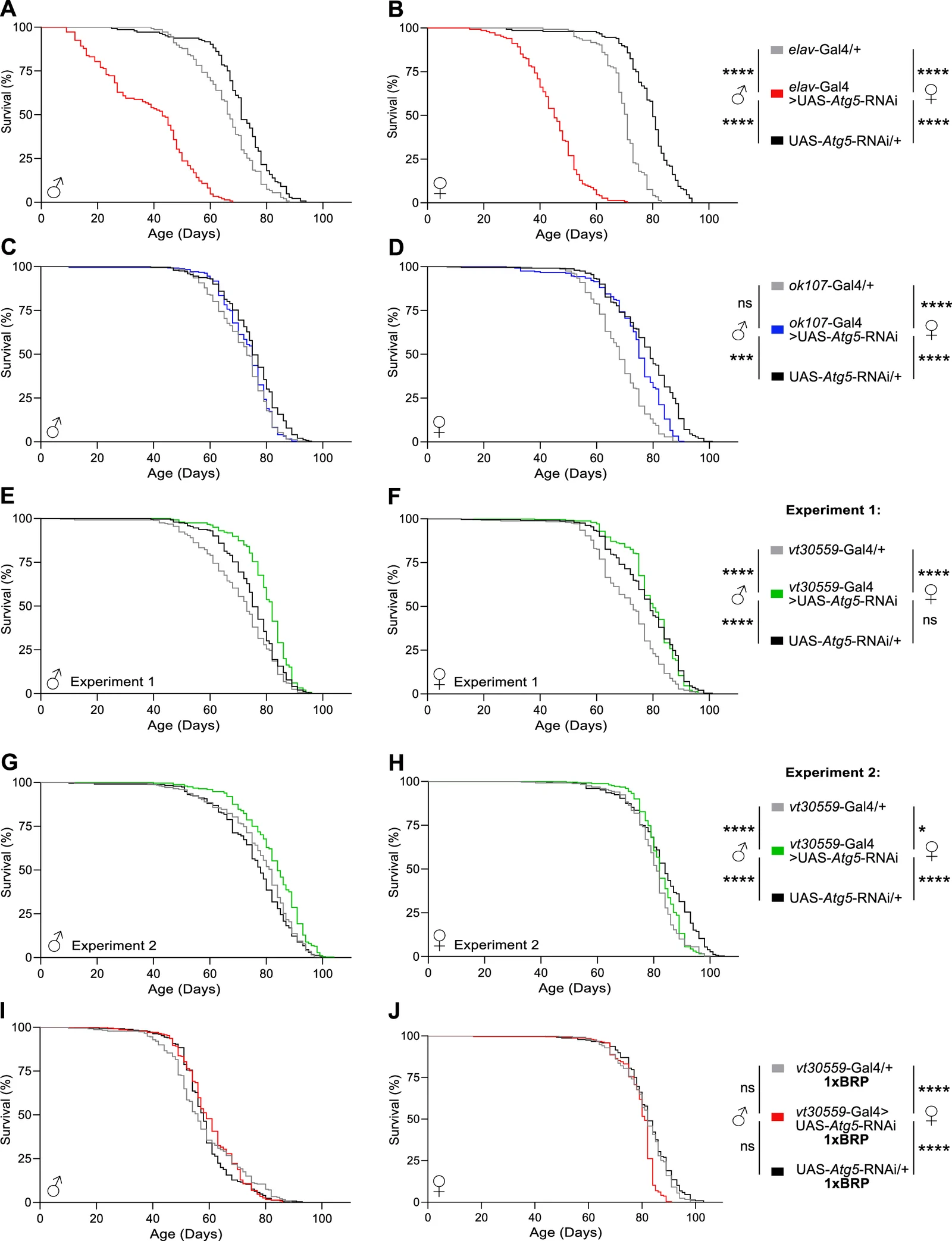
Improved survival of flies with MB-specific *Atg5* knockdown requires adequate BRP levels. (**A**, **B**) Kaplan–Meier survival curves of male (**A**) and female (**B**) flies with pan-neuronal *Atg5* knockdown (*elav*-Gal4>UAS-*Atg5*-RNAi, red curve) compared to *elav*-Gal4/+ and UAS-*Atg5*-RNAi/+ control flies. *n* = 144–148 flies per genotype and sex. Pairwise log-rank (Mantel–Cox) test comparison. ****p < 0.0001. (**C**,** D**) Kaplan–Meier survival curves of male (**C**) and female (**D**) flies with MB-specific *Atg5* knockdown (*ok107*-Gal4>UAS-*Atg5*-RNAi, blue curve) compared to *ok107*-Gal4/+ and UAS-*Atg5*-RNAi/+ control flies. *n* = 242–247 flies per genotype and sex. Pairwise log-rank (Mantel–Cox) test comparison. ***p = 0.0003, ****p < 0.0001, ns not significant. (**E**,** F**) Kaplan–Meier survival curves of male (**E**) and female (**F**) flies with MB-specific *Atg5* knockdown (*vt30559*-Gal4>UAS-*Atg5*-RNAi, green curve) compared to *vt30559*-Gal4/+ and UAS-*Atg5*-RNAi/+ control flies. *n* = 240–248 flies per genotype and sex. Pairwise log-rank (Mantel–Cox) test comparison. ****p < 0.0001, ns not significant. The UAS-*Atg5*-RNAi/+ controls in (**C**, **D**) are the same as in (**E**, **F**). (**G**,** H**) Kaplan–Meier survival curves of male (**G**) and female (**H**) *vt30559*-Gal4>UAS-*Atg5*-RNAi flies compared to *vt30559*-Gal4/+ and UAS-*Atg5*-RNAi/+ controls. Experiment performed with independent cohorts of flies from the ones used in (**E**, **F**). *n* = 236–254 flies per genotype and sex. Pairwise log-rank (Mantel–Cox) test comparison. *p = 0.0378, ****p < 0.0001, ns not significant. (**I**,** J**) Kaplan–Meier survival curves of male (**I**) and female (**J**) flies expressing the same combinations of transgenes as the genotypes in (**G**, **H**), but additionally carrying the *brp*^*c04298*^ null allele in heterozygosity to reduce *brp* gene dosage by 50% (referred to as 1xBRP). *n* = 234–251 flies per genotype and sex. Pairwise log-rank (Mantel–Cox) test comparison. ****p < 0.0001, ns not significant. Source data are available online for this figure.

We next asked whether the MB-triggered resilience response, specifically the accelerated onset of PreScale during early aging (Fig. 1A–C’) and the associated changes in sleep patterns upon MB-specific autophagy impairment (Fig. 2A,B), might be able to compensate for the inherently detrimental loss of autophagic degradation and proteostasis in the MB. Loss of autophagy is per se expected to shorten lifespan and to increase the sensitivity to additional oxidative stress (Juhász et al, 2007; Komatsu et al, 2006; Komatsu et al, 2007; Kuijpers et al, 2021; Maruzs et al, 2019). As anticipated, pan-neuronal knockdown of *Atg5* (*elav*-Gal4>UAS-*Atg5*-RNAi) significantly reduced lifespan in both male and female flies compared to genetic controls (Fig. 3A,B). Similarly, isogenized *Atg7* null mutants (*Atg7*^*d77*^), which systemically lack autophagy, displayed drastically reduced survival compared to wildtype controls (*w*^*1118*^) (Appendix Fig. S2A,B), consistent with previous reports (Juhász et al, 2007). However, when we restored autophagy function in *Atg7*^*d77*^ mutant fly brains, either specifically within the 4000 MB KCs (*Atg7*^*d77*^;*vt30559*-Gal4>UAS-*Atg7*) or pan-neuronally (*Atg7*^*d77*^;*R27E08*-Gal4>UAS-*Atg7*) by overexpression of *Atg7*, the survival of male (Appendix Fig. S2A) and female (Appendix Fig. S2B) flies was significantly improved compared to *Atg7*^*d77*^;UAS-*Atg7*/+ mutant control flies. Together, these results highlight the importance of neuronal autophagy for lifespan regulation and indicate a particularly strong contribution of MB-intrinsic autophagy in this context.

Unexpectedly in this regard, MB-specific *Atg5* knockdown using the strongly MB-targeting *ok107*-Gal4 driver line (Aso et al, 2009) did not compromise longevity (Fig. 3C,D). Instead, we observed a mild trend toward increased survival in males (Fig. 3C) and a modest but significant lifespan extension in females relative to *ok107*-Gal4/+ controls (Fig. 3D), although both sexes remained slightly shorter lived than the UAS-*Atg5*-RNAi/+ controls. Intriguingly, when autophagy was impaired using the strong and highly KC-specific *vt30559*-Gal4 driver line (Plaçais et al, 2017), median survival of male flies with MB-specific autophagy impairment was even significantly extended by several days compared to both genetic controls (Fig. 3E). Female *vt30559*-Gal4>UAS-*Atg5*-RNAi flies also outlived *vt30559*-Gal4/+ controls and showed a positive survival trend relative to UAS-*Atg5*-RNAi/+ control flies, particularly between day 60 and day 80 of age (Fig. 3F). These findings indicate that local impairment of MB autophagy engages protective or compensatory responses that support overall organismal survival.

**Figure EV1:**
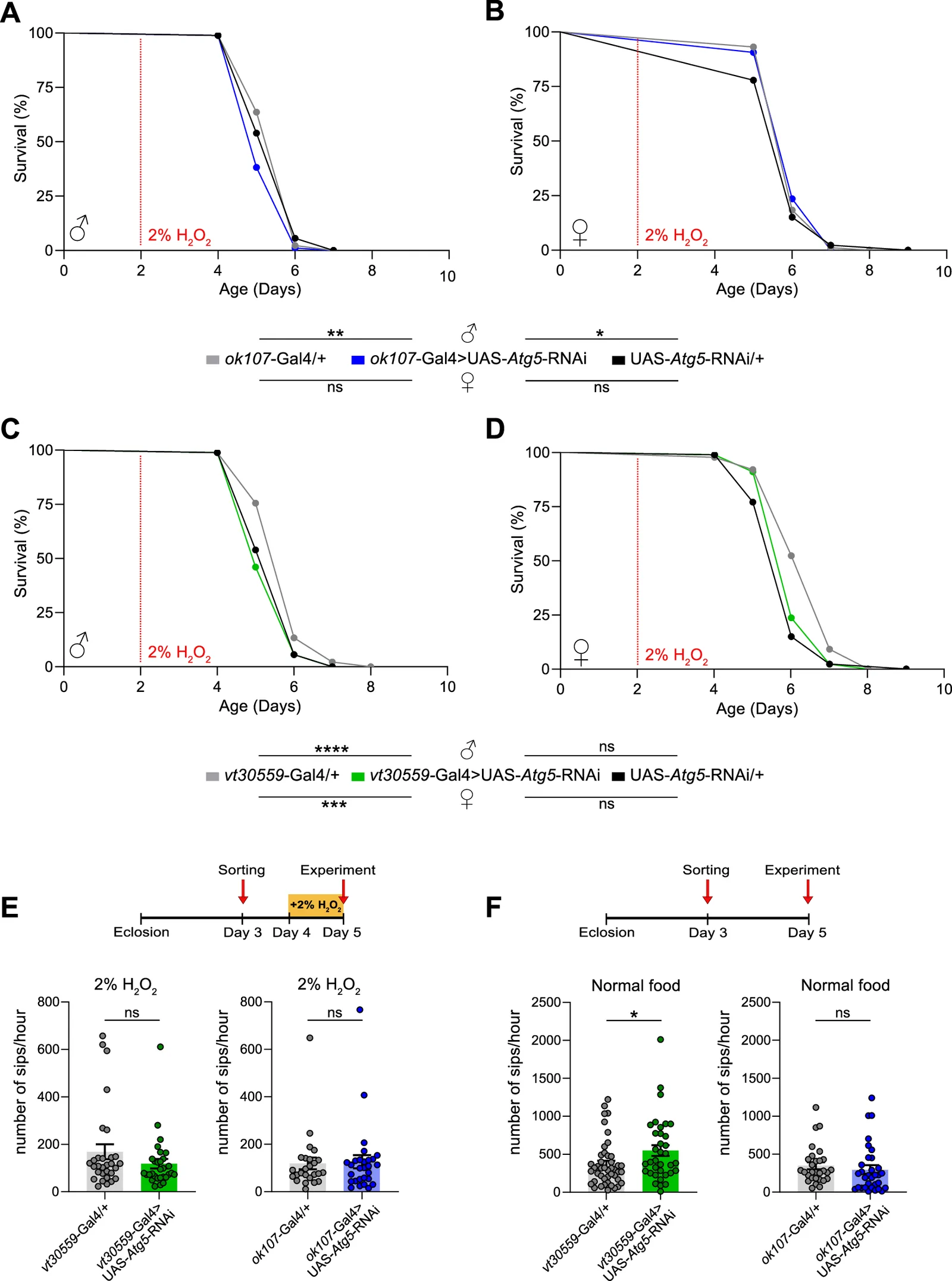
Related to Fig. 3, MB-specific *Atg5* knockdown does not attenuate H_2_O_2_ resistance or feeding behavior. (**A**,** B**) Kaplan–Meier survival curves of male (**A**) and female (**B**) flies with MB-specific *Atg5* knockdown (*ok107*-Gal4>UAS-*Atg5*-RNAi, blue curve) compared to *ok107*-Gal4/+ and UAS-*Atg5*-RNAi/+ control flies continuously exposed to 2% H_2_O_2_ from day 2 onwards. *n* = 85–89 flies per genotype and sex. Pairwise log-rank (Mantel–Cox) test comparison. *p = 0.0180, **p = 0.0011, ns not significant. (**C**,** D**) Kaplan–Meier survival curves of male (**C**) and female (**D**) flies with MB-specific *Atg5* knockdown (*vt30559*-Gal4>UAS-*Atg5*-RNAi, green curve) compared to *vt30559*-Gal4/+ and UAS-*Atg5*-RNAi/+ control flies continuously exposed to 2% H_2_O_2_ from day 2 onwards. *n* = 87–90 flies per genotype and sex. The UAS-*Atg5*-RNAi/+ controls in (**A**, **B**) are the same as in (**C**, **D**). Pairwise log-rank (Mantel–Cox) test comparison. ***p = 0.0003, ****p < 0.0001, ns not significant. (**E**) Number of sips/hour of 5-day-old female *vt30559*-Gal4>UAS-*Atg5*-RNAi and *ok107*-Gal4>UAS-*Atg5*-RNAi flies compared to their respective age- and sex-matched controls (*vt30559*-Gal4/+ and *ok107*-Gal4/+). Flies were sorted at 3 days of age and transferred 24 h before the experiment onto 2% H_2_O_2_ containing sucrose solution. During the experiment, consumption of 2% H_2_O_2_-containing food solution was automatically assessed in FlyPad arenas. *n *= 25–30 flies per genotype pooled from two independent experiments. Mann–Whitney test. ns not significant. (**F**) Number of sips/hour of 5-day-old female *vt30559*-Gal4>UAS-*Atg5*-RNAi and *ok107*-Gal4>UAS-*Atg5*-RNAi flies compared to their respective age- and sex-matched controls (*vt30559*-Gal4/+ and *ok107*-Gal4/+). Flies were sorted at 3 days of age and the consumption of food solution containing 1% Agar, 5% yeast and 5% sucrose was automatically assessed in FlyPad arenas. *n* = 29–45 flies per genotype pooled from three independent experiments. Mann–Whitney test. *p = 0.0270, ns not significant. Data presented as mean ± SD. Source data are available online for this figure.

To assess whether MB-specific autophagy impairment also affects systemic stress resilience, we subjected *ok107*-Gal4>UAS-*Atg5*-RNAi and *vt30559*-Gal4>UAS-*Atg5*-RNAi flies, along with age-matched controls, to chronic oxidative stress (2% H₂O₂ treatment). Despite impaired proteostasis in the central MB, both male and female flies maintained oxidative stress resistance at levels indistinguishable from controls (Fig. EV1A–D). Importantly, MB-autophagy impaired flies showed no pronounced differences in food intake compared to controls, as measured by the number of sips per hour in an automated FlyPad assay (Itskov et al, 2014), either during exposure to 2% H₂O₂ (Fig. EV1E) or under normal feeding conditions (Fig. EV1F). These results exclude altered feeding behavior or major metabolic disturbances, such as starvation or overfeeding, as confounding factors underlying the observed survival and sleep phenotypes.

Taken together, these findings show that MB-specific autophagy impairment enhances organismal resilience, as reflected by increased longevity and preserved oxidative stress resistance. This suggests that autophagy impairment within the MB triggers adaptive, potentially brain-wide, mechanisms.

Given the transient increase in BRP abundance during early aging (Fig. 1), we next tested whether adequate PreScale levels are required to promote this resilience phenotype. To this end, we reduced *brp* gene dosage by introducing a heterozygous *brp* null mutation (*brp*^*c04298*^) into *vt30559*-Gal4>UAS-*Atg5-*RNAi flies. Consistent with our previous observations (Fig. 3E,F), MB-autophagy impaired male flies with normal BRP levels (2xBRP) again showed a significant lifespan extension, while females displayed a more modest effect (Fig. 3G,H). Strikingly, this longevity benefit was completely abolished in males upon BRP reduction to half dose (1xBRP) (Fig. 3I) while survival of females was even significantly attenuated (Fig. 3J), indicating that adequate BRP levels are indeed required for the pro-survival effect.

Together, these findings identify MB-autophagy as a central regulator of the timing and magnitude of brain-wide PreScale activation. Under conditions of autophagy impairment, the MB triggers an accelerated PreScale response during early aging that promotes resilience and enhances survival, whereas reduced BRP dosage prevents sustained activation and abolishes its protective effect. These results establish a mechanistic link between local autophagy function in the MB and organismal resilience, mediated by temporally-controlled brain-wide remodeling of presynaptic active zones.

### Temporally-controlled activation of PreScale during early aging is sufficient to promote longevity

**Figure 4:**
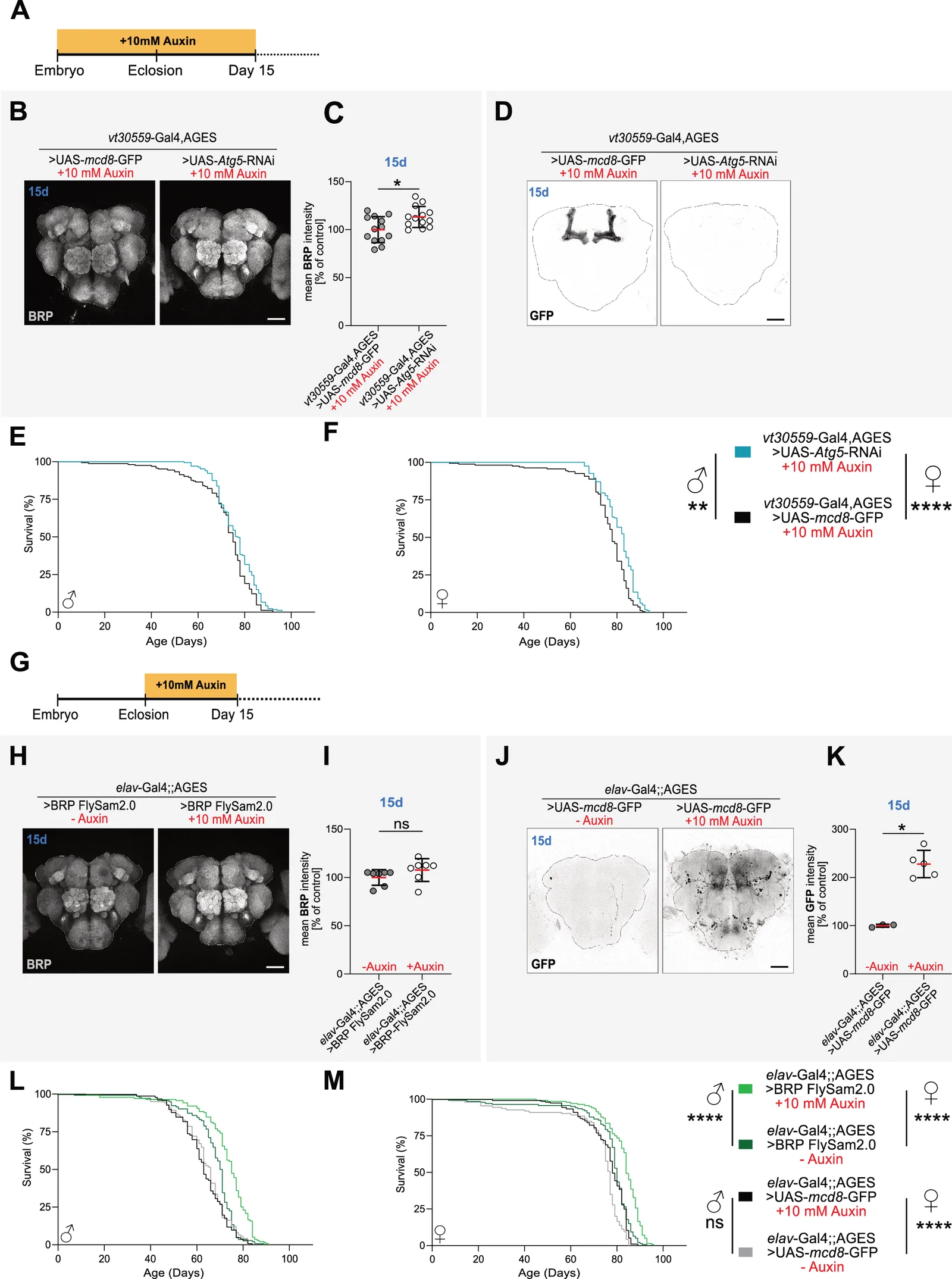
Temporally-controlled activation of PreScale restricted to early aging is sufficient to promote longevity. (**A**) Schematic representation of the time window for 10 mM auxin supplementation, inducing knockdown of *Atg5*, or expression of *mcd8*-GFP as a control, within the MB under *vt30559*-Gal4 control. (**B**–**D**) Auxin-induced knockdown of *Atg5* in the MB during development until day 15 of adult life results in early PreScale activation. (**B**) Representative confocal max projections of adult brains of *vt30559*-Gal4,AGES > UAS-*Atg5*-RNAi and control *vt30559*-Gal4,AGES > UAS-*mcd8*-GFP female flies at 15 d of age after supplementation with 10 mM auxin during larval development and for the first 15 days post-eclosion. Brains were immunostained against BRP^Nc82^; Scale bar = 50 μm. (**C**) Measured whole brain mean BRP intensities for both groups were normalized to the respective *vt30559*-Gal4,AGES > UAS-*mcd8*-GFP control group. *n* = 13 adult brains per genotype, pooled from two independent experiments; unpaired two-tailed *t*-test. *p = 0.0113. (**D**) Representative confocal max projections of adult brains of *vt30559*-Gal4,AGES > UAS-*Atg5*-RNAi and control *vt30559*-Gal4,AGES > UAS-*mcd8*-GFP female flies at 15 days of age after supplementation with 10 mM auxin during larval development and for the first 15 days post-eclosion, illustrating the mcd8-GFP expression pattern within the MB lobes under *vt30559*-Gal4 control. Scale bar = 50 μm. (**E**,** F**) Kaplan–Meier survival curves of male (**E**) and female (**F**) flies with auxin-induced MB-specific *Atg5* knockdown (*vt30559*-Gal4,AGES > UAS-*Atg5*-RNAi, blue curve) compared to *vt30559*-Gal4,AGES > UAS-*mcd8*-GFP controls treated with the same auxin supplementation protocol. 10 mM auxin was supplemented throughout development until day 15 post-eclosion. *n* = 161–167 flies per genotype and sex. Pairwise log-rank (Mantel–Cox) test comparison. **p = 0.028 and ****p < 0.0001. (**G**) Schematic representation of auxin supplementation protocol for post-developmental, pan-neuronal BRP overexpression in *elav*-Gal4;;AGES > BRP FlySam2.0 flies during the first 15 days post-eclosion. (**H**) Representative confocal max projections of adult brains of *elav*-Gal4;;AGES > BRP FlySam2.0 female flies at 15 days of age ± 10 mM auxin supplementation. Brains were immunostained against BRP^Nc82^. Scale bar = 50 μm (**I**) Measured whole brain mean BRP intensities for both groups were normalized to the *elav*-Gal4;;AGES > BRP FlySam2.0 control group without auxin supplementation. *n* = 7 adult brains per genotype; Mann–Whitney test. (**J**) Representative confocal max projections of adult brains of *elav*-Gal4;;AGES > UAS-*mcd8*-GFP female flies at 15 days of age ± 10 mM auxin supplementation, illustrating endogenous fluorescent signal of GFP upon auxin supplementation. Scale bar = 50 μm. (**K**) Measured whole brain mean GFP intensities for both groups were normalized to the *elav*-Gal4;;AGES > UAS-*mcd8*-GFP control group without auxin supplementation. *n* = 3–5 adult brains per genotype; Mann–Whitney test. *p = 0.0357. (**L**,** M**) Kaplan–Meier survival curves of male (**L**) and female (**M**) flies with auxin-dependent pan-neuronal overexpression of BRP (*elav*;;AGES > BRP FlySam2.0) or *mcd8*-GFP (*elav*;;AGES > UAS-*mcd8*-GFP) -/+ auxin treatment. 10 mM auxin was supplemented between day 1 and day 15 post-eclosion. *n* = 100–171 flies per genotype and sex. Pairwise log-rank (Mantel–Cox) test comparison. ****p < 0.0001, ns not significant. Data presented as mean ± SD. Source data are available online for this figure.

To further test whether early-life activation of PreScale contributes to resilience and longevity of MB-autophagy impaired flies, we used the Auxin-inducible Gene Expression system (AGES) (McClure et al, 2022) to temporally restrict the genetic manipulation of MB-autophagy. Post-developmental, Auxin-induced expression of *Atg5*-RNAi in the MB (*vt30559*-Gal4,AGES > UAS-*Atg5*-RNAi) did not result in apparent impairment of protein degradation within the MB KCs at 15 days of age as assessed by Ref(2)P/P62 immunostaining (Appendix Fig. S3). While this may reflect a slow turnover rate of the ATG5 protein, consequently limiting the efficacy of the *Atg5*-RNAi knockdown, we subsequently extended the Auxin supplementation period, starting from larval development until day 15 post-eclosion (Fig. 4A). However, larval Auxin supplementation significantly extended developmental timing in our experimental setup, consistent with previous reports (Fleck et al, 2024), thereby precluding a direct within-genotype ± Auxin comparison of *vt30559*-Gal4,AGES > UAS-*Atg5*-RNAi flies in this context. We therefore decided to use flies expressing an independent UAS-*mcd8*-GFP transgene (*vt30559*-Gal4,AGES > UAS-*mcd8*-GFP), inserted at the same genomic landing site as the UAS-*Atg5*-RNAi transgene, as the experimental control group, and subjected them to the identical developmental Auxin supplementation protocol (Fig. 4A). Importantly, MB-specific knockdown of *Atg5* during this early time window fully recapitulated the premature activation of PreScale at 15 days of age (Fig. 4B–D) in this context, and was sufficient to extend lifespan in both male and female flies (Fig. 4E,F) compared to the isogenic *vt30559*-Gal4,AGES > UAS-*mcd8*-GFP control group.

To further assess whether early-life PreScale activation itself is sufficient to promote longevity independent of MB-autophagy impairment, we next induced BRP expression post-developmentally and pan-neuronally during the first 15 days of adulthood using the same AGES approach (*elav*-Gal4;;AGES > BRP FlySam2.0) (Fig. 4G). For this purpose, we employed a newly generated FlySAM2.0 vector (Jia et al, 2018), which combines a UAS-controlled dCas9 variant with a *brp* promoter-specific gRNA for transcriptional activation of the *brp* gene locus (hereafter referred to as BRP FlySam2.0; see also ‘Methods’ section for details). This genetic approach robustly increased BRP levels at the larval neuromuscular junction (*ok6*-Gal4>BRP FlySam2.0; Appendix Fig. S4A,B) as well as in the adult brain upon pan-neuronal expression (*elav*-Gal4>BRP FlySam2.0; Appendix Fig. S4C,D). The post-developmental supplementation of 10 mM Auxin (day 1–15 post-eclosion; Fig. 4G) induced a modest BRP upregulation at 15 days of age (*elav-*Gal4;;AGES > BRP FlySam2.0; Fig. 4H,I), and also increased mcd8-GFP expression in the parallel control group (*elav-*Gal4;;AGES > UAS-*mcd8*-GFP; Fig. 4J,K). Despite the rather mild BRP increase (Fig. 4H,I), this temporally restricted activation of PreScale was sufficient to significantly enhance lifespan compared to isogenic controls without Auxin treatment (Fig. 4L,M). Importantly, Auxin supplementation did not produce comparable pro-longevity effects in *elav*-Gal4;;AGES > UAS-*mcd8*-GFP control flies (Fig. 4L,M). Although a certain difference in survival was observed in females (Fig. 4M), these results indicate that the lifespan extension observed in *elav*-Gal4;;AGES > BRP FlySam2.0 flies is not attributable to Auxin supplementation itself, but rather to brain-wide BRP upregulation during early adulthood.

Taken together, these results support the idea that early activation of PreScale is sufficient to extend lifespan, and that this AZ-centered resilience mechanism is central to promote longevity in response to autophagy impairment within the MB.

### Coordinated brain-wide remodeling of presynaptic architecture and protein homeostasis in response to MB autophagy impairment

Our results suggest that PreScale functions as a generic, resilience-mediating plasticity program mobilized by the brain in response to diverse physiological challenges. At the molecular level, this state was previously characterized by increased abundance of key active zone (AZ) proteins such as BRP and Unc13A, while core components of the vesicle fusion machinery, such as Syntaxin-1, remained largely unchanged (Huang et al, 2020). However, a comprehensive molecular analysis of PreScale has been lacking, and the broader regulatory logic, particularly regarding non-AZ components and the levels at which these changes occur (transcriptional, translational, or post-translational), remained unclear.

To address this, we examined how local, MB-specific autophagy impairment alters the presynaptic proteome on a brain-wide level. We performed label-free quantitative proteomics on hand-dissected adult brains from 10-day-old *ok107*-Gal4>UAS-*Atg5*-RNAi flies raised at 29 °C and compared them to age-matched *ok107*-Gal4/+ controls, leaving experimental raising conditions exactly the same as in our previous publication (Bhukel et al, 2019). Across four biological replicates per genotype, we detected ~3500 individual proteins (present in ≥3 of 4 replicates). Of these, 13% (471 proteins) were significantly upregulated, and 5% (172 proteins) significantly downregulated in response to MB-specific *Atg5* knockdown (a detailed list of significantly changed protein hits can be found in Appendix Tables S1 and S2).

**Figure 5:**
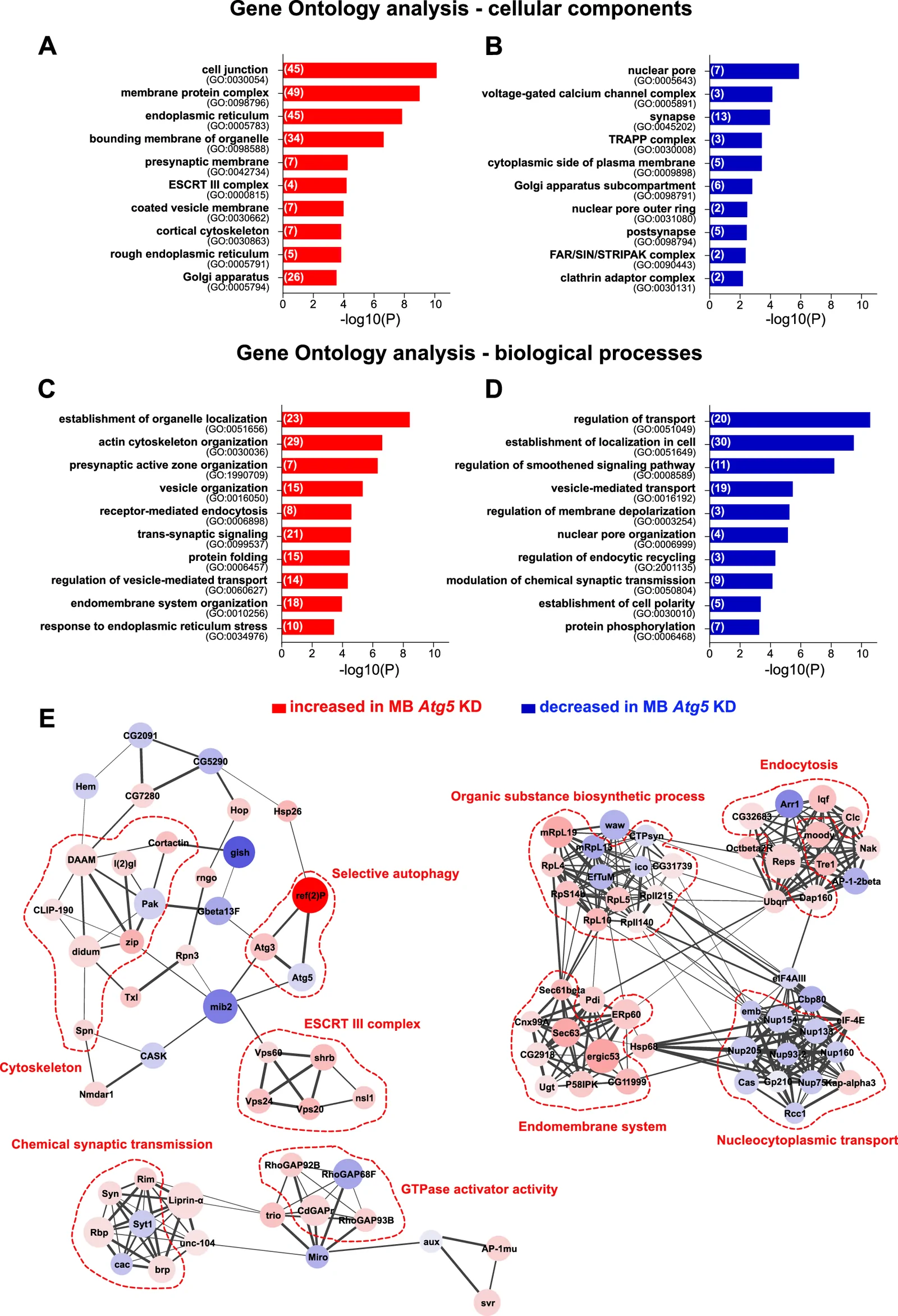
Functional enrichment analysis and interactome of significantly changed proteins in fly brains with MB-specific *Atg5* knockdown. (**A**–**D**) Functional GO enrichment analysis was performed with Metascape, which employs the hypergeometric test and the Benjamini–Hochberg *p* value correction algorithm to identify overrepresented GO terms in a given data set (Zhou et al, 2019). (**A**,** B**) Selected enriched “cellular components” GO terms overrepresented in 10-day-old fly brains with MB-specific knockdown of *Atg5* (ok107-Gal4>UAS-*Atg5*-RNAi) (**A**, increased) or in the age-matched genetic control (*ok107*-Gal4/+) (**B**, decreased). The number of significantly changed proteins identified for each GO term is indicated in brackets. A single protein can be classified into multiple different GO terms. (**C**,** D**) Selected enriched “biological processes” GO terms overrepresented in flies with MB-specific knockdown of *Atg5* (*ok107*-Gal4>UAS-*Atg5*-RNAi) (**C**, increased) or in the wild-type control (*ok107*-Gal4/+) (**D**, decreased). The number of significantly changed proteins identified for each GO term is indicated in brackets. A single protein can be classified into multiple different GO terms. (**E**) Selected PPI clusters identified to be differentially regulated between fly brains with MB-specific *Atg5* knockdown and genetic controls. The size of individual nodes is defined relative to the corresponding −log_10_* p* value, and the color intensity of individual nodes indicates the size of the log_2_ fold change between the effect group and control. The thickness and intensity of individual strings between two nodes indicate the confidence score for a given interaction. The annotated GO terms were directly retrieved from cytoscape. Source data are available online for this figure.

Subsequent unbiased Gene Ontology (GO) functional enrichment analysis using Metascape (Zhou et al, 2019; Fig. 5A–D) revealed a strong enrichment of endoplasmic reticulum (ER)-associated categories among upregulated protein hits (Fig. 5A,C), including the GO terms “endoplasmic reticulum” (GO:0005783), “rough ER” (GO:0005791), “response to ER stress” (GO:0034976), and “protein folding” (GO:0006457). Protein–protein interaction (PPI) analysis further confirmed that ER and endomembrane-associated proteins formed distinct and coherent functional modules (“endomembrane system”; Fig. 5E).

Beyond ER-related signatures, remodeling of the presynaptic active zone emerged as a particularly prominent and specific feature of the proteomic response to MB-specific autophagy impairment. GO terms linked to presynaptic structure and function were significantly enriched across the differentially expressed protein set (Fig. 5A–D). Among the upregulated categories (Fig. 5A,C) were “presynaptic membrane” (GO:0042734), “presynaptic active zone organization” (GO:1990709), and “trans-synaptic signaling” (GO:0099537), whereas downregulated terms (Fig. 5B,D) included “voltage-gated calcium channel complex” (GO:0005891) and “modulation of chemical synaptic transmission” (GO:0050804).

**Figure 6:**
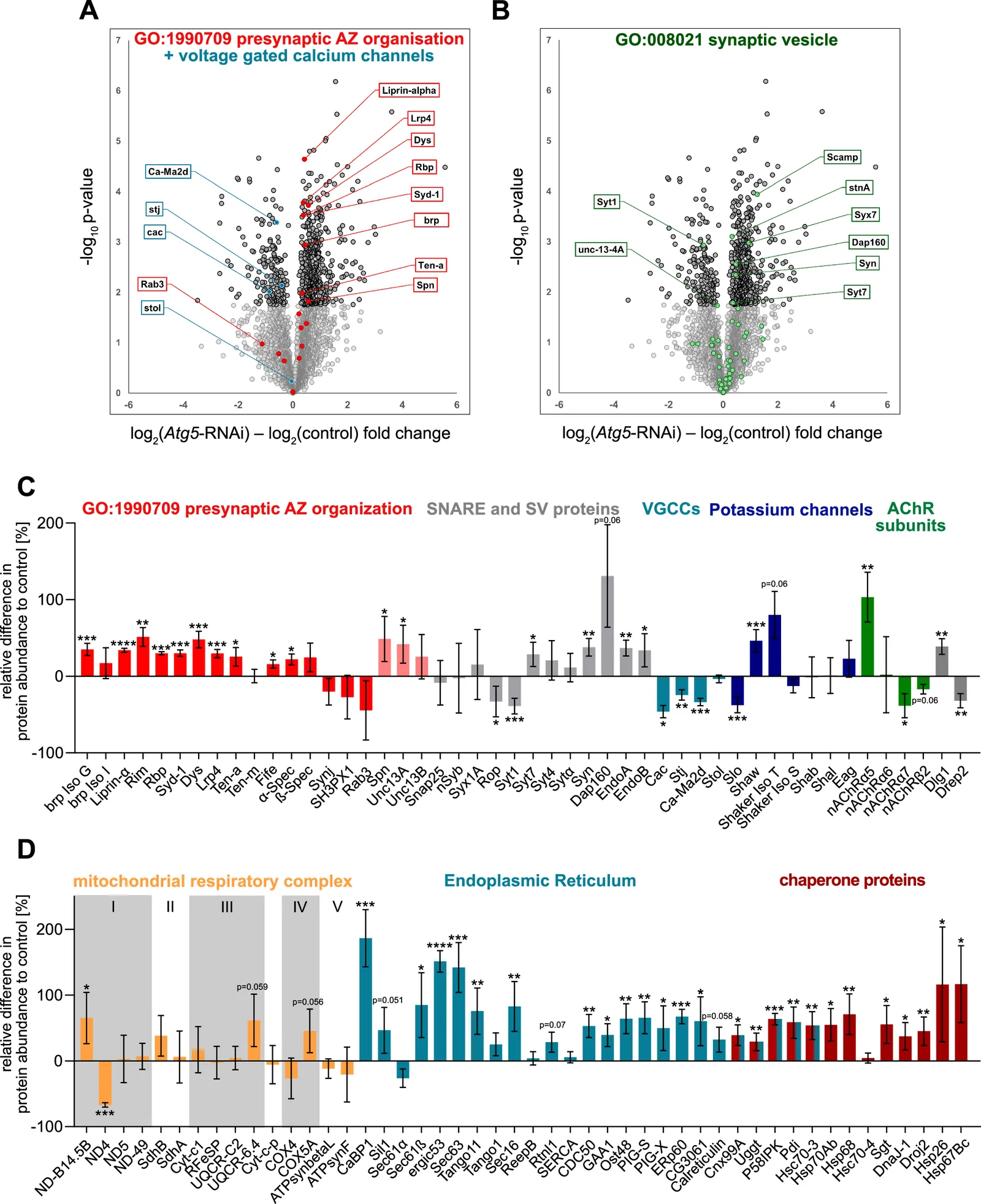
MB-specific *Atg5* knockdown provokes extensive changes in AZ and ER-related protein abundances. Selection of proteomic changes in brains of 10 d old female *ok107*-Gal4>UAS-*Atg5*-RNAi flies compared to age- and sex-matched *ok107*-Gal4/+ controls. (**A**) Volcano plot of all proteins identified in the proteomic data set, highlighting candidates belonging to the GO term “presynaptic AZ organization” (GO:1990709), as well as VGCCs components. The Log_2_ fold change on the x-axis is plotted against the −log_10_* p* value of the two-tailed *t*-test comparison on the y-axis. A −log_10_* p* value cut-off of 1.75 was used to identify significantly changed protein hits (dark gray points), identified proteins below this cutoff are depicted as light gray points. (**B**) Volcano plot highlighting all identified proteins belonging to the GO term “synaptic vesicle” (GO:008021). The log_2_ fold change on the x-axis is plotted against the −log_10_* p* value of the two-tailed *t*-test comparison on the y-axis. A −log_10_* p* value cut-off of 1.75 was used to identify significantly changed protein hits (dark gray points), identified proteins below this cutoff are depicted as light gray points. (**C**) Bar graphs of selected synaptic protein hits, including members of GO:1990709 “presynaptic AZ organization” (red), additional AZ-associated proteins (light red), SNARE complex and synaptic vesicle (SV) components (light gray), VGCCs subunits (light blue), voltage-gated potassium channels (dark blue), acetylcholine receptor (AChR) subunits (green) and postsynaptic proteins (dark gray). Unpaired two-tailed *t*-test. brp Iso G ***p = 0.0009, Liprin-α ****p < 0.0001, Rim **p = 0.0045, Rbp ***p = 0.0002, Syd-1 ***p = 0.0002, Dys ***p = 0.0002, Lrp4 ***p = 0.0002, Ten-a *p = 0.0111, Fife *p = 0.0233, α-Spec *p = 0.0250, Spn *p = 0.0212, Unc13A *p = 0.0265, Rop *p = 0.0432, Syt1 ***p = 0.0008, Syt7 *p = 0.0175, Syn **p = 0.0044, EndoA **p = 0.0015, EndoB *p = 0.0223, Cac *p = 0.0131, Stj **p = 0.0082, Ca-Ma2d ***p = 0.0005, Slo ***p = 0.0006, Shaw ***p = 0.0009, nAChRα5 **p = 0.0012, nAChRα7 *p = 00483, Dlg1 **p = 0.0022, Drep2 **p = 0.0014. (**D**) Bar graphs of selected protein hits, highlighting selected components of all five mitochondrial respiratory complexes (orange), ER-residing proteins (blue) and different classes of chaperone proteins (red). ER-specific chaperone proteins are indicated by two colors. Unpaired two-tailed *t*-test. ND-B14.5B *p = 0.0170, ND4 ***p = 0.0005, CaBP1 ***p = 0.0002, Sec61ß *p = 0.0449, ergic53 ****p < 0.0001, Sec63 ***p = 0.0006, Tango11 **p = 0.0066, Sec16 **p = 0.0069, CDC50 **p = 0.0032, GAA1 *p = 0.0348, Ost48 **p = 0.0024, PIG-S **p = 0.0048, PIG-X *p = 0.0356, ERp60 ***p = 0.0002, CG3061 *p = 0.0210, Cnx99A *p = 0.0114, Uggt **p = 0.0094, P58IPK ***p = 0.0007, Pdi **p = 0.0036, Hsc70-3 **p = 0.0072, Hsp70Ab *p = 0.0144, Hsp68 **p = 0.0062, Sgt *p = 0.0417, DnaJ-1 *p = 0.0139, Droj2 **p = 0.0074, Hsp26 *p = 0.0399, Hsp67Bc *p = 0.0141. For data presented in (**C**, **D**), LFQ intensities measured for the effect group for each protein were normalized to the mean LFQ intensity of the control group to display differences in protein abundance upon MB-specific *Atg5* knockdown relative to the protein level of the control group. *n* = 3–4 biological replicates (10 brains per replicate) per genotype. Only significant differences are depicted. Data presented as mean ± SD. Source data are available online for this figure.

When focusing specifically on presynaptic proteins (Fig. 6A–C), we observed a robust and consistent upregulation of integral AZ components, including BRP, RBP, RIM, Liprin-α, Syd-1, Unc13A, and Spinophilin (Spn) (Fig. 6A,C). In contrast, abundance of synaptic vesicle (SV)-associated proteins remained largely unaffected (Fig. 6B), and SV-related GO categories were likewise not enriched in the functional enrichment and PPI analysis (Fig. 5). These findings indicate that *Atg5* knockdown within the MB induces a selective and regulated restructuring of AZ protein composition, rather than a broad accumulation of synaptic proteins, supporting the interpretation that AZ-remodeling is a hallmark of the PreScale-like resilience program.

Notably, however, not all presynaptic AZ components followed the same pattern. The voltage-gated calcium channel (VGCC) Cacophony (Cac) and its synapse-specific α2δ subunit straightjacket (stj) were significantly downregulated, while the dendritically localized α2δ subunit stolid (stol) remained unchanged (Fig. 6A,C) (Heinrich and Ryglewski, 2020). This selective reduction of axonal VGCC components points to a targeted reprogramming of presynaptic calcium signaling within axonal compartments, rather than a global reduction in channel expression. Such a shift is likely to decrease the probability of SV release in response to action potentials, tuning synaptic output in an activity-dependent manner. Along those lines, core SNARE proteins (Snap25, nSyb, Syx1A) remained unchanged (Fig. 6C), but the Ca²⁺ sensor Synaptotagmin-1 (Syt1) was moderately downregulated (Fig. 6B,C), reinforcing the idea of dampened release dynamics. On the other hand, several voltage-gated potassium channels, including Shaker (K_v_1) and Shaw (K_v_3), were robustly upregulated (Fig. 6C). Given the opposing effects of calcium and potassium conductance on presynaptic excitability, i.e., facilitating versus restraining neurotransmitter release, respectively, these combined changes suggest an adaptive rebalancing of axonal excitability. Together, these findings point to a coordinated reprogramming of presynaptic terminals at the level of calcium influx and membrane excitability, likely contributing to the protective, low-gain transmission state characteristic of PreScale (Huang et al, 2022).

As already suggested by the GO functional enrichment and PPI analysis (Fig. 5), we also observed increased protein levels of numerous ER-related components in fly brains with MB-specific *Atg5* knockdown (Fig. 6D), and multiple ER-related proteins (ergic53, CaBP1, and ScpX) were identified among the top 20 protein hits with the highest p-value (Appendix Fig. S5). Among the significantly increased hits further were ER-components involved in protein folding (Calnexin99A (Cnx99a), Uggt, P58IPK, Hsc70-3, Pdi), GPI-anchoring (PIG-S), ER-organization (Sec16) and response to ER stress (Hsc70-3; CaBP1, Erp60, Uggt, and Pdi) (Fig. 6D). Of note, we also observed increased levels of diverse classes of chaperone proteins including Hsp40/DNAj co-chaperones (DnaJ-1 and Droj2), Hsp70 chaperones (Hsp68, Hsc70-3, and Hsp70Ab) as well as small Hsps (Hsp26 and Hsp67Bc) (Fig. 6D), indicating increased activity of protein-quality control systems upon impairment of autophagic degradation within the MB. Surprisingly, we could not detect major changes in mitochondria-specific proteins, suggesting that mitochondria might not be a major substrate of neuronal autophagy in this context. A lack of mitochondrial phenotypes had been reported also for murine neurons with conditional knockout of *Atg5* (Kuijpers et al, 2021). Coherently, a directed search for proteins of all 5 complexes of the mitochondrial respiratory electron transport chain revealed no clear tendency towards increased or decreased abundances, with most components being unchanged compared to the control group (exemplary protein hits depicted in Fig. 6D). Furthermore, mitochondria-related GO terms were also not identified among the GO functional enrichment (Fig. 5A–D) and PPI network analyses (Fig. 5E).

**Figure EV2:**
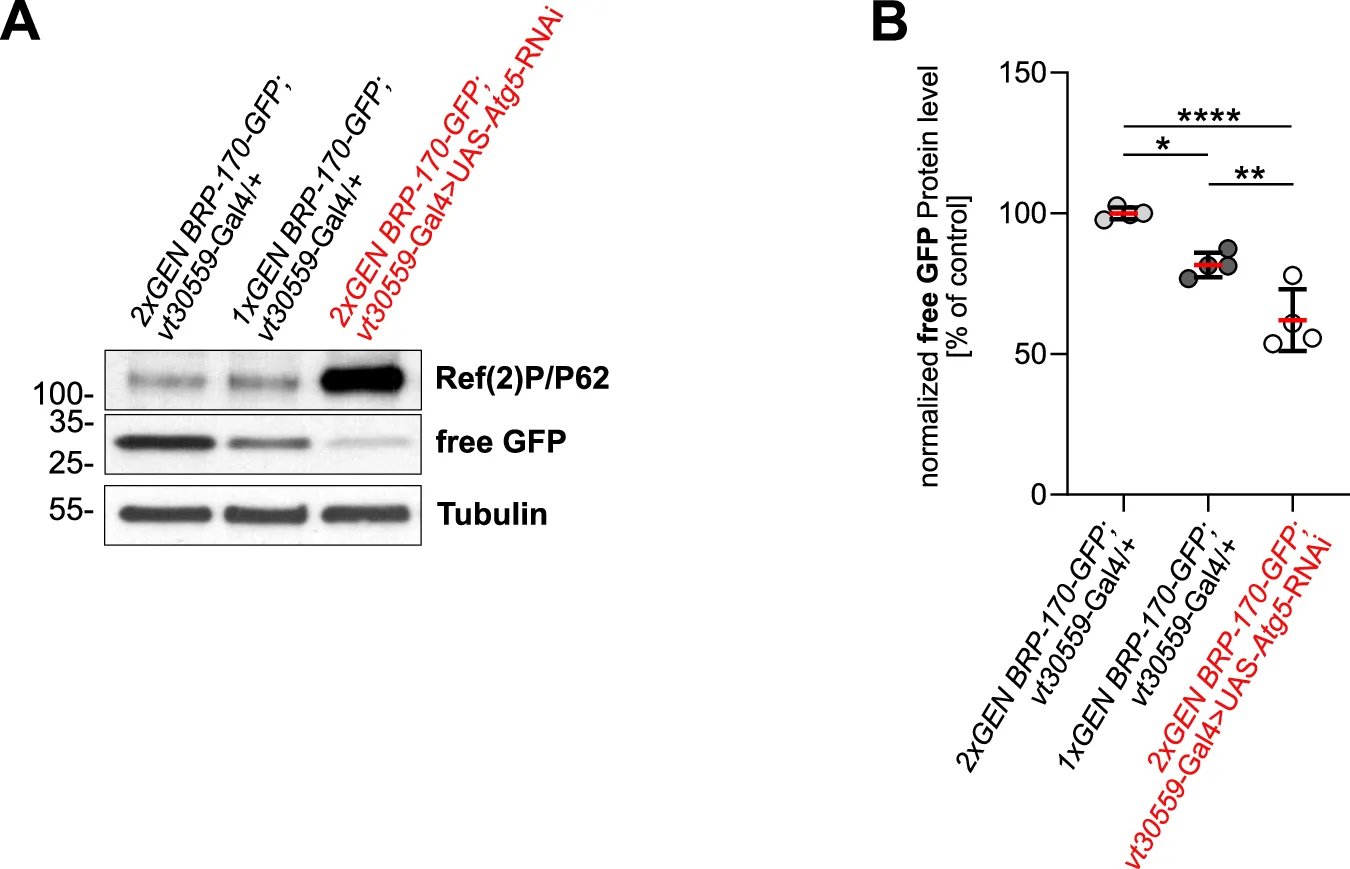
Related to Fig. 6, lysosomal degradation of a GFP-tagged 170 kDa BRP fusion protein is strongly reduced in fly brains with MB-autophagy impairment. (**A**) Representative Western blot of brain homogenates from 5-day-old female flies expressing either one or two additional genomic copies of a GFP-tagged BRP 170 kDa isoform. Control flies additionally carry the *vt30559*-Gal4/+ transgene alone, while in the effect group, UAS-*Atg5*-RNAi is driven exclusively in the MB under control of *vt30559*-Gal4. Membranes were probed against GFP and Ref(2)P/P62, and Tubulin was used as a loading control. An amount equal to two brains was loaded for each lane. (**B**) Quantification of free GFP protein amounts. Measured band intensities were first normalized to the respective Tubulin band intensity, and values of all groups were normalized to the 2xGen BRP-170-GFP;*vt30559*-Gal4/+ control group. *n* = 4 independently prepared Western blot samples run on two separate gels. One-way ANOVA with Tukey’s post hoc test for multiple comparisons. *p = 0.0120, **p = 0.0079, ****p < 0.0001. Data presented as mean ± SD. Source data are available online for this figure.

The observed increase in core presynaptic proteins such as BRP suggests that some AZ components may normally be degraded by autophagy, and thus accumulate upon MB-specific *Atg5* knockdown. Consistent with this idea, BRP has previously been identified as an autophagy substrate in developing neurons (Dutta et al, 2023; Hassan and Hiesinger, 2023). To directly assess BRP degradation dynamics, we performed a GFP conversion assay in which lysosomal processing of GFP-tagged proteins is detected by accumulation of a 27 kDa free GFP fragment in Western blot (Mauvezin et al, 2014; Nagy et al, 2015) (Fig. EV2A). In flies carrying two genomic copies of GFP-tagged BRP 170 kDa isoform (BRP-170-GFP), MB-specific *Atg5* knockdown led to a marked reduction in free GFP levels compared to controls (Fig. EV2A,B).

These results are consistent with the idea that degradation rates of BRP are slowed down upon MB-specific autophagy disruption and likely contribute to its increased abundance under these conditions.

### Extensive proteomic remodeling upon MB-autophagy impairment occurs largely independent of transcriptional changes

To further determine whether these widespread proteomic changes originated at the transcriptional level or instead reflected post-transcriptional regulation, we performed RNA sequencing of fly heads from 10-day-old *ok107*-Gal4>UAS-*Atg5*-RNAi and *ok107*-Gal4/+ control flies. We identified 10262 transcripts, from which only 2.2% (228) were significantly upregulated, and 2.6% (270) were significantly downregulated. Moreover, a cross comparison of proteomic and transcriptomic hits revealed that fewer than 6% of all significantly changed protein hits showed corresponding alterations at the transcript level (Appendix Fig. S6). While some discrepancy could be due to technical limitations, the data suggest that the majority of proteomic remodeling, including that of AZ proteins, VGCC subunits and ER components, is driven primarily by translational control or altered protein turnover, rather than changes in gene expression. Notably, however, two molecular chaperones, Hsp26 and Cnx99A, were transcriptionally upregulated (Appendix Fig. S6) in parallel with their increased protein levels (Fig. 6D), consistent with a selective activation of protective protein quality control programs in response to MB-autophagy impairment.

### MB-specific *Atg5* knockdown results in brain-wide, non-cell autonomous accumulation of Ref(2)P/P62-positive ubiquitinated protein aggregates

**Figure 7:**
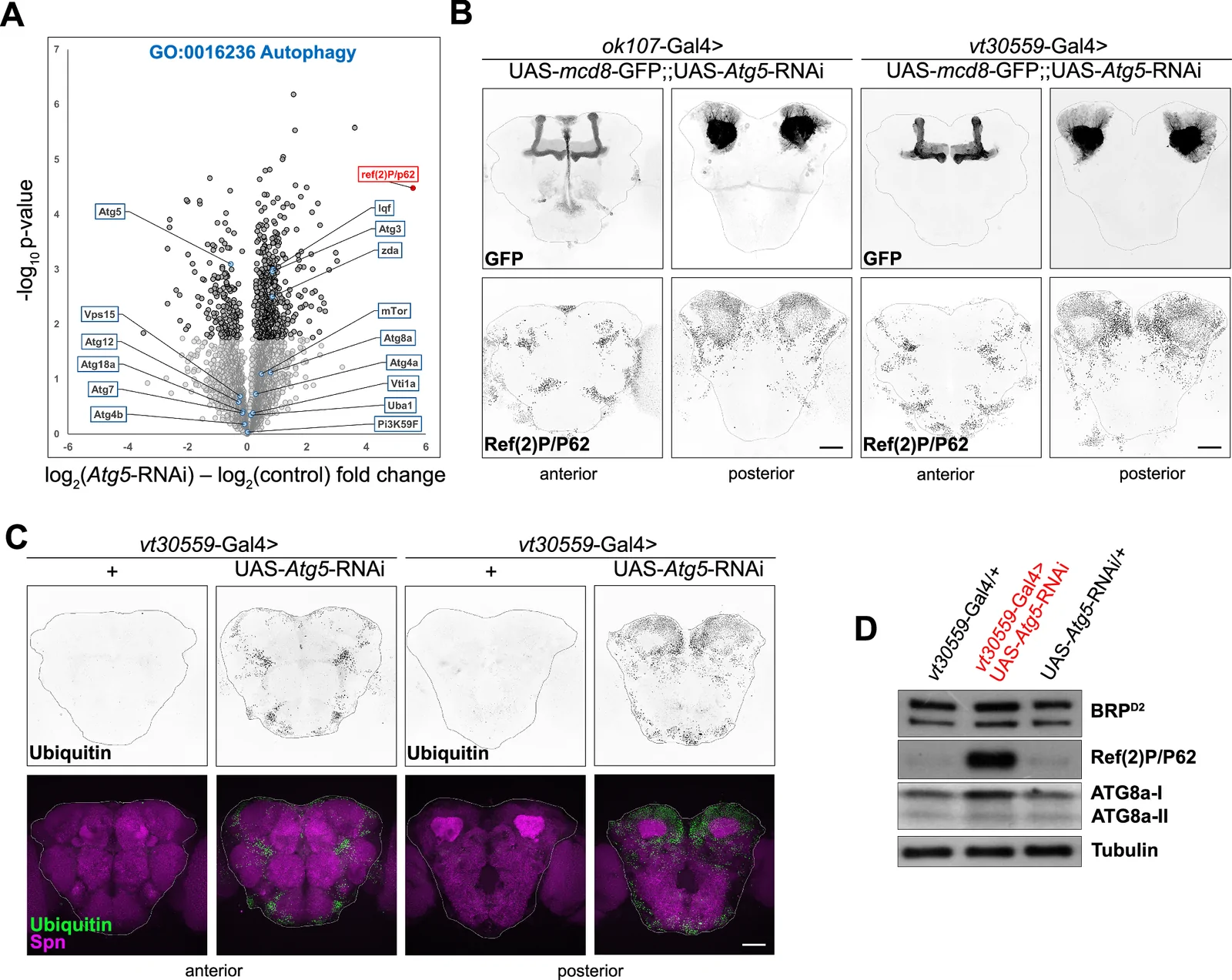
Widespread non-cell autonomous accumulation of autophagic substrates upon MB-restricted *Atg5* knockdown. (**A**) Volcano plot highlighting all proteins belonging to the GO term “autophagy” (GO:0016236) identified in the proteomic data set (Figs. 5 and 6). The Log_2_ fold change between *ok107*-Gal4>UAS-*Atg5*-RNAi effect and *ok107*-Gal4/+ control group is displayed on the x-axis, the −log_10_* p* value of the two-tailed *t*-test comparison is plotted on the y-axis. A −log_10 _*p* value cut-off of 1.75 was used to identify significantly changed protein hits (dark gray points), identified proteins below this cutoff are depicted as light gray points. *n* = 3–4 biological replicates (10 dissected brains per replicate) per genotype. (**B**) Representative confocal max projections of the anterior and posterior side of 5-day-old female fly brains simultaneously expressing a membrane-bound GFP (UAS-*mcd8*-GFP) and an RNAi against *Atg5* (UAS-*Atg5*-RNAi) under the control of either *ok107*-Gal4 or *vt30559*-Gal4. Whole-mount brain immunostaining was performed against Ref(2)P/P62 and BRP^Nc82^, and mcd8-GFP fluorescence was detected without additional antibody labeling. Scale bar = 50 µm. (**C**) Representative confocal max projections of the anterior and posterior side of 5-day-old female fly brains with MB-specific knockdown of *Atg5* (*vt30559*-Gal4>UAS-*Atg5*-RNAi) compared to a genetic control (*vt30559*-Gal4/+). Whole-mount brain immunostaining was performed against Ubiquitin and Spinophilin (Spn). Scale bar = 50 µm. (**D**) Representative Western blot of whole brain homogenates of 5-day-old female flies with MB-specific knockdown of *Atg5* (*vt30559*-Gal4>UAS-*Atg5*-RNAi) and of the two genetic controls (*vt30559*-Gal4/+ and UAS-*Atg5*-RNAi/+). Membranes were probed with antibodies against BRP^D2^, Ref(2)P/P62 and ATG8a, differentiating between the unlipidated (ATG8a-I) and the autophagosome-associated lipidated (ATG8a-II) form. Tubulin was probed as loading control. The amount equal to two brains was loaded per lane. Source data are available online for this figure.

ATG5 is an essential component of the autophagic machinery required for LC3/ATG8a lipidation, and thus is indispensable for phagophore expansion and autophagosome formation (Glick et al, 2010; Iriondo et al, 2023; Mizushima et al, 1998; Yamamoto et al, 2023). In the *Drosophila* brain, pan-neuronal knockdown of *Atg5* leads to a complete loss of lipidated ATG8a-II and causes widespread accumulation of undegraded protein aggregates marked with the selective autophagy receptor Ref(2)P/P62 (Bhukel et al, 2019). In our PPI analysis of brain homogenates from MB-autophagy-impaired *ok107*-Gal4>UAS-*Atg5*-RNAi flies, we identified a small protein cluster annotated as “selective autophagy,” containing mildly decreased ATG5 and strongly increased Ref(2)P/P62 levels (Fig. 5E). However, other autophagy-related GO terms and protein clusters were not identified by our analyses of significantly changed protein hits. A focused search for proteins belonging to the GO term “autophagy” (GO:0016236) confirmed that multiple autophagy key components—including ATG4, ATG7, ATG12, and ATG8—remained unchanged upon MB-specific *Atg5* knockdown compared to controls (Fig. 7A). Importantly however, Ref(2)P/P62 was identified as the most enriched protein in the *ok107*-Gal4>UAS-*Atg5*-RNAi group (Fig. 7A), indicating strong imbalance of autophagic degradation and a drastic accumulation of undegraded protein aggregates.

While a certain degree of Ref(2)P/P62 buildup was anticipated following MB-specific *Atg5* knockdown, the magnitude of this increase was surprising, given that only ~3% of all neurons in the brain (~4000 MB KCs) were genetically targeted. This was also reflected by the only mildly reduced total ATG5 protein levels in whole brain extracts (Figs. 5E and 7A). To further investigate the spatial distribution of Ref(2)P/P62 accumulations across the brain, we co-expressed a UAS-*mcd8*-GFP transgene in combination with the UAS-*Atg5*-RNAi transgene under the control of either *ok107*-Gal4 or *vt30559*-Gal4. This allowed us to visualize Ref(2)P/P62 localization and abundance in relation to the genetically targeted and GFP-labeled MB neurons with reduced *Atg5* expression. Strikingly, numerous large Ref(2)P/P62-positive aggregates were located outside the GFP-labeled MB KCs in both anterior and posterior brain areas (Fig. 7B), indicating a non-cell autonomous accumulation of undegraded protein aggregates and pointing towards either a propagation of autophagy imbalance, or a spreading of autophagic substrates across the brain.

These findings were corroborated by an independent genetic approach, in which a UAS-*Cas9* transgene was expressed under *vt30559*-Gal4 control in flies ubiquitously carrying a guide RNA to induce site-directed DNA double-strand breaks into the *Atg5* gene. This MB-restricted genome editing again resulted in widespread non-cell autonomous Ref(2)P/P62 accumulation (Appendix Fig. S7). As ubiquitination typically precedes Ref(2)P/P62-mediated cargo aggregation and degradation (Cabe et al, 2018; Khaminets et al, 2016; Nezis et al, 2008), we immunostained MB-autophagy impaired fly brains with an anti-ubiquitin antibody and observed numerous ubiquitin-positive puncta outside the MB in similar brain regions as the previously observed non-cell autonomous Ref(2)P/P62 aggregates (Fig. 7C), further confirming the accumulation of undegraded autophagic substrates across the brain. Surprisingly, Western blot analysis of whole brain homogenates revealed that total ATG8a-II levels were not reduced in *vt30559*-Gal4>UAS-*Atg5*-RNAi flies compared to controls (Fig. 7D), suggesting that ATG8a lipidation, and thereby most likely also autophagosome formation, remained globally intact and reinforcing that the *Atg5* knockdown was spatially confined to the MB KCs. Nonetheless, also a modest accumulation of unlipidated ATG8a-I was detected, consistent with cell-autonomous effects of ATG5 loss within MB KCs.

**Figure EV3:**
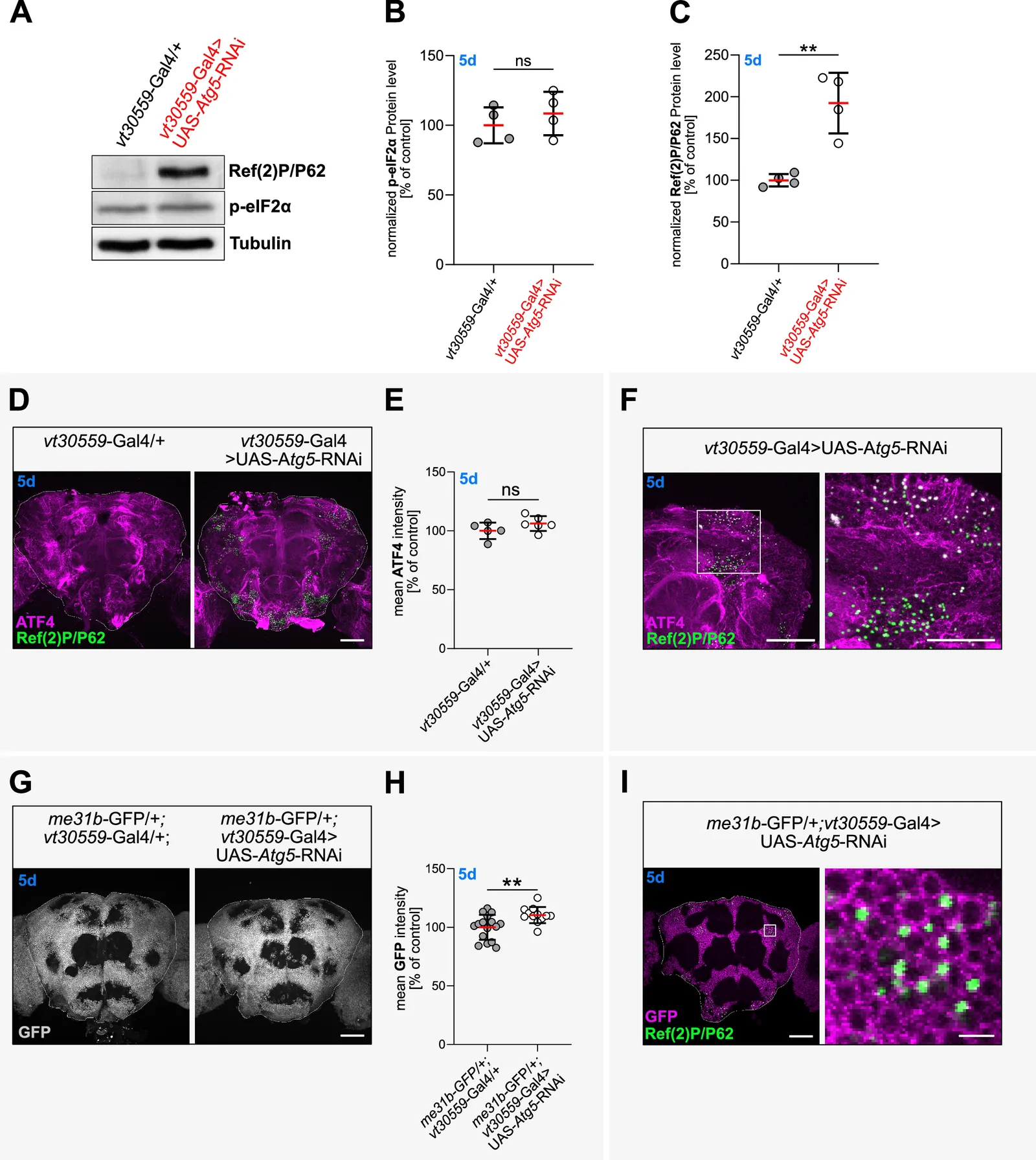
Related to Fig. 7, MB-specific *Atg5* knockdown does not induce global activation of the integrated stress response (ISR). (**A**–**C**) Western blot analysis of Ref(2)P/P62, as well as of Ser51-phosphorylation levels of eIF2α in brain homogenates of 5-day-old female *vt30559*-Gal4>UAS-*Atg5*-RNAi flies in comparison to age- and sex-matched *vt30559*-Gal4/+ controls. (**A**) Representative Western blot displaying protein bands for Ref(2)P/P62, p-eIF2α and Tubulin as loading control. An amount equal to 2 brains was loaded for each lane. (**B**) Quantification of p-eIF2α levels. Band intensities were first normalized to the respective Tubulin band intensity, and all values were subsequently normalized to the *vt30559*-Gal4/+ control group. *n* = 4 independently prepared Western blot samples run together on the same gel; unpaired two-tailed *t*-test. ns not significant. (**C**) Quantification of Ref(2)P/P62 levels. Band intensities were first normalized to the respective Tubulin band intensity, and all values were subsequently normalized to the *vt30559*-Gal4/+ control group. *n* = 4 independently prepared Western blot samples run together on the same Gel; unpaired two-tailed *t*-test. **p = 0.0025. (**D**–**F**) Confocal microscopy analysis of ATF4 and Ref(2)P/P62 in adult brains of 5-day-old female *vt30559*-Gal4>UAS-*Atg5*-RNAi flies and *vt30559*-Ga4/+ controls. (**D**) Representative confocal max projections of ATF4 and Ref(2)P/P62. (**E**) Quantification of whole brain mean ATF4 intensities in 5-day-old female flies. Scale bar = 50 μm; *n* = 5–6 adult brains per genotype; unpaired two-tailed *t*-test. ns not significant. (**F**) Representative confocal max projections of the anterior brain of a *vt30559*-Gal4>UAS-*Atg5*-RNAi fly, illustrating Ref(2)P/P62 aggregates with and without co-localization of ATF4. Scale bar left panel = 50 µm; Scale bar right panel = 25 µm. (**G**–**I**) Confocal microscopy analysis of endogenously tagged me31b-GFP in adult brains of 5-day-old female *me31b*-GFP/+;*vt30559*-Gal4>UAS-*Atg5*-RNAi flies and *me31b*-GFP;*vt30559*-Gal4/+ controls. (**G**) Representative confocal max projections of me31b-GFP fluorescent intensity. (**H**) Quantification of whole brain mean me31b-GFP intensities in 5-day-old female flies. Scale bar = 50 μm; *n* = 12–16 adult brains per genotype pooled from two independent experiments; unpaired two-tailed *t*-test. **p = 0.0066. (**I**) Representative images of a single optical section of the anterior brain of a *vt30559*-Gal4>UAS-*Atg5*-RNAi fly, illustrating Ref(2 P)/P62 aggregates without observable accumulation of me31b-GFP signal. Scale bar left panel = 50 µm, Scale bar right panel = 5 µm. Data presented as mean ± SD. Source data are available online for this figure.

Local loss of sensory input in *Drosophila* was recently shown to induce a non-cell autonomous activation of the integrated stress response (ISR), marked by membrane-less cytosolic stress granules containing RNA, the key ISR marker ATF4, and also Ref(2)P/P62 (Shekhar et al, 2025). To test whether our scenario elicits a similar ISR signature, we assessed phosphorylated eIF2α levels as well as ATF4 abundance and localization in 5-day-old flies with MB-specific *Atg5* knockdown (Fig. EV3A–F). Despite robust Ref(2)P/P62 accumulation (Fig. EV3A,C,D), neither phosphorylated eIF2α abundance (Fig. EV3A,B), nor overall ATF4 levels (Fig. EV3D,E) were significantly altered compared to controls. Although ATF4 was detected in a subset of Ref(2)P/P62-positive aggregates (Fig. EV3F), this was not uniformly observed across the brain. Together, these data do not support a general ISR activation upon MB-autophagy impairment and suggest that these aggregates differ from canonical stress granules. Consistently, endogenously GFP-tagged me31b, a translational repressor associated with ribonucleoprotein complexes (Formicola et al, 2021), displayed mildly elevated protein levels in 5-day-old *vt30559*-Gal4>UAS-*Atg5*-RNAi brains (Fig. EV3G,H) but did not accumulate inside Ref(2)P/P62 aggregates (Fig. EV3I), indicating that they are unlikely to represent RNA-containing regulatory granules.

### Non-cell autonomous Ref(2)P/P62 aggregates colocalize with ATG8a and reflect functional but limited degradation outside the MB

**Figure 8:**
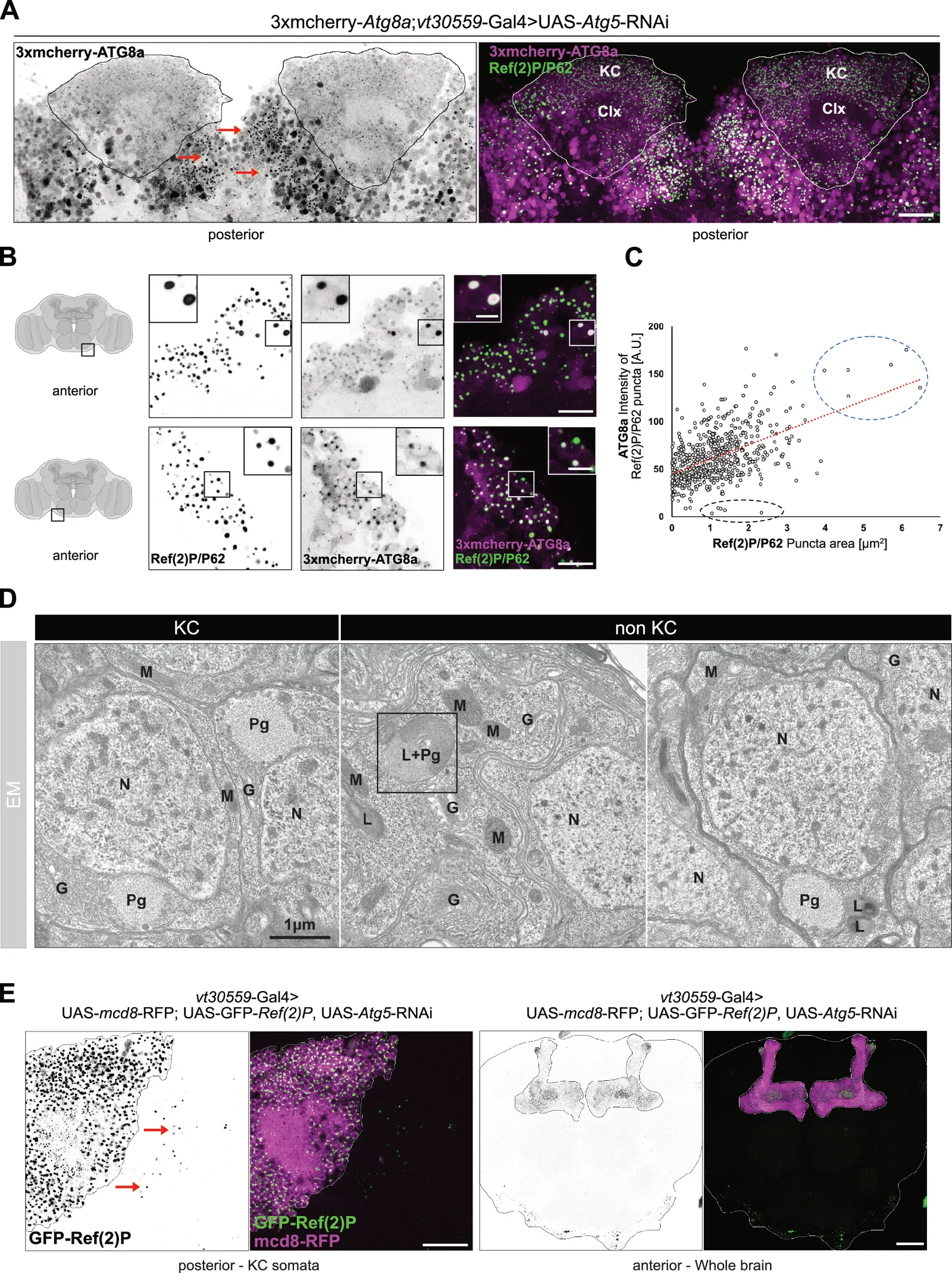
Non-cell autonomous Ref(2)P/P62 aggregates colocalize with ATG8a and are found inside degradative lysosomal compartments. (**A**) Representative confocal max projections of the posterior side of the brain of a female *vt30559*-Gal4>UAS-*Atg5*-RNAi fly, immunostained with an antibody against Ref(2)P/P62 and simultaneously displaying endogenous 3xmCherry-ATG8a fluorescent signal. The MB KC regions (comprising the KC cell bodies (KC) and the Calyx neuropile (Clx)) are indicated by ROIs. Examples of non-cell autonomous 3xmCherry-ATG8a puncta outside the genetically targeted MB region are indicated by red arrows. Scale bar = 25 µm. (**B**) Representative confocal max projections of inferior brain regions close to the suboesophageal ganglion, highlighting the non-cell autonomous accumulation of heterogeneous Ref(2)P/P62 aggregates partially co-positive for 3xmCherry-ATG8a. Zoom-ins highlight large Ref(2)P/P62 puncta strongly co-positive for 3xmCherry-ATG8a (upper row), as well as a Ref(2)P/P62 punctum without 3xmCherry-ATG8a co-localization (lower row). Scale bar = 15 μm, zoom-in scale bar = 5 μm. (**C**) Plot of Ref(2)P/P62 puncta area in relation to the ATG8a intensity inside the Ref(2)P/P62 puncta. Quantification related to representative images in (**B**). The blue circle highlights the largest Ref(2)P/P62 puncta with high intensity mCherry-ATG8a co-labeling (exemplified in zoom-in in (**B**), upper row), gray circles indicate Ref(2)P/P62 aggregates without any mCherry-ATG8a co-label (exemplified in zoom-in in (**B**), lower row). *n* = 606 analyzed Ref(2)P/P62 puncta. (**D**) Representative electron micrographs showing neurons from 5-day-old female fly brains with MB-specific *Atg5* knockdown (*vt30559*-Gal4>UAS-*Atg5*-RNAi). Protein granules (Pg) are present in the perikarya of MB Kenyon cells (KC), but also in other regions of the brain (non-KC). In these regions, lysosomes (L) are frequently observed near protein granules, and in some cases, protein granules are located inside lysosomes (L + Pg; middle panel), likely undergoing degradation. N nucleus, M mitochondrion, G Golgi. Scale bar = 1 µm. (**E**) Representative single section of the posterior side (left panels), and representative max projection of the anterior side (right panels), of 5-day-old female flies expressing UAS-*mcd8*-RFP, UAS-GFP-*Ref(2)P* and UAS-*Atg5*-RNAi specifically within the MB KCs under *vt30559*-Gal4 control. GFP-Ref(2)P fluorescence signal can be detected non-cell autonomously outside the MB KCs (exemplified by red arrows, left panel). Scale bar left panel = 25 µm; Scale bar right panel = 50 µm. Source data are available online for this figure.

Given the lack of evidence for a global ISR activation, we next asked whether MB-specific autophagy impairment induces spatially distinct, cell autonomous versus non-cell autonomous autophagy phenotypes across the brain. Thus, to better separate local (i.e., within the MB) from global, non-cell autonomous autophagy phenotypes, we combined *vt30559*-Gal4 and UAS-*Atg5*-RNAi fly lines with a ubiquitously expressed 3xmCherry-tagged endogenous *Atg8a* reporter (Hegedűs et al, 2016). Within KC cell bodies surrounding the MB calyx, the mCherry-ATG8a signal appeared diffuse and lacked punctate structures, despite the presence of numerous Ref(2)P/P62 aggregates (Fig. 8A), consistent with impaired autophagosome formation following *Atg5* knockdown in the MB. In the MB lobes, mCherry-ATG8a signal was increased but remained diffuse in synaptic regions (Appendix Fig. S8), likely reflecting the accumulation of unlipidated ATG8a-I in presynaptic compartments, in line with the elevated ATG8a-I levels observed by Western blot (Fig. 7D).

Outside the MB, however, in neurons not targeted by *vt30559*-Gal4, we observed large and intense mCherry-ATG8a-positive puncta, which frequently co-localized with Ref(2)P/P62 aggregates and which could reach multiple µm in diameter (Fig. 8A,B). These ATG8a structures were particularly prominent in posterior regions between the calyces (Fig. 8A, red arrows), in anterior areas near the antennal lobes, and in inferior brain regions adjacent to the suboesophageal ganglion (Fig. 8B). The co-localization of mCherry-ATG8a signal with Ref(2)P/P62 aggregates was heterogeneous: Some of the largest Ref(2)P/P62 puncta also displayed the highest intensity for mCherry-ATG8a (Fig. 8B,C, upper row zoom-in and blue circle), while others lacked clear mCherry-ATG8a co-labeling (Fig. 8B,C, lower row zoom-in and gray circle).

**Figure EV4:**
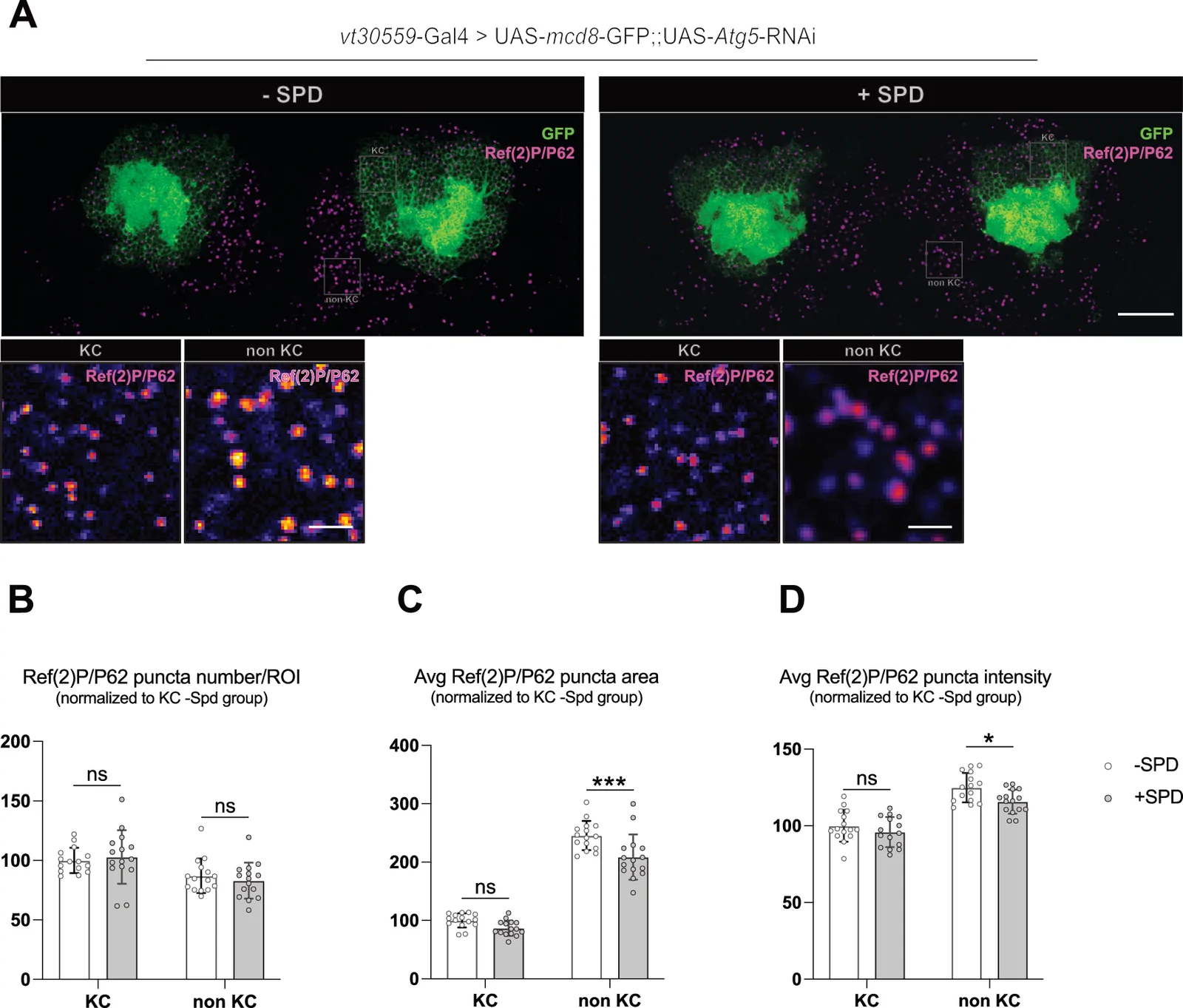
Related to Fig. 8, 5 days spermidine (Spd) supplementation significantly reduces the size and intensity of non-cell autonomous Ref(2)P/P62 aggregates outside the MB KCs. Freshly eclosed *vt30559*-Gal4>UAS-*mcd8*-GFP;;UAS-*Atg5-*RNAi flies were split at day 1 and either transferred onto 5 mM spermidine food or maintained on control food for 5 days. Female fly brains were subsequently dissected and immunostained against Ref(2)P/P62 for confocal microscopic analysis. (**A**) Representative single optical slices of the posterior MB calyx region, exemplifying chosen regions of interest (ROIs) for the analysis of Ref(2)P/P62 puncta in the MB Kenyon cells (KC) and in non-MB neurons (non-KC) of the same optical slice. MB KCs were identified based on the endogenous mcd8-GFP signal driven by *vt30559*-Gal4. Scale bar upper panels = 25 µm; Scale bar lower panels  = 5 µm (**B**–**D**) Quantification of Ref(2)P/P62 puncta number (**B**), area (**C**), and intensity (**D**) for 5-day-old spermidine-treated (+Spd) and non-treated (-Spd) *vt30559*-Gal4>UAS-*mcd8*-GFP;;UAS-*Atg5*-RNAi flies. The average number, area and intensity of Ref(2)P/P62 spots per individual brain was calculated from a total number of 8 ROIs per each brain region (i.e., KC and non-KC), retrieved from two individual optical slices. Measured data from two independent experiments was pooled by normalizing the measured values onto the KC – Spd group. Only relevant statistical comparisons between Spd-supplemented (+Spd) and untreated (−Spd) groups are displayed. The selection of ROIs and suitable optical slices was performed blinded. *n* = 15 adult brains per treatment, pooled from two independent experiments. One-way ANOVA with Tukey’s post hoc test for multiple comparisons. *p = 0.0487, ***p = 0.0009, ns not significant. Data presented as mean ± SD. Source data are available online for this figure.

These findings indicate that autophagy is disrupted cell-autonomously upstream of autophagosome formation in MB KCs, consistent with the requirement of ATG5 for ATG8a lipidation. In contrast, the widespread accumulation of Ref(2)P/P62 aggregates co-labeled with ATG8a in non-targeted neurons suggests that autophagosome formation per se remains functional outside the MB. The widespread accumulation of Ref(2)P/P62 aggregates outside the MB therefore most likely reflects an imbalance between proteostatic load and degradative capacities, rather than a global block of autophagy. The heterogeneous recruitment of ATG8a to these aggregates further suggests regionally distinct compensatory responses to proteostatic stress. Consistent with this interpretation, electron microscopy (EM) revealed protein aggregates within degradative lysosomal compartments in neurons outside the MB (Fig. 8D). While our qualitative EM analysis (Fig. 8D) could not clearly identify double-membraned autophagosomes, despite the evidence of ATG8a-positive Ref(2)P/P62 structures observed with confocal microscopy, it suggests that autophagosome intermediates are either highly transient or rapidly mature into degradative compartments, resulting in predominant detection of late-stage autolysosomal structures outside the MB KCs. Along those lines, pharmacological enhancement of autophagy (Eisenberg et al, 2009; Gupta et al, 2013) by 5 days of spermidine (Spd) supplementation of MB-autophagy impaired flies (Fig. EV4) did not influence the number of Ref(2)P/P62 puncta per chosen ROI (Fig. EV4A,B), but effectively reduced the size (Fig. EV4A,C) and intensity (Fig. EV4A,D) of non-cell autonomous Ref(2)P/P62 puncta, while cell autonomous protein aggregates within the MB KCs were not modulated to similar extends (Fig. EV4). These results underline that autophagic degradation in neurons outside the MB is still functional, but that its degradative capacity is limited and challenged by the cargo load.

Finally, when co-expressing UAS-GFP-*Ref(2)P* together with the UAS-*Atg5*-RNAi specifically within the MB KCs (*vt30559*-Gal4), weaker GFP-Ref(2)P puncta could be detected non-cell autonomously outside the MB in both posterior and anterior brain regions (Fig. 8E), highlighting a spreading of autophagic substrates from the genetically targeted MB KC neurons to other areas of the brains. While the precise molecular mechanisms underlying this cargo relocation remain to be determined, these findings further suggest that the accumulation of non-cell autonomous aggregates likely reflects a condition in which degradative capacity is challenged relative to cargo load, rather than a complete failure of autophagy.

## Discussion

Neural circuits must remain functionally stable while responding flexibly to changing demands, stressors, and aging-related decline (Diniz and Crestani, 2023; Navakkode and Kennedy, 2024). This balance is achieved through plasticity programs that integrate molecular, metabolic, and activity-dependent signals to reconfigure synaptic strength and connectivity (Huang et al, 2022; Parrino and Vaudano, 2018). While many such programs are described at the level of individual synapses or pathways, much less is known about how localized stress responses—such as impairments in proteostasis—can instruct circuit-wide adaptation to promote resilience. Autophagy, as a core mechanism for protein turnover and organelle quality control, is increasingly implicated in this form of adaptive plasticity (Compans et al, 2021; Trujillo-Del Río et al, 2026; Yang et al, 2025).

This study reveals a molecularly resolved, brain-wide synaptic remodeling program triggered by local impairment of autophagy within the *Drosophila* mushroom body (MB). By combining targeted genetic manipulation, proteomic profiling, and circuit-level analysis, we identify a defined presynaptic adaptation characterized by active zone (AZ) scaffold expansion and altered excitability mediated by coordinated changes in calcium and potassium channels. These molecular changes emerge largely through post-transcriptional regulation and occur in the absence of widespread synaptic degeneration. Instead, they correlate with increased sleep, enhanced stress resilience and extended survival—supporting the interpretation that they form part of a resilience-promoting plasticity program.

Previously, we described a non-cell autonomous increase in presynaptic abundance of the AZ scaffold protein BRP in response to MB-specific autophagy impairment (Bhukel et al, 2019), raising the possibility that local disruptions in proteostasis can instruct global synaptic remodeling. Here, we demonstrate that this effect is not limited to BRP, but is embedded in a broader reorganization of presynaptic architecture. Our proteomic analysis revealed upregulation of several core AZ proteins, including RIM, RBP, Liprin-α, Syd-1, Unc13A, and Spinophilin (Spn), along with concurrent downregulation of the AZ VGCC components Cac and stj and upregulation of Shaker-type potassium channels (Figs. 5 and 6A,C). Together, these changes suggest a reconfiguration of release site number and vesicle release probability, consistent with a shift toward lower-gain, energetically efficient transmission (Huang et al, 2022).

This remodeling program was accompanied by a striking non-cell autonomous accumulation of protein aggregates labeled with the autophagy cargo receptor Ref(2)P/P62, both detected by proteomic approaches (Fig. 7A,D) and in spatially resolved immunohistochemical analyses using confocal microscopy (Fig. 7B; Appendix Fig. S7). Notably, Ref(2)P/P62- and ubiquitin-positive aggregates accumulated throughout the brain—even in regions remote from the MB and without direct genetic manipulation—indicating a non-cell autonomous spread of autophagic stress or substrate buildup (Figs. 7 and 8). This observation points to circuit-level communication of proteostatic load, potentially involving partial redistribution of protein aggregates from MB neurons to other brain regions, as supported by the appearance of Ref(2)P-GFP-positive puncta outside the MB upon MB-specific expression (Fig. 8E). Such spreading mechanisms have been described in mammalian systems, where protein aggregates and autophagic intermediates can transfer between neurons or glia (Chakraborty et al, 2023; Fussi et al, 2018; Guo and Lee, 2014; Miranda et al, 2018; Palumbos et al, 2025). Importantly, the widespread Ref(2)P/P62 aggregates in non-targeted brain areas were often associated with mCherry-ATG8a-positive structures (Fig. 8A–C) and could be identified in degradative lysosomal compartments by ultrastructural EM analysis (Fig. 8D), suggesting that autophagic degradation processes remain active in these cells. However, the accumulation of Ref(2)P/P62-positive material indicates an imbalance between proteostatic load and degradative capacity, rather than a complete block in autophagic flux (Fig. 8). In contrast, within MB KCs where *Atg5* was knocked down, autophagy is blocked upstream, before autophagosome maturation, as expected based on ATG5’s essential role in ATG8 lipidation (Fig. 8A; Appendix Fig. S8). This differential pattern of autophagic phenotypes supports the notion that local disruptions in autophagy can trigger a brain-wide, regionally heterogeneous breakdown in proteostatic handling, involving both direct and indirect mechanisms. Notably, recent work has described non-cell autonomous ISR activation accompanied by Ref(2)P/P62-positive stress granule formation (Shekhar et al, 2025). In contrast, although we observed widespread Ref(2)P/P62 accumulation (Fig. 7A–C), we could not find evidence for global ISR activation, as indicated by unchanged eIF2α phosphorylation and ATF4 levels (Fig. EV3), suggesting a mechanistically distinct form of non-cell autonomous proteostatic remodeling. Consistently, molecular signatures of ISR activation were also absent from the global proteomic analysis (Fig. 5).

Despite the widespread proteostatic perturbation, our data show that this remodeling is not associated with overt functional decline. MB-autophagy-impaired animals exhibit increased sleep (Fig. 2A–D), preserved oxidative stress resistance (Fig. EV1A–D), and even display a mild lifespan extension (Figs. 3C–H and 4E,F). At first glance, the coexistence of widespread proteostatic burden with improved organismal outcomes may appear contradictory. However, our data support a model in which moderate, spatially distributed proteostatic stress elicits an adaptive response. Our ultrastructural analyses reveal engagement of degradative compartments in neurons outside the MB (Fig. 8D), indicating active degradative processing rather than failure of proteostasis. In this framework, the observed phenotype is consistent with a hormesis-like response, where controlled stress enhances cellular resilience and organismal fitness (Calabrese et al, 2010; Wiegant et al, 2012). Coherent with this interpretation, these effects align with the previously described PreScale plasticity—a brain-wide, transient, presynaptic upscaling process marked by BRP elevation and linked to sleep need and early aging adaptation (Huang et al, 2020; Huang et al, 2022). Our experiments extend this framework by demonstrating that activation of PreScale-like remodeling can be accelerated via MB autophagy impairment, and conversely, can be delayed by preserving MB autophagy during early aging (Fig. 1). Specifically, enhanced BRP upregulation in MB *Atg5* knockdown animals was already detectable by 5 and 15 days of age (Figs. 1A,B and 4B,C), whereas *Atg5* overexpression suppressed brain-wide BRP increases in a similar time window around day 15 (Fig. 1E). By 30 days of age, differences between genotypes had levelled out (Fig. 1C,F), suggesting that MB-autophagy status controls the timing of PreScale activation but not its final extent, keeping levels of presynaptic AZ proteins in a physiologically relevant range (Fig. 1G).

This bidirectional control of PreScale kinetics positions the MB as a central node linking local metabolic and proteostatic stress to global circuit remodeling. The MB is well established as a hub for sleep regulation in *Drosophila* (Joiner et al, 2006; Pitman et al, 2006), integrating both sleep-promoting and wake-promoting pathways (Sitaraman et al, 2015). Our findings suggest that it also acts as a proteostatic sensor, using its autophagy status to regulate presynaptic output and activity patterns in a manner that promotes restorative sleep and preserves resilience. Notably, similar manipulations in other neuronal populations did not elicit comparable brain-wide responses (Appendix Fig. S1), suggesting a specialized role of the MB in coordinating this form of plasticity.

The causal link between synaptic remodeling and behavioral resilience is further supported by our genetic manipulation of PreScale levels in flies with MB-specific autophagy impairment. Decreasing *brp* gene dosage was sufficient to abolish the increase in survival observed in MB *Atg5* knockdown animals (Fig. 3I,J), underlining a functional role for BRP-driven AZ expansion in mediating these protective adaptive outcomes. These findings are in line with prior studies showing that BRP upscaling in PreScale is necessary for sleep homeostasis following deprivation (Huang et al, 2020; Weiss et al, 2024; Weiss and Donlea, 2021) and sufficient to mediate sleep pattern changes in aging animals (Huang et al, 2022).

At the mechanistic level, our data suggests that MB autophagy status influences synaptic remodeling largely through post-transcriptional means. Despite pronounced changes in protein levels across AZ components and excitability regulators, corresponding transcript levels remained largely unchanged (Appendix Fig. S6). This pattern points to regulated translation, protein turnover, or compartment-specific adjustments of proteostasis as key mechanisms for modulating synaptic protein composition. In this regard, the observed enrichment of ER-associated proteins and chaperones in our proteomic data is noteworthy (Figs. 5A,C,E and 6D). Although not the dominant feature of the remodeling program, ER remodeling and the unfolded protein response may support translational buffering and may contribute to the control of neurotransmission-relevant Ca^2+^ homeostasis at presynaptic compartments, as described in mammalian neurons (Kuijpers et al, 2021). In addition, the induction of chaperones and small heatshock proteins may further support handling of proteostatic stress under these conditions and contribute to the observed pro-longevity effects, likely acting in parallel with PreScale-driven synaptic remodeling as part of a coordinated adaptive response (Liao et al, 2008).

Our findings also resonate with broader concepts in neurodegeneration and resilience. In mammalian systems, resilience to Alzheimer’s pathology has been linked to the preservation of specific synaptic protein modules (Hurst et al, 2023), and autophagic flux is a key determinant of neuronal survival in both aging and disease (López-Otín et al, 2023; Nixon, 2013; Nixon and Rubinsztein, 2024). The MB, as a central integrator of synaptic, metabolic, and proteostatic information (Suárez-Grimalt et al, 2024), may thus serve as a model for understanding how localized stress responses can be translated into brain-wide adaptive reprogramming.

In sum, we define a genetically tractable, mechanistically resolved program of presynaptic plasticity that links autophagy status in the MB to circuit-wide remodeling, sleep behavior, and stress resilience. This program shares key features with PreScale, including BRP-centered AZ expansion and behavioral restoration, but our data extend the model by identifying upstream triggers, molecular logic, and bidirectional regulatory control. More broadly, our work highlights the ability of local proteostatic stress to shape presynaptic architecture and function across the brain, establishing a framework for studying resilience as an emergent property of distributed synaptic plasticity.

The identification of a non-cell autonomous synaptic resilience program centered on presynaptic remodeling in response to autophagy impairment has important implications beyond basic neurobiology. Notably, many neurodegenerative disorders are associated with mutations in genes involved in proteostasis and autophagy, including p62/SQSTM1 as well as VCP, UBQLN2, OPTN, and C9ORF72—several of which are implicated in amyotrophic lateral sclerosis (ALS) and frontotemporal dementia (FTD), and emerge as key factors of stress and aging (Beckers and Van Damme, 2025; Cozzi and Ferrari, 2022; Fecto et al, 2011; Kumar et al, 2022; Nixon and Rubinsztein, 2024). The demonstration that local autophagy impairment can systemically reprogram synaptic organization and function suggests new paths for understanding how focal cellular dysfunction spreads through neural networks. In particular, our work supports a model in which AZ-remodeling acts as a compensatory adaptation to local autophagic stress, one that may initially preserve function but later become maladaptive. Elucidating this balance between resilience and pathological remodeling could therefore provide new therapeutic opportunities to enhance beneficial aspects of synaptic plasticity while curbing their long-term destabilizing effects.

## Methods

**Table:** Reagents and tools table

Reagent/resource	Reference or source	Identifier or catalog number
**Experimental models**		
*w*^*1118*^ (Wildtype) (*Drosophila melanogaster*)	Bloomington *Drosophila* Stock Center	#5905
*Atg7*^*d77*^ (*Drosophila melanogaster*)	Juhász et al, (2007)	N/A
*sss*^*P1*^ (*Drosophila melanogaster*)	Bloomington *Drosophila* Stock Center	#16588
1x BRP (*brp*^*c04298*^*)* (*Drosophila melanogaster*)	Bloomington *Drosophila* Stock Center	#85966
3xmCherry-*Atg8a* (*Drosophila melanogaster*)	Hegedűs et al, (2016)	N/A
*me31b*-GFP (*Drosophila melanogaster*)	Formicola et al, (2021)	N/A
*elav*-Gal4 (*Drosophila melanogaster*)	Bloomington *Drosophila* Stock Center	#458
*vt30559*-Gal4 (*Drosophila melanogaster*)	Vienna *Drosophila* Resource Center	#206077
*ok107*-Gal4 (*Drosophila melanogaster*)	Aso et al, (2009)	N/A
*R24B11*-Gal4 (*Drosophila melanogaster*)	Bloomington *Drosophila* Stock Center	#49070
*R58H05*-Gal4 (*Drosophila melanogaster*)	Bloomington *Drosophila* Stock Center	#39198
*R23E10*-Gal4 (*Drosophila melanogaster*)	Bloomington *Drosophila* Stock Center	#49032
*R27E08*-Gal4 (*Drosophila melanogaster*)	Bloomington *Drosophila* Stock Center	#49226
AGES Gal80 (*Drosophila melanogaster*)	Bloomington *Drosophila* Stock Center	#92470
UAS-*Atg5*-RNAi (*Drosophila melanogaster*)	Bloomington *Drosophila* Stock Center	#34899
UAS-GFP-*Atg5* (*Drosophila melanogaster*)	Bloomington *Drosophila* Stock Center	#59848
UAS-*Atg7* (*Drosophila melanogaster*)	Chen et al (2012)	N/A
UAS-*mcd8*-GFP (Fig. 7) (*Drosophila melanogaster*)	Sigrist Laboratory	Stock #15
10xUAS-*mcd8*-GFP (Fig. 4) (*Drosophila melanogaster*)	Bloomington *Drosophila* Stock Center	#32184
UAS-*mcd8*-RFP (*Drosophila melanogaster*)	Bloomington *Drosophila* Stock Center	#32219
UAS-GFP-*Ref(2)P* (*Drosophila melanogaster*)	Bloomington *Drosophila* Stock Center	#605356
UAS-*Cas9* (*Drosophila melanogaster*)	Bloomington *Drosophila* Stock Center	isolated from #67073
*Atg5*-gRNA (*Drosophila melanogaster*)	Bloomington *Drosophila* Stock Center	#80819
BRP-170-GFP (*Drosophila melanogaster*)	Sigrist Laboratory	Stock #2219
BRP FlySam2.0 (*Drosophila melanogaster*)	This study/ Sigrist Laboratory	Stock #402
**Recombinant DNA**		
N/A	N/A	N/A
**Antibodies**		
anti-BRP^Nc82^	DSHB	AB_2314866
anti-BRP^D2^	Sigrist Laboratory Chen et al (2012)	rb5228
anti-Spn^2.2^	Sigrist Laboratory Muhammad et al (2015)	N/A
anti-Ref(2)P/P62	Abcam	Ab178440
anti-Ubiquitin (UBCJ2)	Enzo Life Sciences	ENZ-ABS840-0100
anti-GFP (IHC)	Abcam	Ab13970
anti-GFP (WB)	Sigma-Aldrich	SAB4301795
Anti-p-eIF2α Ser51	Cell Signaling Technology	#3597 L
Anti-RFP	Chromotek	5F8
anti-ATF4	Proteintech Group Inc	60035-1-Ig
anti-ATG8a	Abcam	Ab109364
anti-Tubulin	Sigma-Aldrich	T5168_2ML
anti-Mouse Alexa Fluor 488	Invitrogen	Cat# A-11029
anti-Chicken Alexa Fluor 488	Invitrogen	Cat# A-11039
anti-Rabbit Alexa Fluor 488	Invitrogen	Cat# A-11070
anti-Mouse Cy3	Abcam	Cat# ab97035
anti-Rabbit Cy5	Invitrogen	Cat# A10523
Anti-Rat Cy5	Invitrogen	Cat# A10525
anti-Mouse POD	Biozol	Cat#115-035-003
anti-Rabbit POD	Biozol	Cat# 111-035-144
**Oligonucleotides and other sequence-based reagents**		
PWG6020 FlySAM2.0 vector	Jia et al, (2018)	N/A
**Chemicals, enzymes and other reagents**		
Spermidine	Sigma-Aldrich	Cat#S2626
Naphthaleneacetic Acid (Auxin)	PhytoTechlabs	ID N610
cOmpleteTM Mini protease inhibitor cocktail	Roche	#11836153001
**Software**		
Sleep and Circadian Analysis MATLAB Program (SCAMP)	Vecsey et al, (2024)	N/A
MaxQuant v.1.6.2.6	MaxQuant	N/A
Perseus v.1.6.2.3.	MaxQuant	
Metascape	Zhou et al, (2019)	N/A
Cytoscape v.3.8.2	https://cytoscape.org/ Shannon et al (2003)	N/A
Fiji/Image J v.153q	Schindelin et al, (2012)	N/A
iTEM software	Olympus	N/A
GrapPad Prism 8	GraphPad Software Inc.	N/A
Affinity Design 2.5.6	Affinity.studio	N/A
Excel	Microsoft	N/A
**Other**		
Drosophila activity monitors (DAM2)	Trikinetics Inc.	N/A
FlyPad	Itskov et al, (2014)	N/A
Dionex UltiMate 3000 system	Thermo Fisher Scientific	N/A
Orbitrap Fusion Lumos Instrument control software 3.1	Thermo Fisher Scientific	N/A
Leica TCS SP8 inverted confocal microscope	Leica Microsystems GmbH	N/A
JEM-1011 transmission electron microscope	Jeol	N/A

### Drosophila stocks and maintenance

Fly stocks and experimental crosses were maintained under standard laboratory conditions on semi-defined food medium (Bloomington recipe) under a 12/12 h light/dark cycle with 65% humidity at 25 °C. Crosses and progeny for proteomics and RNA-Sequencing experiments (*ok107*-Gal4/+ and ok*107*-Gal4>UAS-*Atg5*-RNAi) were raised and maintained at 29 °C (Bhukel et al, 2019). All flies were backcrossed to the same *w*^*1118*^ (*iso31*, BDSC #5905) background for at least six generations, except for 3xmCherry-*Atg8a* flies. If not otherwise indicated, 5-day-old flies were used for all experiments.

### Spermidine and auxin supplementation

Spermidine (Sigma-Aldrich, Cat#S2626) and naphthaleneacetic Acid (Auxin; PhytoTechlabs ID N610) stock solutions were prepared with double-distilled H_2_O and added accordingly to freshly cooked fly food medium in order to reach a final concentration of 5 mM for spermidine and of 10 mM for auxin. Control food without a supplement was prepared from the same batch by adding an equal volume of H_2_O. For spermidine, the fly food was cooled down to 40 °C before the addition of the supplement. For spermidine experiments (Fig. EV4), newly eclosed flies were transferred for 5 days onto the respective food before being dissected for immunohistochemical analysis.

### Sleep measurements

Sleep and locomotor activity measurements of single adult flies was performed with *Drosophila* activity monitors (DAM2) from Trikinetics Inc. (Waltham, MA., USA) at 25 °C and 65% humidity in a 12/12 h light/dark cycle (Huang et al, 2020; Pfeiffenberger et al, 2010). At day 3 of age, individual flies were loaded under brief CO_2_ anesthesia into single Trikinetics glass tubes (5 mm inner diameter and 65 mm length) containing a food source of 5% sucrose and 2% agar on one side. The activity of individual flies was recorded as the number of beam crosses within 1 min, and periods of inactivity without any beam cross of 5 min or longer were considered as sleep (Shaw et al, 2000). Activity measurements were performed over 5 consecutive days, and measurements from the first 2 days were discarded due to entrainment of the flies to the new environment. Data from the last 3 days was averaged, and sleep parameters were analyzed using the Sleep and Circadian Analysis MATLAB Program (SCAMP) (Vecsey et al, 2024). Activity measurements of flies which died during the experiment were discarded from the analysis. For sleep measurements of aged flies, 13-day-old flies were loaded into Trikinetics glass tubes so that they reached 15 days of age at the start of the relevant recording period.

### Longevity assay

Newly eclosed flies were collected every other day and maintained in groups of mixed sex for 2 days. At day 3, male and female flies were quickly separated under CO_2_ anesthesia and sorted into populations of ~25 individuals per vial. This procedure was repeated on different days with different cohorts of collected flies, until 6-10 experimental vials per sex and genotype were set up. The established longevity cohorts were transferred onto fresh food every second day, and the number of individual dead flies per vial was recorded at each time point of transfer.

### H_2_O_2_ survival assay

Freshly eclosed flies were collected every other day and maintained in groups of mixed sexes for 2 days. Subsequently, 45 flies per genotype and sex were transferred onto big vials containing three filter papers soaked with 2 ml of solution consisting of 2% H_2_O_2_ and 5% sucrose diluted in water. Experimental cohorts were transferred onto new vials with freshly prepared H_2_O_2_ solutions every second day, and the number of individual death events in each vial was scored exactly at the same time of the day every 24 h.

### Feeding assay

Feeding assays were performed using FlyPad as previously described (Itskov et al, 2014). 3-day-old female flies were sorted in groups of 25 under brief CO₂ anesthesia and housed in vials containing standard cornmeal food. Two days later, individual flies were transferred by mouth aspiration into FlyPad arenas and allowed to feed for 1 h on two 5 μl drops of 1% agar (Karl Roth, 4508.1) supplemented with 5% sucrose (Sigma-Aldrich, S5016) and 5% yeast extract (Karl Roth, 2363.3). Experiments were conducted in the dark at 25 °C and 70% relative humidity. For H₂O₂ feeding experiments, groups of female flies were transferred 1 day prior to the experiment to vials containing filter paper soaked with 2% H₂O₂ and 5% sucrose diluted in tap water. During the feeding assay, flies were allowed to feed on food containing 2% H₂O₂, 1% agar, 5% sucrose, and 5% yeast extract. The total number of sips for each fly was calculated using a custom-made MATLAB script provided by the manufacturer. Data from flies showing signs of technical malfunction (rare) or zero sips on one of the electrodes were excluded from further analysis (Musso et al, 2019).

### Western blot

Western blot was performed as previously described (Liang et al, 2021) with minor modifications. In brief, four female adult brains per genotype were dissected in cold hemolymph-like saline solution (HL3 70 mM NaCl, 5 mM KCl, 10 mM NaHCO_3_, 20 mM MgCl_2_, 1.5 mM CaCl_2_, 5 mM HEPES, 5 mM Trehalose, 115 mM Sucrose, and pH =  7.2) and stored in 50 µl HL3 in low-binding Eppendorf tubes on ice. Dissected brains were immediately centrifuged for 5 min at 7000 rpm at 4 °C, the HL3 supernatant was discarded, and the sample was frozen at −20 °C. After completion of sample collection, 30 µl of Lysis buffer (1x PBS, 0.5% Triton-X, 2% SDS, 1x Sample Buffer, 1x Roche cOmplete™ Mini protease inhibitor cocktail (#11836153001) was added onto the brain pellets, and samples were homogenized by mechanical rotation for 1 h at 4 °C, followed by 5 min full-speed centrifugation at 13.000 rpm at room temperature. All genotypes/groups of an experiment were dissected on the same day, and sample collection for Western blotting was always performed in the middle of the day (around ZT5, i.e., 2 pm). Lysed brain samples were boiled for 5 min at 95 °C and subsequently centrifuged for 1 min at 13,000 rpm. An amount equal to two brains for each experimental condition was loaded onto SDS-PAGE gels and immunoblotted according to standard protocols. Blots were manually developed with ECL solution and Kodak/GE films, and subsequently digitalized. The following primary antibody dilutions were used: Rabbit anti-BRP^D2^ (rb5228 (Fouquet et al, 2009), 1:40.000), rabbit anti-Ref(2)P (Abcam Ab178440, 1:1000), rabbit anti-ATG8 (Abcam Ab109364, 1:500), rabbit anti-GFP (Sigma SAB4301795, 1:5000), rabbit anti-p-eIF2α Ser51 (Cell Signaling Technology #3597 L, 1:1000), mouse anti-Tubulin (Sigma T5168, 1:100,000). Secondary horseradish peroxidase (HRP)-conjugated goat anti-mouse and goat anti-rabbit antibodies were used for detecting the protein signals by chemiluminescence (Biozol, 1:5000).

### Sample preparation for label-free global proteomics of adult *Drosophila* brain homogenates

10 female brains per genotype were hand-dissected in cold HL3 and stored in 50 µl HL3 in low-binding Eppendorf tubes on ice, followed by centrifugation at 4 °C and 7000 rpm for 5 min, before being immediately frozen at −80 °C until completion of sample collection. Dissection was always performed in the middle of the day (around ZT5, i.e., 2 pm), and the four biological replicates were dissected on different days from independently collected fly cohorts arising from the same parental cross. About 20 µl of lysis buffer was added onto each frozen brain pellet, and samples were lysed for 1 h at 4 °C on a rotator, followed by 5 min full-speed centrifugation at 13,000 rpm. Subsequently, 1 μl of 100 mM Dithiothreitol (DTT) dissolved in 50 mM triethylammonium-bicarbonate (TEAB) buffer was added to the samples to reach a final concentration of 5 mM of DTT in the solution, and samples were incubated shaking at 300 rpm for 30 min at 55 °C. Protein alkylation was performed by the addition of 2.1 μl of 400 mM chloracetamatide (CAA) dissolved in 50 mM TEAB, to reach a final CAA concentration of 40 mM inside the sample. Samples were incubated shaking at 300 rpm for 30 min at room temperature and in darkness, as CAA is unstable under light exposure. After alkylation, proteomic samples were boiled for 5 min at 95 °C, followed by 1 min of centrifugation at 13.000 rpm. Exactly 20 μl of each sample was loaded onto 4–20% Mini-PROTEAN® TGX™ precast gels (Bio-Rad Laboratories, Hercules, USA), and SDS-PAGE was performed for 2–3 min at 200 V until all proteins migrated out of the loading pockets into the gel. After Coomassie staining of the gel, each lane of the protein sample was cut with sterile scalpels into 4 equal pieces. Gel pieces were transferred into low-binding Eppendorf tubes containing 50 µl of distilled H_2_O, and stored at 4 °C until further processing.

### Liquid chromatography mass spectrometry (MS) analysis

In-Gel protein digestion was performed overnight at 37 °C with trypsin at an enzyme to protein weight ratio of 1:20. Equal volumes of 1 μg of peptide were loaded on a Thermo Scientific Dionex UltiMate 3000 system connected to a PepMap C18 trap-column (0.075 × 50 mm, 3-μm particle size, 100 Å pore size; Thermo Fisher Scientific), followed by an in-house-packed C18 column (column material: Poroshell 120 ECC18, 2.7 μm; Agilent Technologies). After loading, peptides were separated at a 250 nL/min flow rate over a 120 min gradient of increasing acetonitrile concentration and sprayed into an Orbitrap Fusion Lumos (instrument control software 3.1). The MS1 scans were performed in the Orbitrap. Only precursors with a charge state of 2–4 were subjected to fragmentation and then dynamically excluded for 60 s. The MS2 scans were acquired in the ion trap with the following settings: scan rate rapid, 35 ms max. injection time, first mass 110 m/z, isolation window 1.6 m/z, 30% NCE, AGC target 10,000. A 1 s cycle time was set between master scans.

### Proteomic data analysis

#### Database search, data processing, and statistical analysis

Raw data were searched using MaxQuant version 1.6.2.6 in default settings. The number of missed tryptic cleavages allowed was set to 2, “label-free quantification” was enabled, and the “match between runs” option was disabled. The search was performed using the UniprotKB database of *Drosophila melanogaster* downloaded in May 2020 and containing 42,678 entries. Peptide-spectrum match (PSM) and protein false-discovery rate (FDR) were set to 1%. Further data analysis was performed with Perseus (v1.6.2.3). Label-free quantification (LFQ) values were log_2_-transformed to achieve a normal data distribution. Proteins identified in at least three (out of four) biological replicates were considered for further statistical quantification. Missing data values were imputed by values from a normal distribution (width 0.3 standard deviations; down shift 1.8). For statistical protein enrichment analysis, the two experimental groups (i.e., o*k107*-Gal4>UAS-*Atg5*-RNAi and *ok107*-Gal4/+) were compared by an unpaired two-tailed *t*-test, and the −log_10_ *p* value cutoff was set to 1.75. Fold changes were calculated as the difference of mean log_2_-transformed intensity values between *ok107*-Gal4>UAS-*Atg5*-RNAi effect and the *ok107*-Gal4/+ control group.

#### Gene Ontology (GO) functional enrichment analysis

GO functional enrichment analysis was performed with Metascape (Zhou et al, 2019). All identified significantly up- or downregulated proteins were uploaded to the Metascape website (https://metascape.org/), and functional enrichment analysis was performed separately for GO “Biological processes” and GO “cellular components” with a *p* value cutoff of *p* < 0.01, an enrichment factor >1.5, and a number of hits ≥2.

#### Protein–protein interaction (PPI) network analysis

PPI network analysis was performed with Cytoscape (https://cytoscape.org/, Version 3.8.2). Log_2_ fold change (FC) and −log_10_ *p* values of all significantly changed proteins were loaded into the software, and a full string network with a minimum confidence score of 0.4 and 0 additional interactors was created. The size of individual nodes was defined relative to the corresponding −log_10_ *p* value, and the color intensity of individual nodes indicates the size of the log_2_ FC. The size and intensity of the strings between two nodes indicate the confidence score for a given interaction.

### RNA-sequencing

Sample collection for RNA-sequencing was performed as follows: Female flies of the desired age and genotypes were snap-frozen in liquid nitrogen and immediately stored at −80 °C. Subsequently, frozen flies were decapitated with metal sieves without interrupting the cooling chain, and fly heads were immediately transferred into low-binding Eppendorf tubes to be stored at −80 °C. In total, four biological replicates per genotype with ~50 heads/replicate were collected, and QuantSeq 3’ mRNA sequencing was subsequently performed by Lexogen® (Vienna, Austria). Differential expression analysis was provided by the company and was performed with the DESeq2 Bioconductor implemented in R. Significantly changed gene expression between *ok107*-Gal4>UAS-*Atg5-*RNAi effect and *ok107*-Gal4/+ control group was defined by an adjusted *p* value <0.10.

### Immunohistochemistry of adult *Drosophila* brains

Immunohistochemical stainings of adult *Drosophila* brains was performed as previously described (Bhukel et al, 2019) with minor modifications. In brief, flies with the desired age, sex, and genotype were anesthetized on ice, brains were hand-dissected in 50 μl HL3 and stored in glass staining chambers containing 500 μl of HL3 on ice. Brains were immediately fixed in 4% PFA for 40 min at room temperature on a horizontal shaker. After fixation, brains were quickly rinsed two times, followed by three times of 10 min washing in 0.7% PBS-T. Subsequently, brains were incubated for 2 h at room temperature, shaking in 0.7% PBS-T containing 10% Roti® ImmunoBlock (Carl Roth GmbH). Brains were incubated, gently shaking for 48 h at 4 °C in 200 µl of solution containing primary antibodies diluted in 0.7% PBS-T with 5% Roti® ImmunoBlock. In the following, brains were washed in 30 min intervals at least eight times with 0.7% PBS-T, and then incubated with corresponding fluorophore-conjugated secondary antibodies diluted in 0.7% PBS-T containing 5% Roti® ImmunoBlock overnight at 4 °C on a shaker. Brains were washed in 30 min intervals at least three times with 0.7% PBS-T, and subsequently mounted on glass slides using Vectashield® Mounting Medium (Vector Laboratories Inc.). Samples were covered with high-precision coverslips No. 1.5H (Carl Roth GmbH) and sealed with nail polish. Mounted brains were stored at −20 °C in darkness until the day of microscopy image acquisition to preserve the fluorescence.

The following primary antibody dilutions were used: Mouse anti-BRP^Nc82^ (DSHB AB_2314866; 1:50 for adult brain, 1:250 for NMJ), rabbit anti-Spn^2.2^ (Muhammad et al, 2015; 1:1000), rabbit anti-Ref(2)P (Abcam Ab178440; 1:1000), mouse anti-Ubiquitin (Enzo Life Sciences ENZ-ABS840-0100; 1:250), chicken anti-GFP (Abcam Ab13970; 1:1500), rat anti-RFP (Chromotek 5F8; 1:250), mouse anti-ATF4 (Proteintech Group Inc 60035-1-Ig; 1:500). Corresponding fluorophore-coupled secondary antibodies were used at a dilution of 1:400.

### Confocal image acquisition

Confocal images of immunofluorescently stained adult *Drosophila* brains were acquired with a Leica TCS SP8 inverted confocal microscope (Leica Microsystems GmbH) at the BioSupraMol Optical Microscopy Facility of the Freie Universität Berlin, Germany. Optical slices of the whole brain were acquired at a format of 1024 × 1024 pixels and 0.75x zoom with a 40x, 1.3 NA (numerical aperture) oil immersion objective. Image stacks were acquired in line scanning mode at a speed of 400 Hz and at a Z-step size of 1 μm. For higher magnifications, selected brain areas were scanned with a 63x, 1.4 NA oil immersion objective and variable zoom and Z-step size settings, while all other parameters remained the same as for whole brain image acquisition. All settings were kept exactly the same for the image acquisition of all groups/conditions of a given experiment.

### Confocal image processing and analysis

Image, processing, analysis and the selection of representative stack projections was performed with Fiji/Image J software (Version 1.53q; (Schindelin et al, 2012)).

### Whole-brain mean fluorescence intensity measurement

Measurements of whole brain mean fluorescence intensities was performed on average projections of confocal image stacks, comprising all optical slices between the beginning of the antennal lobes at the anterior side and the end of the dorsal fan-shaped body brain structure towards the posterior side. A region of interest (ROI) was manually drawn around the whole brain, and the mean gray value inside the ROI was measured. Individual intensities of all groups of a given experiment were normalized to the mean intensity of the control group in order to obtain the relative difference between the control and experimental groups. For whole-brain BRP intensity measurements, each experiment was independently performed twice, and the normalized intensity values obtained for each experiment were pooled together for analysis. For analysis of 3xmCherry-ATG8a intensities inside the MB lobes, a ROI was manually drawn around the MB lobes for single optical slices at an interval of four slices. Subsequently, missing ROIs were interpolated and manually corrected if needed, and 3xmCherry-ATG8a intensity was measured for each ROI per individual optical slice to obtain an average intensity value over the whole MB neuropile. Measured values were subsequently normalized to the average value of the control group.

### Ref(2)P/P62 and 3xmCherry-ATG8a puncta analysis

Quantification of Ref(2)P/P62 puncta number, size and intensity was performed on single optical slices. Images were thresholded and ROIs for individual Ref(2)P/P62 puncta were obtained with the “Find Maxima” and “Analyze particles” functions in FIJI/ ImageJ. The same ROIs obtained from Ref(2)P/P62 puncta were applied to the 3xmCherry-ATG8a channel, in order to obtain the corresponding 3xmCherry-ATG8a intensity for individual Ref(2)P/P62 puncta.

### Ref(2)P/P62 puncta analysis of spermidine-supplemented animals

Quantification of Ref(2)P/P62 puncta number, size and intensity was performed on single optical slices. Images were thresholded and ROIs for individual Ref(2)P/P62 puncta were obtained with the “Find Maxima” and “Analyze particles” functions in FIJI/ImageJ. For each brain, two posterior optical slices in the MB calyx region were chosen based on the mcd8-GFP signal, and four ROIs per optical slice were placed within the GFP-positive MB KCs, as well as four ROIs in non-KC cell body regions of the same optical slice, respectively. Measured values were pooled in order to estimate the average Ref(2)P/P62 puncta number, size, and intensity per individual brain. The analysis was performed blindly.

### Transmission electron microscopy

Dissected adult brains of 5-day-old *vt30559*-Gal4>UAS-*Atg5*-RNAi flies were fixed in 3.2% formaldehyde, 1% glutaraldehyde, 1% sucrose, and 0.028% CaCl_2_ in 0.1 N sodium cacodylate, pH 7.4, overnight at 4 °C. Samples were then postfixed in 0.5% osmium tetroxide for 1 h and in half-saturated aqueous uranyl acetate for 30 min at RT, dehydrated in a graded series of ethanol, and embedded in Durcupan (Fluka) according to the manufacturer’s recommendations. About 70-nm sections were stained in Reynolds lead citrate and viewed on a JEM-1011 transmission electron microscope (Jeol) equipped with a Morada digital camera (Olympus) using iTEM software (Olympus).

### Generation of transgenic BRP FlySam2.0 animals

The generation of the BRP FlySam2.0 fly line followed the guidelines of the CRISPR/Cas9 transcriptional activation (CRISPRa)/FlySAM approach (Jia et al, 2018) and was performed by WellGenetics Inc. (Taipei City, Taiwan). The gRNA design was based on parameters optimized for the FlySAM method (Mao et al, 2020). The gRNA sequence TCGCGCAGTTCAGTGAGTGC[TGG] was cloned into the PWG6020 FlySAM2.0 vector using BbsI cutting sites. The recombinant FlySAM2.0 vector, containing a UAS-*dcas9-vp64* construct and the U6:2-controlled gRNA targeting the [TGG] PAM sequence 186 bp upstream of the *brp* transcription start site, was microinjected into [LWG126] y[1] M{vas-int.Dm}ZH-2A w[*]; P{y[+t7.7]=CaryP}attP40 animals according to standard protocols and integrated into the genome by Phi-C31-mediated transgenesis. The screening for transformants was performed using the visible *vermilion* marker. BRP FlySam2.0 animals were isogenized into the *w*^*1118*^ (*iso31*, BDSC #5905) background for six generations prior to longevity experiments.

### Statistical analysis and visual data presentation

Data sets were analyzed and plotted into representative graphs using GraphPad Prism 8 (GraphPad Software Inc.). If not stated otherwise, data from two independent experiments were pooled together by normalizing all measured values of an experiment to the respective control group/condition. Data sets were tested for normality, and statistical comparisons between two experimental groups was performed by unpaired two-tailed *t*-test (normal distribution) or by Mann–Whitney test (skewed distribution). Statistical testing between more than two experimental groups was achieved by one-way analysis of variance (ANOVA) followed by Tukey’s post hoc test for multiple comparisons. Kaplan–Meier survival curves were statistically compared pairwise by the log-rank (Mantel–Cox) Test. The linear regression model for Fig. 8C was calculated in Excel (Microsoft). Statistical significance is indicated as follows: *p < 0.05; **p < 0.01; ***p < 0.001; ****p < 0.0001; ns not significant = *p* > 0.05. Exact *p* values are reported in the figure legends to four digits after the decimal point. All figures were created with Affinity Design 2.5.6.

## Supplementary information


Appendix
Peer Review File
Source data Fig. 1
Source data Fig. 2
Source data Fig. 3
Source data Fig. 4
Source data Fig. 5
Source data Fig. 6
Source data Fig. 7
Source data Fig. 8
EV Figure Source Data
Expanded View Figures


## Data Availability

The data and resources that support the findings of this study are available from the corresponding author upon reasonable request. Source data underlying the main and EV figures are uploaded alongside this article. RAW RNA-sequencing data were uploaded to the ArrayExpress database under the accession number E-MTAB-17037 (https://www.ebi.ac.uk/biostudies/arrayexpress/studies/E-MTAB-17037). RAW proteomics data were uploaded to the PRIDE database under the project accession number PXD076163 (https://www.ebi.ac.uk/pride/archive/projects/PXD076163/). The source data of this paper are collected in the following database record: biostudies:S-SCDT-10_1038-S44318-026-00847-4.
