# Supplementary material for: Local autophagy impairment triggers brain-wide presynaptic remodeling and resilience
Source: EMBO J. 2026 Jul 8;45(17):6054–85. doi: 10.1038/s44318-026-00847-4 (PMC13534471; doi:10.1038/s44318-026-00847-4)
Supplement: Supplementary file 1 [file 44318_2026_847_MOESM12_ESM.pdf]

Figure EV1. Related to Fig. 3, MB-specific *Atg5* knockdown does not attenuate H_2O_2 resistance or feeding behavior.

(**A**, **B**) Kaplan–Meier survival curves of male (**A**) and female (**B**) flies with MB-specific *Atg5* knockdown (*ok107*-Gal4>UAS-*Atg5*-RNAi, blue curve) compared to *ok107*-Gal4/+ and UAS-*Atg5*-RNAi/+ control flies continuously exposed to 2% H_2O_2 from day 2 onwards. $n = 85–89$ flies per genotype and sex. Pairwise log-rank (Mantel–Cox) test comparison. *$p = 0.0180$, **$p = 0.0011$, ns not significant. (**C**, **D**) Kaplan–Meier survival curves of male (**C**) and female (**D**) flies with MB-specific *Atg5* knockdown (*vt30559*-Gal4>UAS-*Atg5*-RNAi, green curve) compared to *vt30559*-Gal4/+ and UAS-*Atg5*-RNAi/+ control flies continuously exposed to 2% H_2O_2 from day 2 onwards. $n = 87–90$ flies per genotype and sex. The UAS-*Atg5*-RNAi/+ controls in (**A**, **B**) are the same as in (**C**, **D**). Pairwise log-rank (Mantel–Cox) test comparison. ***$p = 0.0003$, ****$p < 0.0001$, ns not significant. (**E**) Number of sips/hour of 5-day-old female *vt30559*-Gal4>UAS-*Atg5*-RNAi and *ok107*-Gal4>UAS-*Atg5*-RNAi flies compared to their respective age- and sex-matched controls (*vt30559*-Gal4/+ and *ok107*-Gal4/+). Flies were sorted at 3 days of age and transferred 24 h before the experiment onto 2% H_2O_2 containing sucrose solution. During the experiment, consumption of 2% H_2O_2-containing food solution was automatically assessed in FlyPad arenas. $n = 25–30$ flies per genotype pooled from two independent experiments. Mann–Whitney test. ns not significant. (**F**) Number of sips/hour of 5-day-old female *vt30559*-Gal4>UAS-*Atg5*-RNAi and *ok107*-Gal4>UAS-*Atg5*-RNAi flies compared to their respective age- and sex-matched controls (*vt30559*-Gal4/+ and *ok107*-Gal4/+). Flies were sorted at 3 days of age and the consumption of food solution containing 1% Agar, 5% yeast and 5% sucrose was automatically assessed in FlyPad arenas. $n = 29–45$ flies per genotype pooled from three independent experiments. Mann–Whitney test. *$p = 0.0270$, ns not significant. Data presented as mean ± SD. Source data are available online for this figure.

© The Author(s)

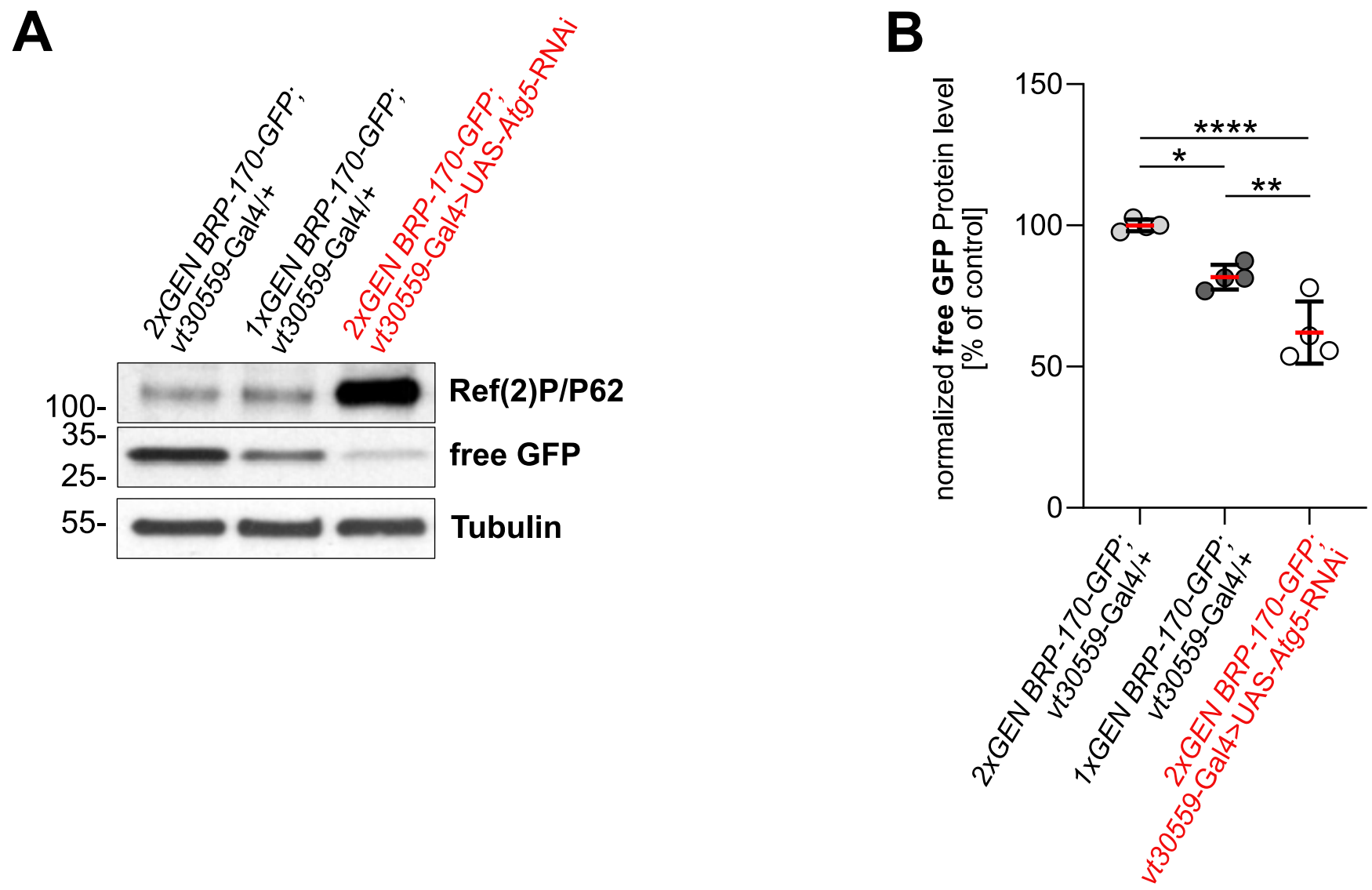

Figure EV2. Related to Fig. 6, lysosomal degradation of a GFP-tagged 170 kDa BRP fusion protein is strongly reduced in fly brains with MB-autophagy impairment.

(A) Representative Western blot of brain homogenates from 5-day-old female flies expressing either one or two additional genomic copies of a GFP-tagged BRP 170 kDa isoform. Control flies additionally carry the *vt30559*-Gal4/+ transgene alone, while in the effect group, UAS-*Atg5*-RNAi is driven exclusively in the MB under control of *vt30559*-Gal4. Membranes were probed against GFP and Ref(2)P/P62, and Tubulin was used as a loading control. An amount equal to two brains was loaded for each lane. **(B)** Quantification of free GFP protein amounts. Measured band intensities were first normalized to the respective Tubulin band intensity, and values of all groups were normalized to the 2xGen BRP-170-GFP;*vt30559*-Gal4/+ control group. $n = 4$ independently prepared Western blot samples run on two separate gels. One-way ANOVA with Tukey's post hoc test for multiple comparisons. *$p = 0.0120$, **$p = 0.0079$, ****$p < 0.0001$. Data presented as mean ± SD. Source data are available online for this figure.

© The Author(s)

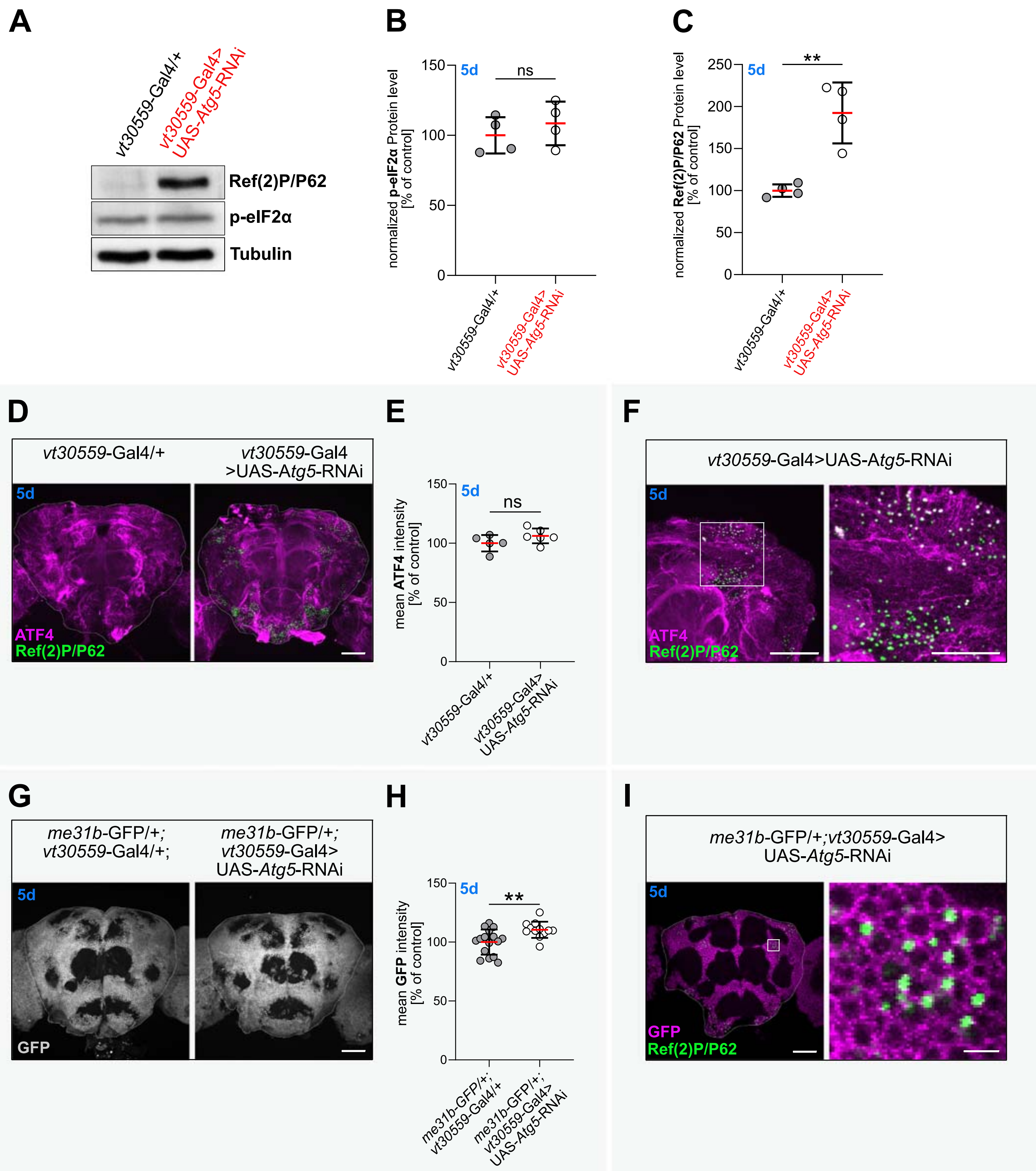
A
vt30559-Gal4/+
vt30559-Gal4>UAS-Atg5-RNAi
Ref(2)P/P62
p-eIF2α
Tubulin
B
normalized p-eIF2α Protein level [% of control]
5d
ns
vt30559-Gal4/+
vt30559-Gal4>UAS-Atg5-RNAi
C
normalized Ref(2)P/P62 Protein level [% of control]
5d
**
D
vt30559-Gal4/+
vt30559-Gal4>UAS-Atg5-RNAi
5d
ATF4
Ref(2)P/P62
E
mean ATF4 intensity [% of control]
5d
ns
F
vt30559-Gal4>UAS-Atg5-RNAi
5d
ATF4
Ref(2)P/P62
G
me31b-GFP/+; vt30559-Gal4/+;
me31b-GFP/+; vt30559-Gal4>UAS-Atg5-RNAi
5d
GFP
H
mean GFP intensity [% of control]
5d
**
me31b-GFP/+; vt30559-Gal4/+
me31b-GFP/+; vt30559-Gal4>UAS-Atg5-RNAi
I
me31b-GFP/+;vt30559-Gal4>UAS-Atg5-RNAi
5d
GFP
Ref(2)P/P62

 © The Author(s)

Figure EV3. Related to Fig. 7, MB-specific *Atg5* knockdown does not induce global activation of the integrated stress response (ISR).

(**A–C**) Western blot analysis of Ref(2)P/P62, as well as of Ser51-phosphorylation levels of eIF2α in brain homogenates of 5-day-old female *vt30559*-Gal4>UAS-*Atg5*-RNAi flies in comparison to age- and sex-matched *vt30559*-Gal4/+ controls. (**A**) Representative Western blot displaying protein bands for Ref(2)P/P62, p-eIF2α and Tubulin as loading control. An amount equal to 2 brains was loaded for each lane. (**B**) Quantification of p-eIF2α levels. Band intensities were first normalized to the respective Tubulin band intensity, and all values were subsequently normalized to the *vt30559*-Gal4/+ control group. $n = 4$ independently prepared Western blot samples run together on the same gel; unpaired two-tailed *t*-test. ns not significant. (**C**) Quantification of Ref(2)P/P62 levels. Band intensities were first normalized to the respective Tubulin band intensity, and all values were subsequently normalized to the *vt30559*-Gal4/+ control group. $n = 4$ independently prepared Western blot samples run together on the same Gel; unpaired two-tailed *t*-test. $^{**}p = 0.0025$. (**D–F**) Confocal microscopy analysis of ATF4 and Ref(2)P/P62 in adult brains of 5-day-old female *vt30559*-Gal4>UAS-*Atg5*-RNAi flies and *vt30559*-Ga4/+ controls. (**D**) Representative confocal max projections of ATF4 and Ref(2)P/P62. (**E**) Quantification of whole brain mean ATF4 intensities in 5-day-old female flies. Scale bar = 50 µm; $n = 5$–6 adult brains per genotype; unpaired two-tailed *t*-test. ns not significant. (**F**) Representative confocal max projections of the anterior brain of a *vt30559*-Gal4>UAS-*Atg5*-RNAi fly, illustrating Ref(2)P/P62 aggregates with and without co-localization of ATF4. Scale bar left panel = 50 µm; Scale bar right panel = 25 µm. (**G–I**) Confocal microscopy analysis of endogenously tagged me31b-GFP in adult brains of 5-day-old female *me31b*-GFP/+;*vt30559*-Gal4>UAS-*Atg5*-RNAi flies and *me31b*-GFP;*vt30559*-Gal4/+ controls. (**G**) Representative confocal max projections of me31b-GFP fluorescent intensity. (**H**) Quantification of whole brain mean me31b-GFP intensities in 5-day-old female flies. Scale bar = 50 µm; $n = 12$–16 adult brains per genotype pooled from two independent experiments; unpaired two-tailed *t*-test. $^{**}p = 0.0066$. (**I**) Representative images of a single optical section of the anterior brain of a *vt30559*-Gal4>UAS-*Atg5*-RNAi fly, illustrating Ref(2 P)/P62 aggregates without observable accumulation of me31b-GFP signal. Scale bar left panel = 50 µm, Scale bar right panel = 5 µm. Data presented as mean ± SD. Source data are available online for this figure.

© The Author(s)

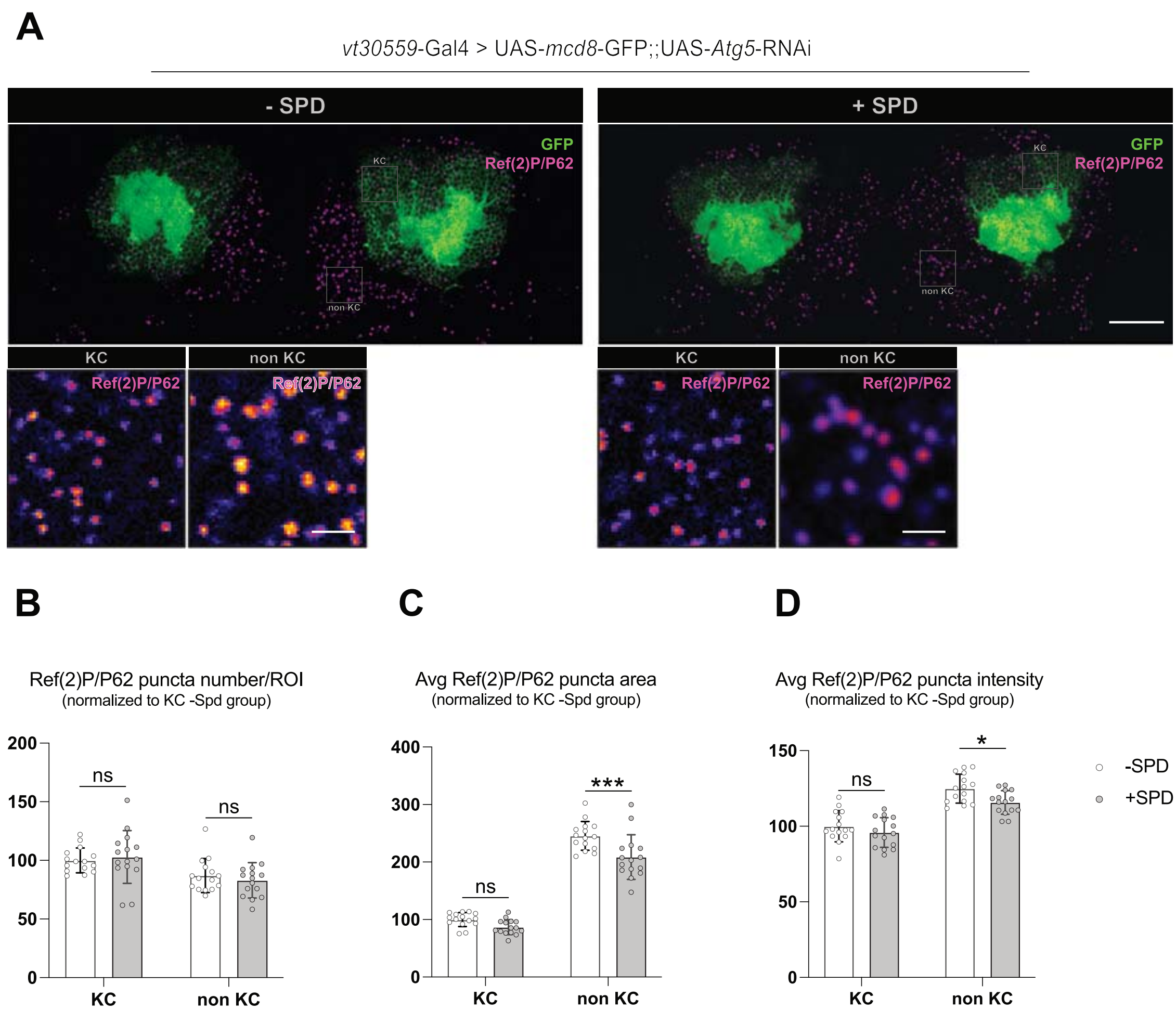

Figure EV4. Related to Fig. 8, 5 days spermidine (Spd) supplementation significantly reduces the size and intensity of non-cell autonomous Ref(2)P/P62 aggregates outside the MB KCs.

Freshly eclosed *vt30559*-Gal4>UAS-*mcd8*-GFP;;UAS-*Atg5*-RNAi flies were split at day 1 and either transferred onto 5 mM spermidine food or maintained on control food for 5 days. Female fly brains were subsequently dissected and immunostained against Ref(2)P/P62 for confocal microscopic analysis. (**A**) Representative single optical slices of the posterior MB calyx region, exemplifying chosen regions of interest (ROIs) for the analysis of Ref(2)P/P62 puncta in the MB Kenyon cells (KC) and in non-MB neurons (non-KC) of the same optical slice. MB KCs were identified based on the endogenous mcd8-GFP signal driven by *vt30559*-Gal4. Scale bar upper panels = 25 µm; Scale bar lower panels = 5 µm (**B–D**) Quantification of Ref(2)P/P62 puncta number (**B**), area (**C**), and intensity (**D**) for 5-day-old spermidine-treated (+Spd) and non-treated (-Spd) *vt30559*-Gal4>UAS-*mcd8*-GFP;;UAS-*Atg5*-RNAi flies. The average number, area and intensity of Ref(2)P/P62 spots per individual brain was calculated from a total number of 8 ROIs per each brain region (i.e., KC and non-KC), retrieved from two individual optical slices. Measured data from two independent experiments was pooled by normalizing the measured values onto the KC – Spd group. Only relevant statistical comparisons between Spd-supplemented (+Spd) and untreated (−Spd) groups are displayed. The selection of ROIs and suitable optical slices was performed blinded. $n = 15$ adult brains per treatment, pooled from two independent experiments. One-way ANOVA with Tukey's post hoc test for multiple comparisons. *$p = 0.0487$, ***$p = 0.0009$, ns not significant. Data presented as mean ± SD. Source data are available online for this figure.

 © The Author(s)
