# Supplementary material for: Local autophagy impairment triggers brain-wide presynaptic remodeling and resilience
Source: EMBO J. 2026 Jul 8;45(17):6054–85. doi: 10.1038/s44318-026-00847-4 (PMC13534471; doi:10.1038/s44318-026-00847-4)
Supplement: Supplementary file 2 [file 44318_2026_847_MOESM1_ESM.pdf]

Appendix for:

"Local autophagy impairment triggers brain-wide presynaptic remodeling and resilience"

1	.	Appendix Figure S1	–	Related to	Fig. 1	Knockdown of *Atg5* in sleep-regulating neurons does not induce brain-wide PreScale.	**1**
2	.	Appendix Figure S2	–	Related to	Fig. 3	Pan-neuronal or MB-specific re-expression of *Atg7* improves the survival of $Atg7^{d77}$ mutants.	**3**
3		Appendix Figure S3		Related to	Fig. 4	Post-developmental Auxin-induced expression of *Atg5*-RNAi within the MB KCs does not result in Ref(2)P/P62 aggregate formation at 15 days.	**4**
4	.	Appendix Figure S4	–	Related to	Fig. 4	Gal4-controlled activation of BRP FlySam2.0 robustly increases BRP levels at the larval neuromuscular junction (NMJ) and in the adult brain.	**5**
5	.	Appendix Figure S5	–	Related to	Fig. 6	Volcano plots illustrating the Top 20 protein hits with the highest p-value significantly downregulated or upregulated in MB-autophagy impaired flies compared to controls.	**6**
6	.	Appendix Figure S6	–	Related to	Fig. 6	Proteomic changes in fly brains with MB-specific *Atg5* knockdown are largely mediated post-transcriptionally.	**7**
7	.	Appendix Figure S7	–	Related to	Fig. 7	Somatic CRISPR/Cas9-mediated conditional knockout of *Atg5* specifically in MB KCs results in widespread non-cell autonomous accumulation of Ref(2)P/P62.	**8**
8	.	Appendix Figure S8	–	Related to	Fig. 8	Synaptic accumulation of ATG8a in the MB lobes upon *vt30559*-Gal4 mediated knockdown of *Atg5.*	**9**
9	.	Appendix Table S1	–	Related to	Fig. 5,6	Significantly upregulated proteins upon MB-specific *Atg5* knockdown.	**10**
10	.	Appendix Table S2	–	Related to	Fig. 5,6	Significantly downregulated proteins upon MB-specific *Atg5* knockdown.	**18**

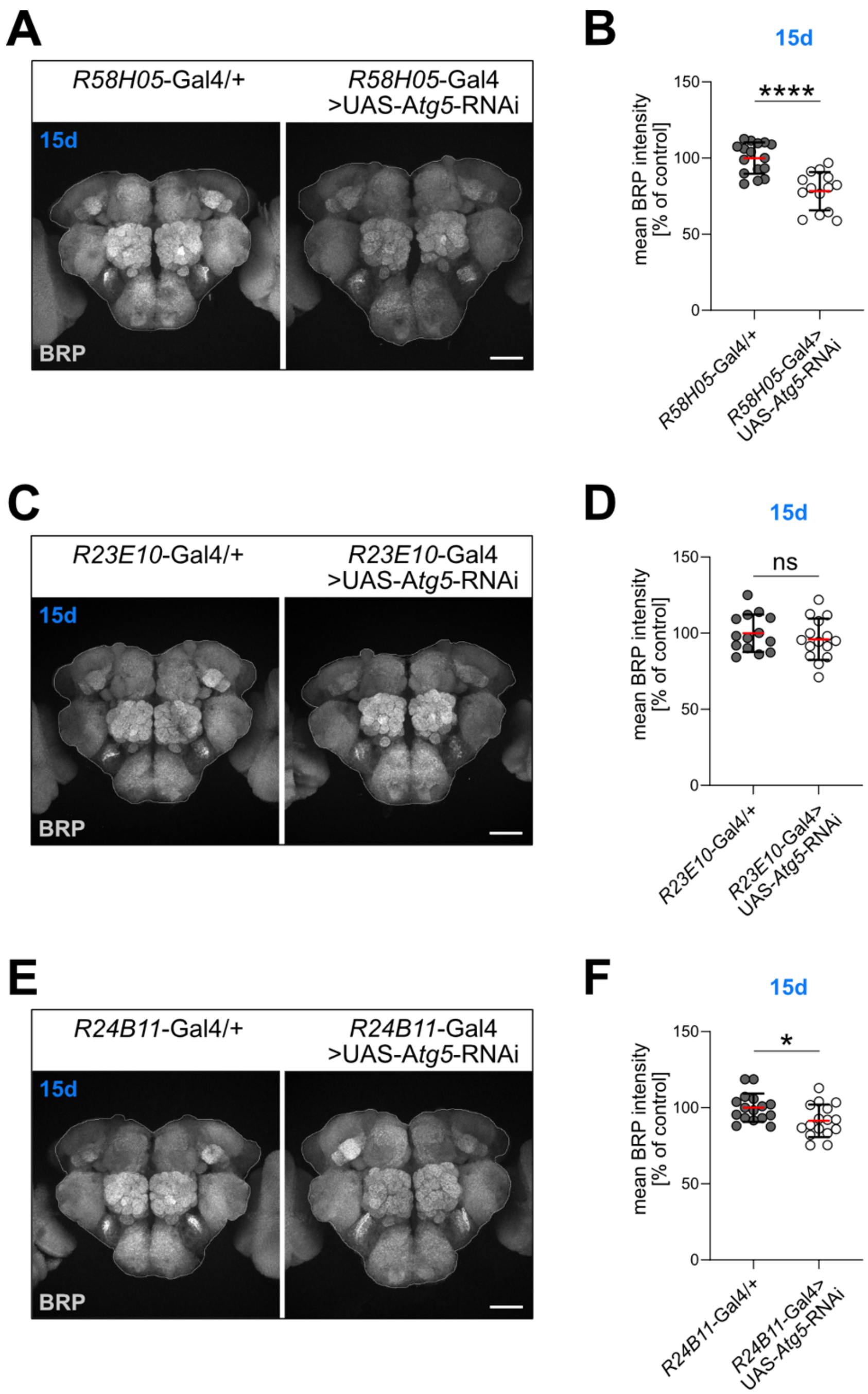

Appendix Figure S1 – related to Fig. 1

Knockdown of *Atg5* in sleep-regulating neurons does not induce brain-wide PreScale.

(A, B) Knockdown of *Atg5* in R2/5 neurons of the ellipsoid body (*R58H05*-Gal4>UAS-*Atg5*-RNAi) in comparison to *R58H05*-Gal4/+ female control flies at 15d age. Brains were immunostained against BRP^{Nc82}, and measured whole brain mean BRP intensities were normalized to the *R58H05*-Gal4/+ control group. **(A)** Representative confocal max projections and **(B)** quantification of BRP intensities for 15d old female flies. Scale bar = 50 µm; n = 14-16 adult brains per genotype pooled from 2 independent experiments; unpaired Two-tailed t-test. ****p <0.0001. **(C, D)**

Knockdown of *Atg5* in dorsal-fan-shaped body neurons (*R23E10*-Gal4>UAS-*Atg5*-RNAi) in comparison to *R23E10*-Gal4/+ female control flies at 15d age. Brains were immunostained against BRP^{Nc82}, and measured whole brain mean BRP intensities were normalized to the *R23E10*-Gal4/+ control group. **(C)** Representative confocal max projections and **(D)** quantification of BRP intensities for 15d old female flies. Scale bar = 50 µm; n = 14-15 adult brains per genotype pooled from 2 independent experiments; unpaired Two-tailed t-test. ns, not significant. **(E, F)** Knockdown of *Atg5* in helicon cells (*R24B11*-Gal4>UAS-*Atg5*-RNAi) in comparison to *R24B11*-Gal4/+ female control flies at 15d age. Brains were immunostained against BRP^{Nc82}, and measured whole brain mean BRP intensities were normalized to the *R24B11*-Gal4/+ control group. **(E)** Representative confocal max projections and **(F)** quantification of BRP intensities for 15d old female flies. Scale bar = 50 µm; n = 16 adult brains per genotype pooled from 2 independent experiments; unpaired Two-tailed t-test. *p = 0.0210. Data presented as mean ± SD.

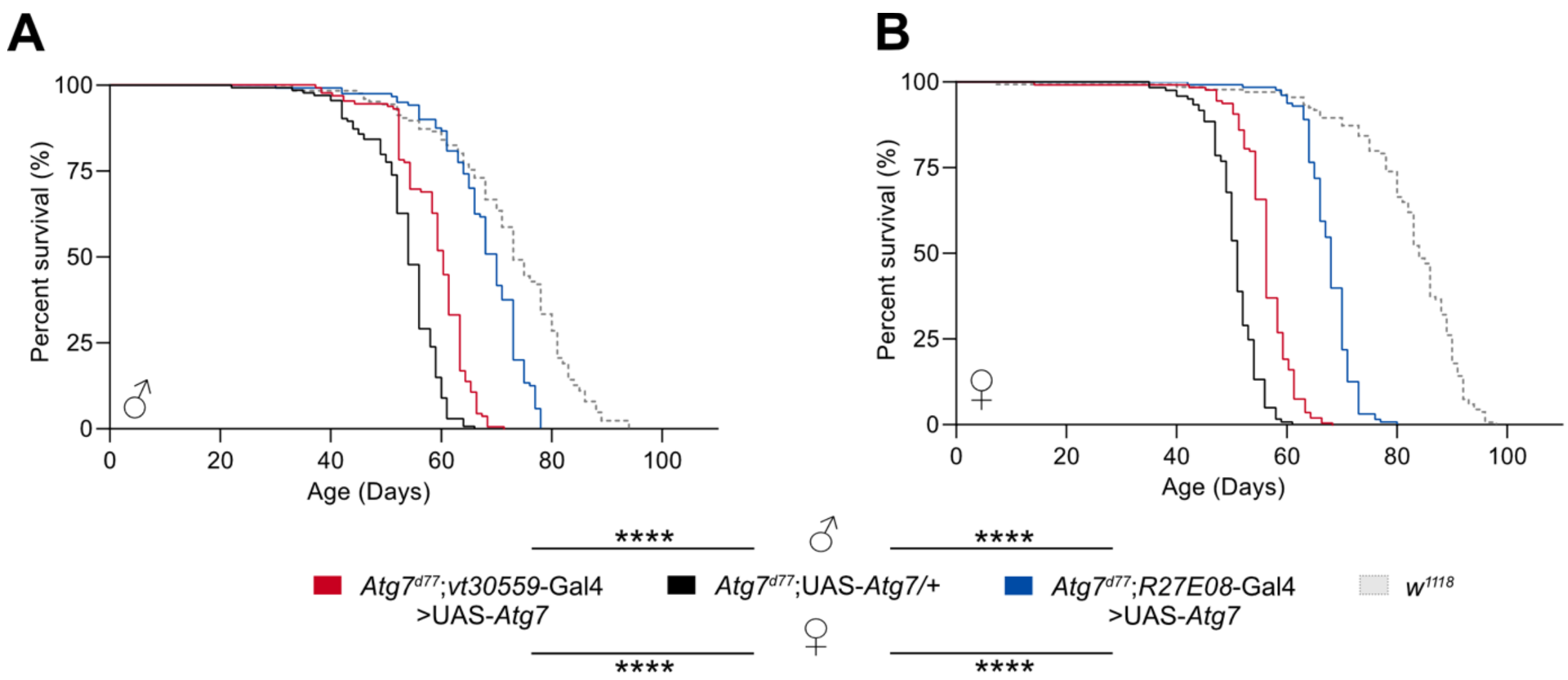

Appendix Figure S2 – related to Fig. 3

Pan-neuronal or MB-specific re-expression of *Atg7* improves the survival of *Atg7*^{d77} mutants.

(A and B) Kaplan-Meier survival curves of male **(A)** and **(B)** female *Atg7*^{d77} flies with either pan-neuronal (*Atg7*^{d77}*;R27E08*-Gal4>UAS-*Atg7*) or MB KC-specific (*Atg7*^{d77}*;vt30559*-Gal4>UAS-*Atg7*) re-expression of *Atg7* compared to *Atg7*^{d77};UAS-*Atg7*/+ control flies. Survival curves of *w*^{1118} was plotted as a wildtype control group. n = 126-135 flies per genotype and sex. Pairwise Log-rank (Mantel-Cox) test comparison. ****p <0.0001.

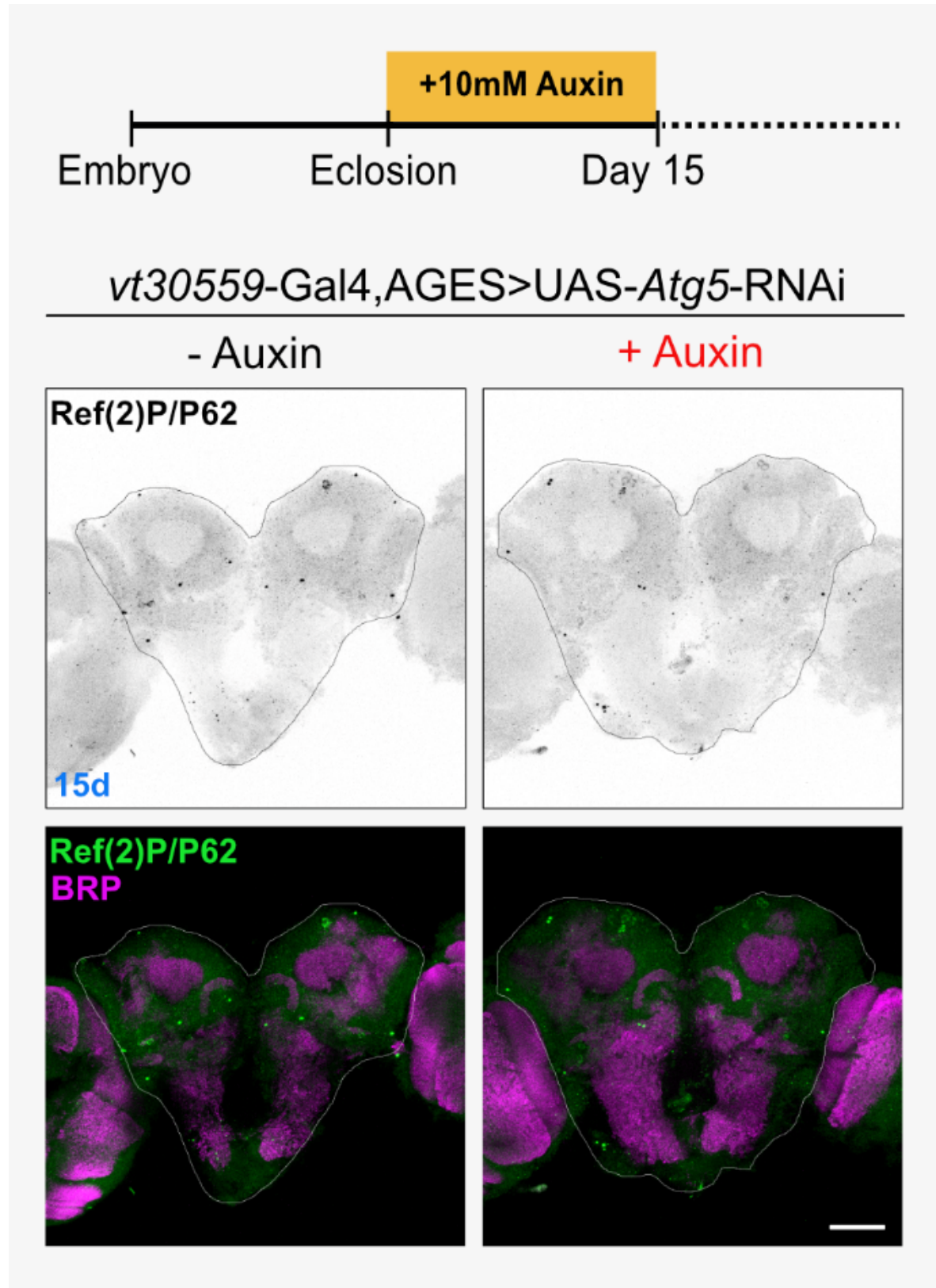

Appendix Figure S3 – related to Fig. 4

Post-developmental Auxin-induced expression of *Atg5*-RNAi within the MB KCs does not result in Ref(2)P/P62 aggregate formation at 15 days.

Representative confocal max projections of the posterior part of 15d old female fly brains, including the MB calyx region with MB KC cell bodies. *vt30559*-Gal4**,**AGES>UAS-*Atg5*-RNAi flies originating from the same cross were either supplemented with 10mM Auxin between day 1 and day 15 post-eclosion (+Auxin), or maintained on standard food (-Auxin) to investigate the effects of post-developmentally induced expression of *Atg5*-RNAi within the MB KCs. Ref(2)P/P62 immunostaining was used as molecular marker for assessing autophagy impairment, and the BRP^{Nc82} co-staining was employed to identify the MB calyx region. Scale bar = 50µm.

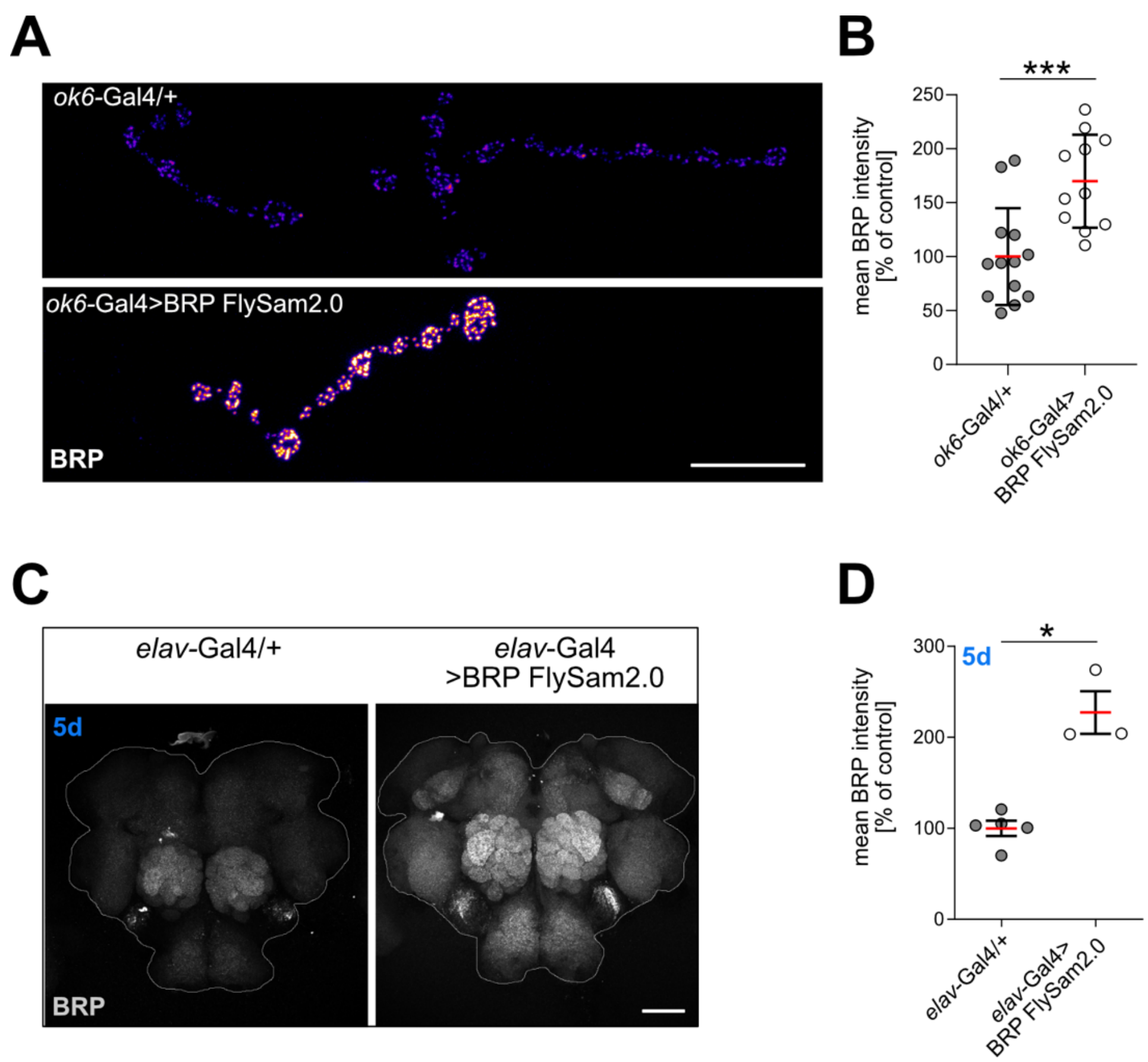

Appendix Figure S4 – related to Fig.4

Gal4-controlled activation of BRP FlySam2.0 robustly increases BRP levels at the larval neuromuscular junction (NMJ) and in the adult brain.

(A) Representative confocal Max projections of BRP signal at larval NMJs upon motor-neuron specific activation of BRP FlySam2.0 (*ok6*-Gal4>BRP FlySam2.0) in comparison to *ok6*-Gal4/+ controls. Larval filets were immunostained against BRP^{Nc82}. Scale bar = 15 µm **(B)** Quantification of BRP intensity at larval NMJs, normalized to *ok6*-Gal4/+ controls. n = 11-13 NMJs per genotype acquired from 4 animals per genotype. Unpaired Two-tailed t-test. ***p = 0.0008 **(C)** Representative confocal Max projections of BRP levels in 5d old female adult *Drosophila* brains upon pan-neuronal activation of BRP FlySam2.0 (*elav*-Gal4>BRP FlySam2.0) in comparison to age-matched *elav*-Gal4/ controls. Brains were immunostained against BRP^{Nc82}. Scale bar = 50 µm. **(D)** Quantification of whole brain mean BRP intensities, normalized to the *elav*-Gal4/+ control group. n = 3-5 brains per genotype. Mann-Whitney test. *p = 0.0357; Data presented as mean ± SD.

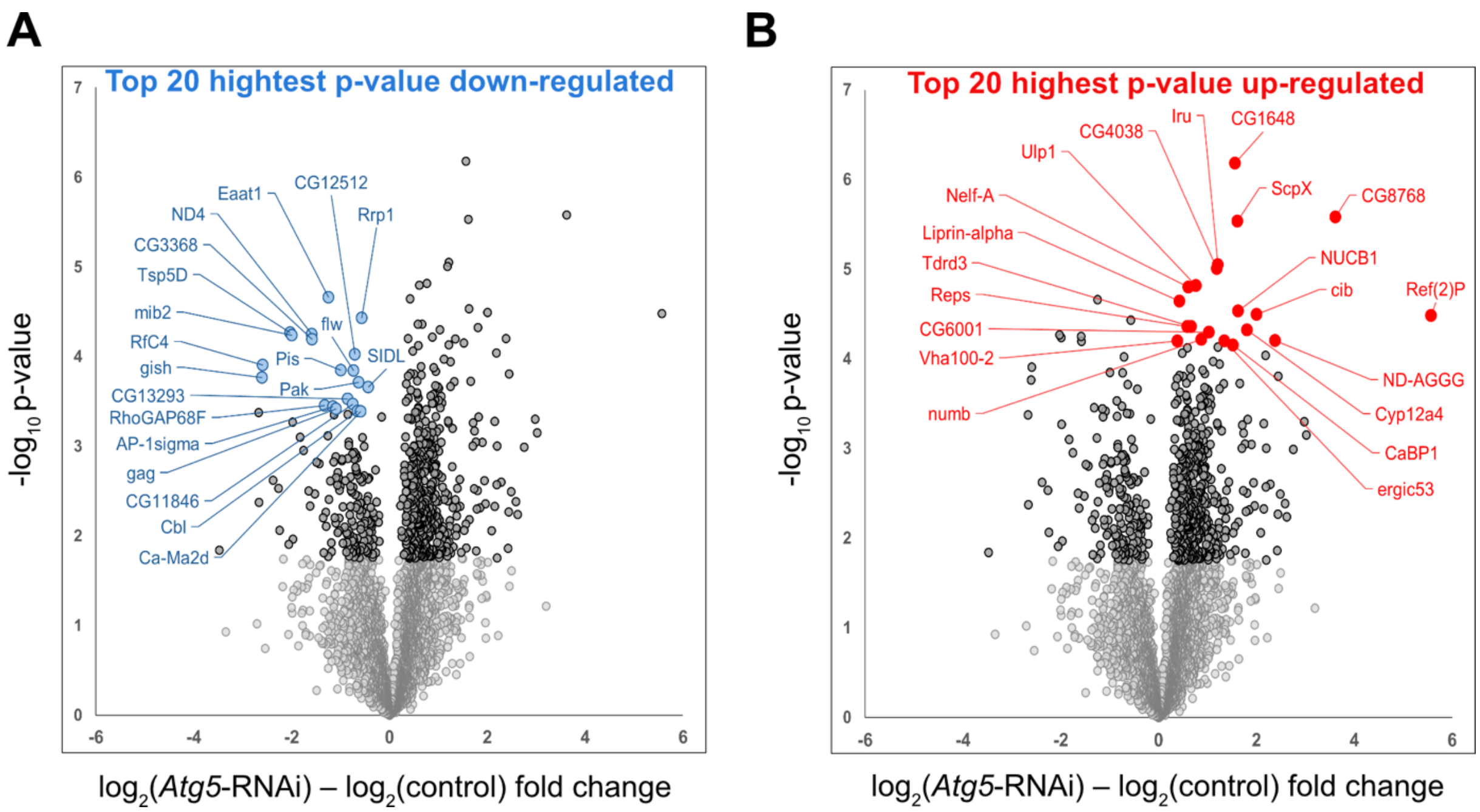

Appendix Figure S5 – related to Fig. 6

Volcano plots illustrating the Top 20 protein hits with the highest p-value significantly downregulated or upregulated in MB-autophagy impaired flies compared to controls.

(A, B) Volcano plots of all proteins identified in the proteomic data set, highlighting the Top20 downregulated **(A)** and upregulated **(B)** protein hits with the highest p-value identified in 10d old *ok107*-Gal4>UAS-*Atg5*-RNAi flies compared to age-matched *ok107*-Gal4/+ controls. The Log_2 fold change on the x-axis is plotted against the $-\text{Log}_{10}$ p-value of the two-tailed t-test comparison on the y-axis. A $-\log_{10}$ p-value cut-off of 1.75 was used to identify significantly changed protein hits (dark grey points), identified proteins below this cutoff are depicted as light grey points. n = 3-4 biological replicates (10 dissected brains per replicate) per genotype.

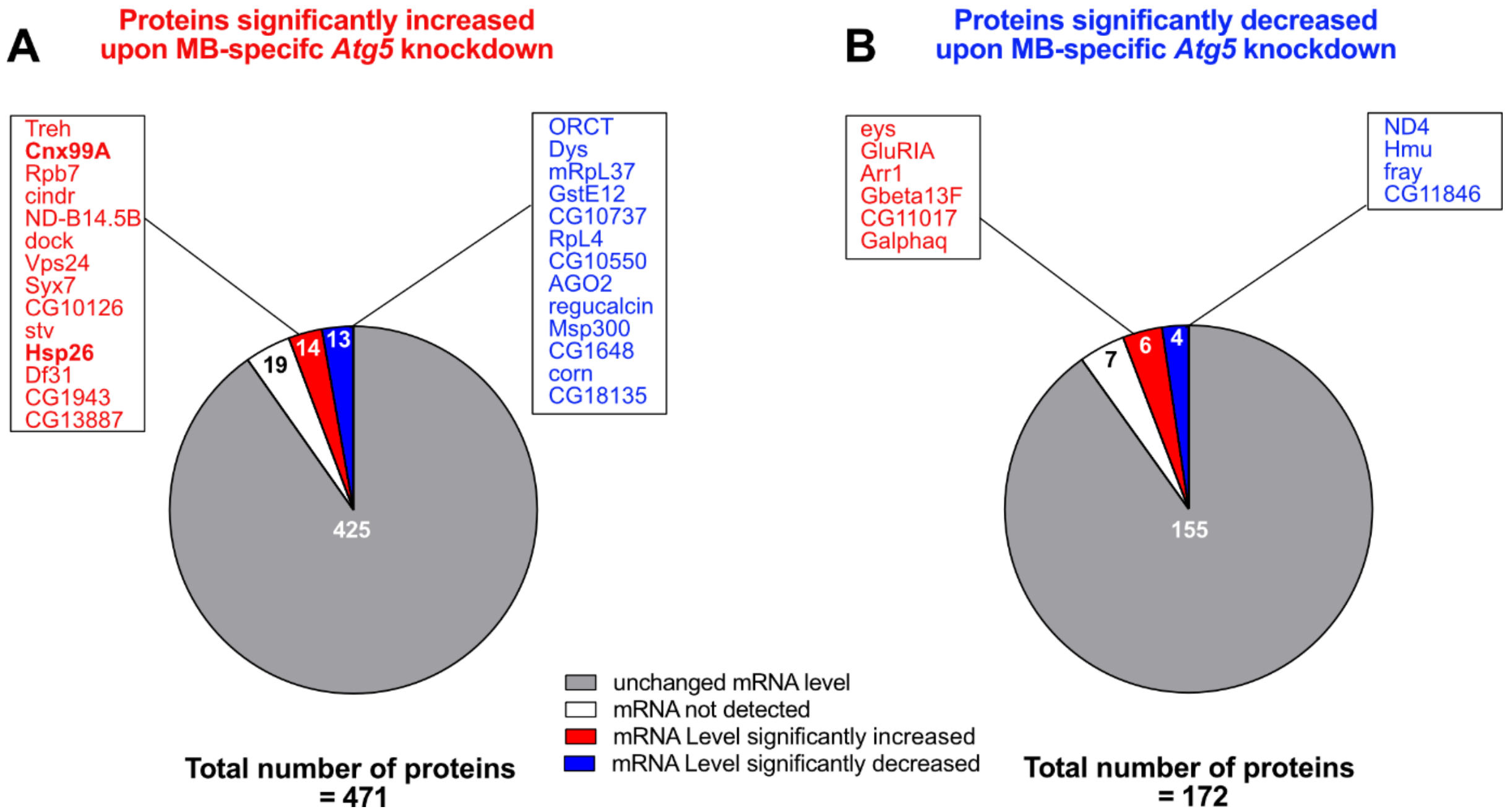

Appendix Figure S6 – related to Fig. 6

Proteomic changes in fly brains with MB-specific *Atg5* knockdown are largely mediated post-transcriptionally. Correlation of all significantly increased **(A)** or decreased **(B)** proteins in *ok107*-Gal4>UAS-*Atg5*-RNAi fly brains compared to *ok107*-Gal4/+ controls detected in the proteomic experiment with the data set acquired by RNA-Seq. Candidates with significantly changed expression at mRNA and protein level are depicted in the boxes.

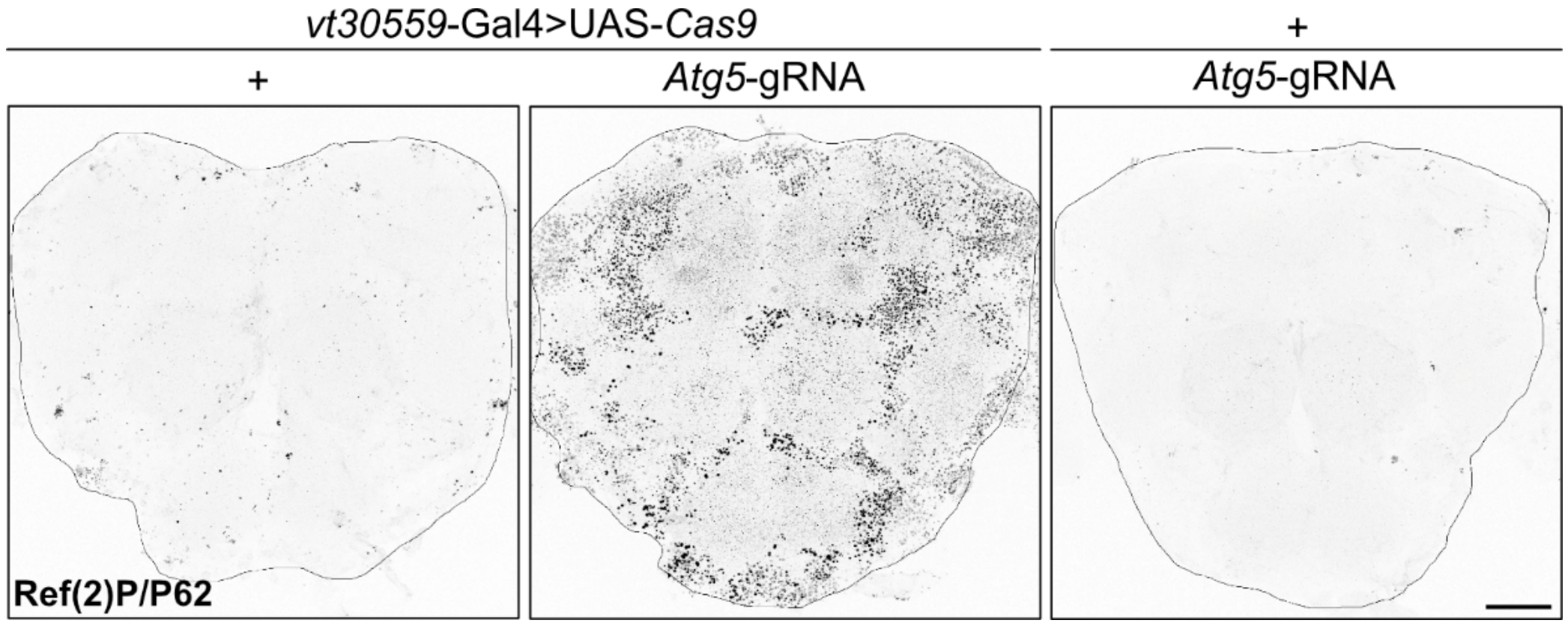

Appendix Figure S7 – related to Fig. 7

Somatic CRISPR/Cas9-mediated conditional knockout of *Atg5* specifically in MB KCs results in widespread non-cell autonomous accumulation of Ref(2)P/P62.

Representative confocal max projections of whole-mount 5d old female fly brains immunostained against Ref(2)P/P62. The expression of *Cas9* in the MB under control of *vt30559*-Gal4 and the simultaneous presence of an ubiquitously expressed *Atg5*-gRNA induces site-directed DNA double stand breaks into the *Atg5* gene exclusively in the MB KCs. Scale bar = 50 µm.

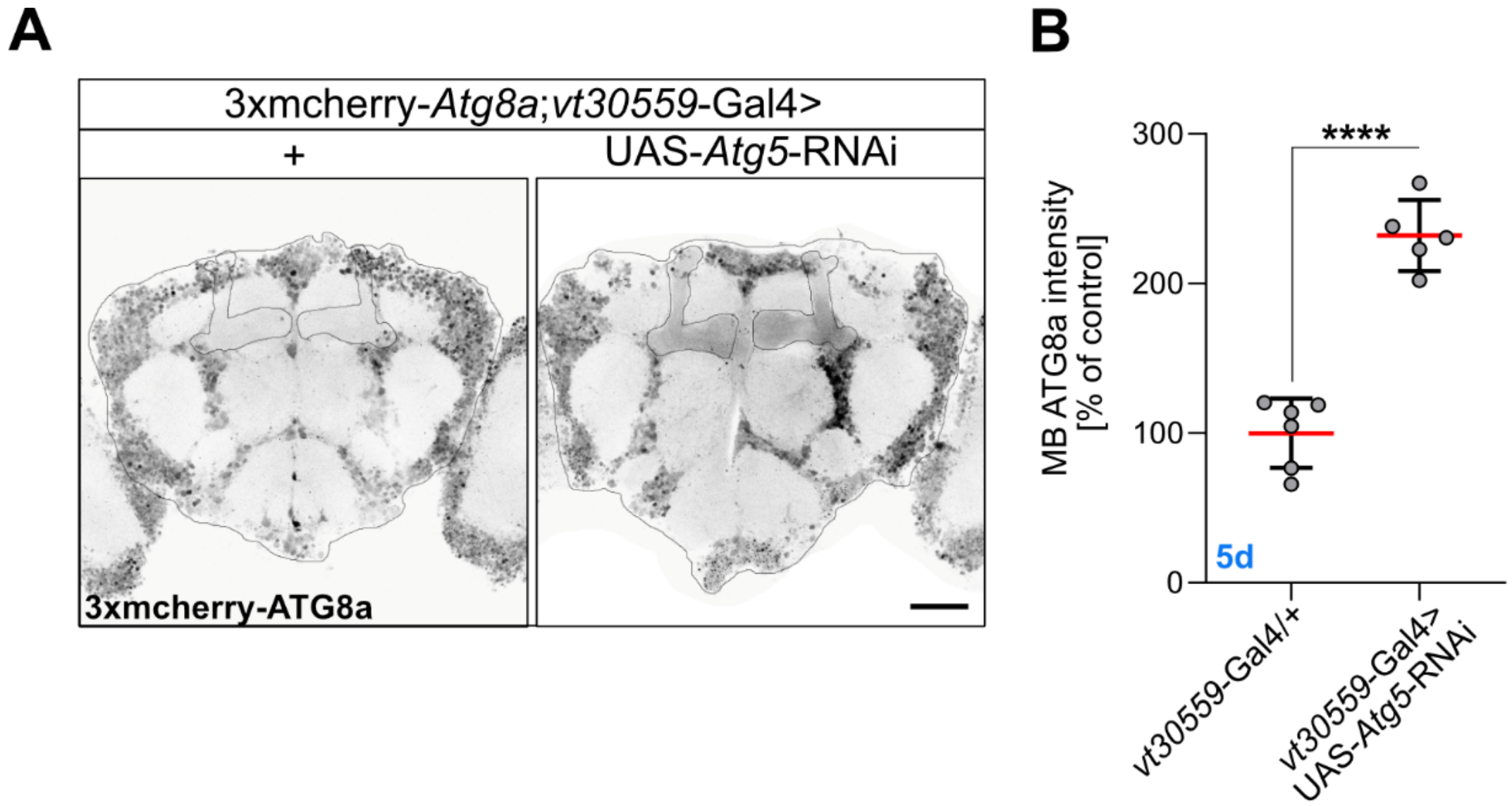

Appendix Figure S8 – related to Fig. 8

Synaptic accumulation of ATG8a in the MB lobes upon *vt30559*-Gal4 mediated knockdown of *Atg5*.

(A) Representative confocal max projections of the fluorescent signal of the endogenous promoter-driven 3xmCherry-*Atg8a* reporter in *vt30559*-Gal4>UAS-*Atg5*-RNAi and *vt30559*-Gal4/+ control brains, imaged from the anterior side. Scale bar = 50 µm **(B)** Quantification of the 3xmCherry-ATG8a fluorescent signal inside the MB lobes. Representative ROIs surrounding the MB lobes are depicted in (A). n = 5-6 MB lobes analyzed per genotype. Unpaired two-tailed t-test. ****p <0.0001. Data presented as mean ± SD.

Appendix Table S1 – related to Fig. 5+6

All significantly upregulated proteins upon MB-specific *Atg5* knockdown

Protein ID	Flybase	Gene Name	Student's T-test Difference ATG5 RNAi_ctrl	-Log Student's T-test p-value ATG5 RNAi_ctrl
Q7K2P3	FBgn0033446	**CG1648**	1.559679508	6.180024352
Q8T0N5	FBgn0033769	**CG8768**	3.61668396	5.581058227
Q9VJ43	FBgn0015808	**ScpX**	1.609803677	5.531523801
Q9VHI7	FBgn0037653	**Iru**	1.212962151	5.04810325
A8E6K0	FBgn0011824	**CG4038**	1.186741829	5.004553488
Q9VWK5	FBgn0027603	**Ulp1**	0.76397419	4.81542428
A0A0B4LIF3	FBgn0038872	**Nelf-A**	0.611036301	4.79610967
Q9VM93	FBgn0046704	**Liprin-alpha**	0.42342186	4.64150071
Q1EC31	FBgn0052190	**NUCB1**	1.62173748	4.531294878
O97428	FBgn0026084	**cib**	2.001122475	4.491889754
P14199	FBgn0003231	**ref(2)P**	5.565166473	4.479433949
M9NDL7	FBgn0032341	**Reps**	0.584844589	4.360461509
Q9VUH8	FBgn0036450	**Tdrd3**	0.666800499	4.359333277
Q9VE00	FBgn0038681	**Cyp12a4**	1.807024479	4.321889975
A0A0B4LHR2	FBgn0051064	**CG6001**	1.030639648	4.296598375
M9PCN6	FBgn0002973	**numb**	0.877635479	4.214773382
Q7PL91	FBgn0058002	**ND-AGGG**	2.377919674	4.200582033
Q9VE75	FBgn0028670	**Vha100-2**	0.381268978	4.199039139
Q9V3A8	FBgn0035909	**ergic53**	1.343855858	4.196771926
Q9V438	FBgn0025678	**CaBP1**	1.51931715	4.149502417
Q9Y0Y5	FBgn0027496	**epsilonCOP**	1.210440159	4.129888924
Q59E58	FBgn0265434	**zip**	0.586822987	4.122669328
Q7KQM6	FBgn0039936	**CG11148**	0.479780674	4.061450418
Q9VIU4	FBgn0053116	**CG33116**	2.181749344	4.039069212
Q9V3N6	FBgn0028541	**TM9SF4**	1.113148689	3.971117532
Q9VXV6	FBgn0040285	**Scamp**	1.249063492	3.941331222
Q95NR4	FBgn0032679	**bsf**	0.642412186	3.888312632
F6JQA6	FBgn0025615	**Torsin**	0.902486324	3.873920986
Q8IQW5	FBgn0031037	**HspB8**	0.942337513	3.833654895
Q59DX2	FBgn0266756	**btsz**	0.428268433	3.811835146
O97417	FBgn0261397	**didum**	0.496436119	3.805379661
Q9VUB8	FBgn0061515	**endos**	2.446491718	3.805073215
Q2PDY2	FBgn0013263	**Trl**	0.654875755	3.792876982
B5X533	FBgn0030706	**Lrp4**	0.376559734	3.779355352
Q9VDW3	FBgn0260003	**Dys**	0.56616354	3.7273028
Q7JR91	FBgn0013305	**Nmda1**	1.657114029	3.724564358
Q8I7Q0	FBgn0025876	**gag**	1.326896667	3.720246429
Q9W347	FBgn0040234	**c12.2**	0.344968319	3.693387684
Q9VK58	FBgn0032455	**Pih1D1**	0.940951824	3.676218379
Q9VHN6	FBgn0037608	**mRpL19**	1.249351501	3.666720425
Q8IR92	FBgn0265595	**CG42683**	0.989835262	3.661289575
Q9VUQ5	FBgn0087035	**AGO2**	1.01206398	3.645359781
Q7YZA4	FBgn0025641	**DAAM**	0.432307243	3.62724248
E1JHM0	FBgn0032821	**CdGAPr**	0.560956478	3.595402446
M9PFF1	FBgn0020655	**ArfGAP1**	0.569808483	3.580061658
Q9V8R9	FBgn0010434	**cora**	0.886549473	3.575622388
Q7KW39	FBgn0023537	**CG17896**	0.843849182	3.560632842
Q9W254	FBgn0022985	**qkr58E-2**	1.058598042	3.555883477
Q9VP80	FBgn0026179	**siz**	0.58856678	3.538436093
A0A0B4K7G3	FBgn0039883	**RhoGAP100F**	0.38231945	3.533062181
E1JIL7	FBgn0262483	**Rbp**	0.382414818	3.531500591
Q9VWK6	FBgn0030997	**CG7990**	0.935040951	3.515947695
Q9VTG0	FBgn0260795	**NaPi-III**	1.008313656	3.509472708
Q9U6X3	FBgn0027951	**MTA1-like**	0.336732388	3.497208386
X2J4W8	FBgn0266449	**Kr-h2**	1.070585251	3.482871574
M9PE74	FBgn0261710	**nocte**	0.899382114	3.477483467
Q9VF20	FBgn0038376	**Hmt-1**	0.870473862	3.475827291
Q9V3Y5	FBgn0028474	**CG4119**	0.589488029	3.432835374

Protein ID	Flybase	Gene Name	Student's T-test Difference ATG5 RNAi_ctrl	-Log Student's T-test p-value ATG5 RNAi_ctrl
M9PC74	FBgn0031673	**CG9112**	1.033864975	3.429007549
M9MS31	FBgn0261549	**rdgA**	0.581436157	3.416742008
Q3YMU0	FBgn0033663	**ERp60**	0.750228882	3.409169161
Q8SYN6	FBgn0035830	**CG8209**	0.528584957	3.381090575
A1ZBE9	FBgn0034401	**MetRS**	0.560018063	3.376626071
A0A126GUM8	FBgn0020279	**lig**	0.644701958	3.347122266
Q9VTJ4	FBgn0036169	**Fuca**	0.947486401	3.33030136
B7Z107	FBgn0259722	**CG42376**	1.820374489	3.324892932
M9PDH4	FBgn0023531	**CG3791**	0.378716469	3.315442764
P17972	FBgn0003386	**Shaw**	0.546954155	3.313341192
Q9VGG9	FBgn0051368	**CG14729**	0.426767826	3.31081168
Q8IHB6	FBgn0261625	**GLS**	2.975390434	3.299158023
M9NFP7	FBgn0260400	**elav**	0.457743645	3.295291727
Q9VXU2	FBgn0027287	**Gmap**	1.199504852	3.279206482
X2JD55	FBgn0004045	**Yp1**	2.152569771	3.27741127
Q9VC40	FBgn0039223	**CG5805**	0.736559868	3.274763598
O77134	FBgn0038224	**ATPsynE**	1.707364559	3.259407169
Q9VI56	FBgn0037468	**CG1943**	1.20650959	3.259306525
Q8SY33	FBgn0051992	**gw**	0.608628273	3.235509298
Q7KUX7	FBgn0262734	**eIF4H1**	0.873892784	3.23005479
Q9VS57	FBgn0035771	**Sec63**	1.286948681	3.198097796
Q8T7V5	FBgn0028875	**nAChRalpha5**	1.023607254	3.193945326
A8JR03	FBgn0085412	**kmr**	0.59426403	3.172360952
Q7JVK8	FBgn0034362	**CG5323**	1.73666811	3.169868087
Q9VB96	FBgn0051075	**CG31075**	0.766214371	3.166379099
Q7KNS5	FBgn0022987	**qkr54B**	0.969668865	3.161912077
Q9VDY8	FBgn0267975	**vib**	3.021068573	3.151133786
X2JEC4	FBgn0085446	**CG3960**	0.872220516	3.151005793
Q7KV90	FBgn0010909	**msn**	0.572367191	3.149818421
Q86BS3	FBgn0044324	**Chro**	0.698696613	3.147203865
Q86BI3	FBgn0263603	**Zn72D**	0.763814926	3.122977457
A4V4W0	FBgn0016976	**stnA**	0.330391407	3.105677381
Q8IM93	FBgn0052017	**CG32017**	0.912090778	3.091064188
Q1RKQ9	FBgn0085382	**CG5566**	0.617788792	3.083077432
E1JIH1	FBgn0051352	**Unc-115a**	0.828317642	3.078103987
X2JAZ6	FBgn0036165	**chrb**	1.765043259	3.047248936
Q9I7U2	FBgn0035321	**CG1275**	1.054427624	3.044494124
Q9VYU9	FBgn0030332	**CG9360**	1.896373272	3.043457542
Q9VU17	FBgn0036316	**nebu**	0.820839405	3.03585592
Q8IPA7	FBgn0052831	**CG16917**	0.802610874	3.031916799
Q9VXK9	FBgn0030716	**CG9170**	0.700047016	3.031558349
E0WN35	FBgn0015519	**nicra3**	0.740230083	3.022684008
Q8SX89	FBgn0038476	**kuk**	0.782693863	3.021672735
A8JQV3	FBgn0037525	**CG17816**	0.603003502	3.013520626
A0A0B4KEK7	FBgn0033669	**Pl31**	0.856729031	3.010046415
H8F4P8	FBgn0000479	**dnc**	0.502356052	3.003696616
Q9VVV6	FBgn0036837	**CG18135**	2.19914341	3.002477943
M9PEM7	FBgn0028582	**lqf**	0.86951828	3.00130679
Q9VV95	FBgn0036656	**CG13026**	1.703688622	2.999700345
Q7JWU9	FBgn0034242	**CG14480**	0.494612694	2.99772878
P38040	FBgn0004921	**Ggamma1**	2.750299931	2.98945408
P13496	FBgn0001108	**DCTN1-p150**	0.31874609	2.98944294
Q7K0X9	FBgn0267849	**Syx7**	0.938266754	2.980470332
M9PBG8	FBgn0264707	**RhoGEF3**	0.522706985	2.970263141
Q9VVS6	FBgn0036813	**Atg3**	0.85214138	2.963735304
Q9VBK9	FBgn0039385	**CG5913**	0.528435707	2.958349348
Q9VRE0	FBgn0031149	**Stt3A**	0.569153309	2.94967878
M9PBN7	FBgn0011570	**cpb**	1.010475636	2.944980813
A0A0B4K7K9	FBgn0259246	**brp**	0.43570137	2.940144833
P17210	FBgn0001308	**Khc**	0.660561562	2.936903837
M9NFH8	FBgn0036663	**GS**	0.409172058	2.933736617
Q9VH98	FBgn0012344	**Dh44**	0.888766766	2.932098268
Q8IRB9	FBgn0264693	**ens**	0.826870441	2.928297094

Protein ID	Flybase	Gene Name	Student's T-test Difference ATG5 RNAi_ctrl	-Log Student's T-test p-value ATG5 RNAi_ctrl
Q9W2V2	FBgn0052683	**CG15303**	0.458001614	2.913727523
Q9W0S7	FBgn0035121	**Tudor-SN**	0.625941277	2.911511925
A0A126GUS4	FBgn0053208	**Mical**	0.88074255	2.910329588
Q7K126	FBgn0033029	**Not3**	0.816794872	2.908519076
Q9Y111	FBgn0026206	**mei-P26**	0.911016464	2.886774099
Q24319	FBgn0014868	**Ost48**	0.710199356	2.884303316
Q9VEJ5	FBgn0261046	**Dscam3**	0.479027748	2.882004846
A0A0B4LGZ0	FBgn0051352	**Unc-115a**	0.479701996	2.873421942
O62621	FBgn0025724	**beta'COP**	0.740339279	2.863940633
X2JGR4	FBgn0000017	**Abl**	0.663938999	2.863803771
Q9W0H8	FBgn0025682	**scf**	0.61348772	2.85281839
P42787	FBgn0004648	**svr**	0.567816257	2.851141936
A0A0B4KGN2	FBgn0019960	**Mitofilin**	0.49845171	2.844339708
Q7YU85	FBgn0027598	**cindr**	0.588870525	2.829385598
Q709R6	FBgn0037719	**bocks**	0.747193813	2.828057286
A4V2I0	FBgn0000071	**Ama**	0.655789852	2.826978992
P13709	FBgn0004656	**fs(1)h**	0.784294605	2.816099575
Q8WR08	FBgn0034072	**Dg**	0.490888596	2.813910956
X2JFR1	FBgn0004227	**nonA**	0.547379971	2.810875499
A0A0B4LHT3	FBgn0265274	**Inx3**	0.778326035	2.808978624
Q9VBU5	FBgn0039304	**CG10425**	0.624582767	2.79829521
Q9GPQ1	FBgn0033520	**Prx2540-1**	0.952177525	2.796132473
Q7KTU2	FBgn0052451	**SPoCk**	0.305841923	2.787593556
Q9VHA8	FBgn0037718	**P58IPK**	0.72527647	2.78454446
B5RIU6	FBgn0038659	**EndoA**	0.454913616	2.78386418
Q9VNA2	FBgn0013576	**mtd**	0.418243408	2.780400559
Q9VA97	FBgn0039751	**CG1983**	0.758174896	2.780033755
Q8T0Q4	FBgn0086656	**shrb**	0.938057899	2.778002686
Q4QQ70	FBgn0030694	**CG15602**	1.283683777	2.771654407
E6PBV6	FBgn0030151	**CG1354-RA**	0.927331924	2.76660958
Q24523	FBgn0259176	**bun**	0.539965153	2.757590193
Q9W384	FBgn0030093	**Bap111**	0.500801563	2.754579862
M9PEU6	FBgn0264305	**CG5741**	0.394497395	2.753894046
Q9VXI3	FBgn0030734	**CG9911**	0.797796726	2.753124527
Q9W1R3	FBgn0034854	**Golgin245**	0.813738823	2.751201099
H1UUG6	FBgn0033846	**mip120**	0.759030342	2.744914787
Q7JWI7	FBgn0033028	**hrm**	0.731140137	2.740638517
Q9NAZ4	FBgn0053126	**NLaz**	0.505874634	2.732007116
A8DYK5	FBgn0034598	**cg4266**	0.561712742	2.725598017
A0A126GUN3	FBgn0267002	**unc-104**	0.301437378	2.721088337
Q9V9N5	FBgn0261802	**CG42748**	0.720464706	2.71586099
Q7JRC3	FBgn0010053	**Jheh1**	0.519627571	2.705972976
A0A0F6QCW0	FBgn0013432	**bcn92**	0.949622631	2.704491976
M9MS14	FBgn0025631	**moody**	0.915418148	2.699320236
X2JGP4	FBgn0014002	**Pdi**	0.651749134	2.692496197
Q9VNA3	FBgn0037312	**CG11999**	0.999297619	2.683665325
A0AN20	FBgn0031817	**CG9531**	0.434117317	2.67318379
O61613	FBgn0020392	**Nmt**	0.54317522	2.670900887
A0A126GUM1	FBgn0263102	**psq**	0.301305294	2.660265953
Q9VYR1	FBgn0030362	**regucalcin**	1.072425842	2.653181747
Q8T9D5	FBgn0030478	**CG1640**	0.569777489	2.647864758
B7YZL7	FBgn0034420	**CG10737**	0.590616226	2.637989985
Q9U4F3	FBgn0029117	**Surf1**	1.435103416	2.627506066
Q9VC66	FBgn0039204	**CG6607**	0.509538174	2.626528825
E1JHT2	FBgn0011603	**ine**	0.709859848	2.615839609
Q9VR57	FBgn0031190	**CG12576**	1.939256668	2.615742044
M9NCX1	FBgn0002121	**l(2)gl**	0.569170475	2.612280699
Q9W247	FBgn0034733	**CG4752**	0.501574516	2.610290379
Q8IMF5	FBgn0002645	**Map205**	0.939098835	2.601105429
V9H0A0	FBgn0016662	**gag**	2.284747124	2.601093259
X2JCX8	FBgn0004404	**RpS14b**	0.879567146	2.599615005
Q9VJH2	FBgn0051739	**AspRS-m**	0.333879471	2.595595203
Q9W2M0	FBgn0034585	**Rbpn-5**	0.344696522	2.583216002

Protein ID	Flybase	Gene Name	Student's T-test Difference ATG5 RNAi_ctrl	-Log Student's T-test p-value ATG5 RNAi_ctrl
O62531	FBgn0024833	**AP-1mu**	0.601736546	2.571265544
M9PHM1	FBgn0259173	**corn**	2.189641476	2.570957215
A0A0B4LHE0	FBgn0261794	**kcc**	0.847440243	2.569667418
M9PBF6	FBgn0023388	**Dap160**	0.466516972	2.559330508
Q9VK59	FBgn0032454	**CG5787**	0.539141655	2.558110109
Q8IRI5	FBgn0024277	**trio**	0.849539757	2.548267896
Q9VR62	FBgn0031194	**PP2C**	0.575130939	2.543075127
E2QD65	FBgn0010412	**RpS19a**	1.086164474	2.540880464
Q9Y1A7	FBgn0002778	**mnd**	0.501338482	2.540469704
P31007	FBgn0001624	**dlg1**	0.47742033	2.531043064
Q9VV76	FBgn0036643	**Syx8**	1.389786243	2.530538568
Q9VB02	FBgn0039543	**CROT**	1.732679367	2.527338501
Q9VN02	FBgn0037231	**Vps24**	0.867722988	2.525855815
Q9W2Y5	FBgn0052685	**ZAP3**	0.605772018	2.524061031
Q9V455	FBgn0027338	**Kap-alpha3**	0.597454548	2.521586634
X2JD82	FBgn0017561	**Ork1**	0.832496166	2.507960524
Q9W3W9	FBgn0029896	**LP06294.5prime**	1.80318737	2.50455042
Q7K3D4	FBgn0034368	**zda**	0.847662926	2.501861008
Q9VPU1	FBgn0010583	**dock**	0.711231709	2.500311276
A0A0B4KHB2	FBgn0020496	**CtBP**	0.803299904	2.493220714
Q0E930	FBgn0028496	**CG30116**	2.486050129	2.492442893
Q961J5	FBgn0033778	**Balat**	0.338793278	2.491416283
Q9VXG0	FBgn0030752	**CDC50**	0.616394997	2.489299866
M9NEH6	FBgn0026428	**HDAC6**	0.502477169	2.484405379
Q9VJ58	FBgn0025608	**Faf2**	0.656838417	2.484008734
M9PC26	FBgn0263846	**CG10020**	0.64632988	2.483036247
Q9VKW8	FBgn0032240	**CG5356**	1.144766808	2.478822748
O97125	FBgn0001230	**Hsp68**	0.768706799	2.465846754
Q7KU45	FBgn0022153	**l(2)k05819**	0.567430019	2.461365102
Q9VH64	FBgn0037756	**CG8507**	0.941309452	2.459384762
Q24418	FBgn0010399	**Nmdar1**	0.584379673	2.457300788
P29746	FBgn0001090	**bnb**	1.191353798	2.455216415
M9NFR5	FBgn0022959	**yps**	0.805016518	2.449301457
E2QCK0	FBgn0050404	**Tango11**	0.796741486	2.44705357
Q867Z4	FBgn0005630	**lola**	0.757752895	2.442683549
X2JAT7	FBgn0000636	**Fas3**	0.762407303	2.4367254
Q9VWP4	FBgn0030966	**shop**	0.52799511	2.435929337
R9Q794	FBgn0064225	**RpL5**	0.808803558	2.430274917
Q9VXZ8	FBgn0027601	**pdgy**	0.888380527	2.423591671
Q9VJ30	FBgn0015772	**Nak**	0.437388897	2.419092829
E4NKG1	FBgn0042134	**Capr**	0.607332706	2.418807287
Q9VU83	FBgn0086708	**stv**	0.974002838	2.415198508
A4UZR3	FBgn0003748	**Treh**	0.273539066	2.412960112
M9PIH3	FBgn0037098	**Wnk**	0.750164986	2.405975586
Q99323	FBgn0265434	**zip**	1.12364006	2.405790788
Q8MRW1	FBgn0267976	**Tim23**	1.345817089	2.404016856
Q9VKA4	FBgn0051760	**CG31760**	0.491872787	2.39532809
Q9VMC7	FBgn0051638	**CG9545**	0.784432411	2.394765319
E9P245	FBgn0264294	**Cyt-b5**	1.192165852	2.389325871
Q9VXN3	FBgn0030699	**CG8578**	0.532962322	2.384475834
Q9VRI9	FBgn0031182	**Cyp6t1**	0.995396137	2.383762705
Q9VCC6	FBgn0039156	**CG6178**	2.580887794	2.383477385
Q8IGW0	FBgn0015582	**dare**	0.945400238	2.383368397
Q7K5K3	FBgn0039635	**Pdhb**	0.450385094	2.378604239
B5RIZ2	FBgn0038747	**RhoGAP92B**	0.715254307	2.374410266
Q9VFV9	FBgn0038145	**Droj2**	0.52657795	2.368915102
Q7K2E1	FBgn0033717	**CG8839**	0.376251698	2.36803182
O46067	FBgn0023529	**GRP170**	0.413895607	2.3640153
A0A0B4K885	FBgn0041205	**key**	1.963984966	2.358905095
Q9Y139	FBgn0027582	**CG6230**	0.673618793	2.35521567
L0MPN7	FBgn0039908	**Asator**	0.355072498	2.348882818
D2NUG3	FBgn0270928	**VAChT**	0.81664753	2.348307014
M9ZVK8	FBgn0011638	**La**	0.71808672	2.347804114

Protein ID	Flybase	Gene Name	Student's T-test Difference ATG5 RNAi_ctrl	-Log Student's T-test p-value ATG5 RNAi_ctrl
M9PID3	FBgn0037027	**HIPP1**	0.663259029	2.34644954
M9NG39	FBgn0004401	**Pep**	0.54216671	2.343437925
E2QCY9	FBgn0004575	**Syn**	0.467128277	2.342101996
M9PG43	FBgn0003744	**trc**	0.607552052	2.338669671
Q24478	FBgn0000283	**Cp190**	0.225794792	2.338530334
Q9VDI5	FBgn0038829	**CG17271**	1.439640045	2.328590603
A0A0B4JD21	FBgn0033983	**ADPS**	2.150292873	2.327544199
Q9VC10	FBgn0265190	**PIG-S**	0.731011868	2.322521833
A1Z6Q0	FBgn0265003	**koi**	0.644662857	2.320133729
M9PBW0	FBgn0035853	**UbcE2M**	1.37896204	2.316414678
Q9VEN1	FBgn0014141	**cher**	1.292876244	2.316343151
Q8SXQ1	FBgn0036857	**Aldh7A1**	0.503893375	2.313081399
A0A0B4K6Y4	FBgn0004395	**unk**	1.84774065	2.312005157
Q9W0R0	FBgn0265574	**Cdc5**	0.650714874	2.311364031
P54353	FBgn0015379	**dod**	0.814435959	2.310872809
Q9V3Q9	FBgn0260446	**GABA-B-R1**	0.355745792	2.307826393
Q95RR2	FBgn0039186	**CG5746**	0.874632835	2.303363897
Q9VQI8	FBgn0024947	**NTPase**	0.501927376	2.298013005
H0RNK5	FBgn0036889	**CG14100**	1.788659573	2.297760047
Q9VHM3	FBgn0037621	**M1BP**	0.747713566	2.297321648
P09180	FBgn0003279	**RpL4**	0.729194164	2.292139908
Q8MR50	FBgn0033229	**CG12822**	0.743739605	2.2871125
Q9VZ00	FBgn0030293	**CG1737**	0.522981167	2.287036519
O61491	FBgn0024754	**Flo1**	0.370693207	2.272835177
A0A0S0X8K7	FBgn0250874	**ttm50**	0.839575768	2.272088614
D5AEP6	FBgn0243578	**AnnX**	0.956786633	2.270945661
A1Z9L2	FBgn0033897	**Rcd1**	0.633444309	2.270649356
B7YZQ9	FBgn0035092	**Nplp1**	2.435664177	2.265996388
Q9VDE9	FBgn0038853	**RhoGAP93B**	0.625634193	2.264836694
X2JJP5	FBgn0052654	**Sec16**	0.859406948	2.262590211
O61650	FBgn0024352	**Stip1**	0.691052914	2.256183149
Q9VMI2	FBgn0031772	**CG13994**	0.794898033	2.250700933
Q7K3S2	FBgn0033426	**CG1814-RB**	0.505489826	2.248167281
A4V2K7	FBgn0000412	**D1**	0.881549835	2.243923452
A0A0B4KH51	FBgn0020510	**Abi**	0.632632732	2.240165003
Q9VUQ9	FBgn0267390	**dop**	0.771681309	2.239667947
Q9V419	FBgn0028940	**Cyp28a5**	0.613331318	2.237965303
X2JB25	FBgn0005391	**Yp2**	2.621832371	2.237452834
R9QRD4	FBgn0019952	**ORCT**	0.501724243	2.226927106
Q9V9Y0	FBgn0039844	**CG1607**	0.595664978	2.225816665
Q9VBS7	FBgn0039321	**CG10550**	0.945419312	2.220817162
Q9VMB9	FBgn0031830	**COX5B**	1.374547482	2.219830941
M9PF68	FBgn0004381	**Klp68D**	0.335768223	2.219206492
Q9W158	FBgn0035016	**CG4612**	0.566883087	2.218142134
Q9VWD9	FBgn0031057	**Ubqn**	0.578613758	2.216186278
Q95SQ6	FBgn0020372	**TM4SF**	0.985240936	2.208203664
A0A0B4K812	FBgn0015754	**Lis-1**	0.815336704	2.202449335
Q9W0D3	FBgn0035237	**CG13917**	0.715898991	2.200031086
Q9XZ19	FBgn0027524	**CG3909**	0.662519932	2.199816752
Q9V3V9	FBgn0028515	**EndoGI**	0.774765968	2.199579733
Q9VYT1	FBgn0030346	**BORCS5**	0.691252232	2.191169345
Q9VBZ5	FBgn0039261	**Ythdf**	0.550411701	2.190524218
Q8MT58	FBgn0259979	**Cndp2**	0.436166286	2.18818672
Q8IR41	FBgn0263994	**CG43737**	0.501888275	2.186843199
Q9W3X7	FBgn0029888	**ND-ASHI**	0.859421253	2.18624106
Q9VPG2	FBgn0036984	**dCat-4**	0.755281925	2.184660457
X2JD88	FBgn0264502	**CG15207**	0.36216259	2.181653351
O02195	FBgn0015834	**eIF3i**	0.566663265	2.179210539
Q7K2N0	FBgn0033309	**Lnpk**	1.62876749	2.178524585
M9PIM0	FBgn0024733	**RpL10**	1.02192831	2.177844309
B6IDW8	FBgn0053558	**CG33558**	0.848483562	2.169582679
Q9VXE8	FBgn0267384	**Ubc7**	1.231104374	2.167932362
A8JV09	FBgn0029903	**pod1**	0.463968754	2.166746563

Protein ID	Flybase	Gene Name	Student's T-test Difference ATG5 RNAi_ctrl	-Log Student's T-test p-value ATG5 RNAi_ctrl
Q8T0L4	FBgn0000289	**cg**	0.764501572	2.163330581
Q9VYV5	FBgn0030327	**FucT6**	0.974027634	2.159410055
Q09332	FBgn0014075	**Uggt**	0.362892151	2.158561273
A8DYW5	FBgn0028703	**Nhe3**	0.339920044	2.154227187
Q0KI68	FBgn0262527	**nsl1**	0.724935532	2.153695515
Q9VY16	FBgn0030581	**CG14408**	0.474517345	2.150547496
B7FNJ7	FBgn0031450	**Hrs**	0.58426857	2.145877126
M9NGQ9	FBgn0046687	**Tre1**	0.978375435	2.145574177
Q9VGW9	FBgn0261380	**mRpL37**	0.568697929	2.14533289
O44434	FBgn0022984	**qkr58E-3**	0.604176521	2.136560563
Q0E8J0	FBgn0035357	**MEP-1**	0.589755058	2.134319017
B6IDQ5	FBgn0035593	**CG4603-RA**	0.587385178	2.133576915
Q9VN95	FBgn0037305	**CG12173**	0.611679554	2.132684872
F3YDH0	FBgn0001218	**Hsc70-3**	0.623568058	2.132116634
A1ZBH2	FBgn0034419	**CG15111**	0.779883862	2.131901405
Q9W2U8	FBgn0083167	**Neb-cGP**	1.563018322	2.131526514
A8JV04	FBgn0030735	**CG3632**	0.602939606	2.128882595
Q9W0L7	FBgn0052479	**Usp10**	0.994760036	2.126996261
Q95SB2	FBgn0022344	**CG10340**	0.665497303	2.126331607
Q9VPE8	FBgn0036997	**CG5955**	0.934125423	2.122043868
Q9VZU7	FBgn0035402	**Usp5**	0.442691326	2.119471949
V5M2P5	FBgn0053547	**Rim**	0.616991997	2.117365906
Q9V400	FBgn0028427	**Ilk**	1.22875309	2.106238969
Q9VYM7	FBgn0259143	**Pripl4**	0.752135277	2.104900071
D2NUK5	FBgn0022343	**CG3760**	1.09265852	2.104143345
A0A0B4LFE1	FBgn0033951	**CG10139**	0.633892536	2.103685323
Q9W3J5	FBgn0030007	**alpha-PheRS**	0.64369154	2.101732975
Q9VAN7	FBgn0014869	**Pglym78**	0.752728462	2.101324882
E2QD00	FBgn0037374	**jagn**	0.622564316	2.096042078
Q9VWP8	FBgn0259927	**CG32543**	0.459004402	2.09231501
E1JJ98	FBgn0003464	**sol**	1.367950916	2.086484957
E1JH83	FBgn0034145	**CG5065**	0.875044346	2.085643128
E1JIJ4	FBgn0038063	**Octbeta2R**	0.778123856	2.080861131
A8Y560	FBgn0028697	**RpL15**	1.379717827	2.077897308
Q9V3L7	FBgn0027785	**NP15.6**	0.830290794	2.075431452
M9PFU0	FBgn0264308	**CG15639**	0.842409134	2.074245366
Q9VEH0	FBgn0038535	**alt**	0.698494911	2.073884781
Q960C7	FBgn0033205	**CG2064**	0.816527367	2.073797501
Q9VG23	FBgn0038088	**CG10126**	0.940752983	2.071414533
Q9VBJ3	FBgn0259146	**fid**	1.072065353	2.069899834
Q9VY92	FBgn0030519	**CG11151**	1.529267788	2.060657576
O96046	FBgn0025865	**Cortactin**	0.877203941	2.058689749
A0A0B4K8A1	FBgn0003435	**sm**	2.084187508	2.057332839
Q9VHY7	FBgn0260005	**wtrw**	0.445888042	2.054847272
Q9VL89	FBgn0025117	**und**	0.247352123	2.054636418
Q9I7T7	FBgn0035424	**Larp4B**	0.625384331	2.053936554
Q9VT00	FBgn0036004	**Jarid2**	0.848642826	2.04556758
A0A0B4LGQ8	FBgn0086605	**CG9853**	0.384590149	2.039317906
Q8IGD6	FBgn0036239	**Pop2**	1.188744545	2.034415163
A0A0B4LGY8	FBgn0263395	**hppy**	0.637890816	2.033949314
Q86DS7	FBgn0263106	**DnaJ-1**	0.446942806	2.03279544
Q9VKH0	FBgn0032361	**Cog8**	0.519154549	2.025627054
Q8IQF1	FBgn0263241	**Mocs1**	1.127472878	2.024049609
E1JHJ9	FBgn0020503	**CLIP-190**	0.380075455	2.020387587
Q9VEW6	FBgn0038418	**pad**	0.791833878	2.019995276
Q9VJZ5	FBgn0051852	**Tap42**	0.494419575	2.017127214
Q9VQK7	FBgn0031493	**Sf3b2**	0.295649052	2.011636893
Q7K519	FBgn0033272	**RagC-D**	0.406216145	2.011030448
A0A0B4LHE7	FBgn0283536	**Vha13**	1.027782917	2.008905463
Q9VC58	FBgn0039212	**Syx18**	0.681842804	2.006784155
A0A4D6K3W6	FBgn0003204	**ras**	0.413764477	2.005737966
Q9VWD0	FBgn0052528	**parvin**	1.355480194	2.004973
Q9W236	FBgn0034744	**Vps20**	1.015503407	2.000698529

Protein ID	Flybase	Gene Name	Student's T-test Difference ATG5 RNAi_ctrl	-Log Student's T-test p-value ATG5 RNAi_ctrl
X2JCS0	FBgn0029864	**CG15894**	0.691566944	2.00031089
A4UZL5	FBgn0010226	**GstS1**	0.798205376	1.994930972
A0A0B4LI86	FBgn0262955	**RpII140**	0.255694389	1.993088997
P32748	FBgn0000447	**Dhod**	0.819711685	1.992885149
M9PJK5	FBgn0267001	**Ten-a**	0.325946331	1.981993936
Q9VVW3	FBgn0036843	**Sfxn2**	0.45031929	1.973695807
Q7KTU3	FBgn0037188	**CG7369**	0.715139389	1.967917882
Q8SXS0	FBgn0260467	**CG7071**	1.078880787	1.966948203
Q9V405	FBgn0028686	**Rpt3**	0.367323399	1.964251565
Q9VCA0	FBgn0039178	**CG6356**	0.756425381	1.961749907
B5RJ70	FBgn0086913	**Rab26**	0.600113869	1.957760366
A0A0B4KI59	FBgn0039730	**CG7903**	0.719232082	1.948861949
A8DZ29	FBgn0023213	**eIF4G1**	0.545462132	1.948620419
Q9VXP3	FBgn0030692	**mRpS30**	0.716902733	1.947002105
Q9VN25	FBgn0037249	**eIF3a**	0.337666512	1.942765078
A1Z934	FBgn0033761	**CG8778**	0.611711979	1.940732878
Q9XYZ9	FBgn0027590	**GstE12**	0.577888489	1.939647424
A0A0B4K5Z4	FBgn0263346	**smash**	1.216524124	1.932995979
M9PBZ9	FBgn0015218	**eIF4E1**	0.580943584	1.930878361
Q9VNR6	FBgn0010830	**l(3)04053**	0.70059967	1.9304109
M9MRJ4	FBgn0261836	**Msp300**	1.175998688	1.926941439
A0A0B4LH72	FBgn0038387	**blp**	0.974612236	1.926611313
Q9W255	FBgn0022986	**qkr58E-1**	0.567928314	1.920557947
Q9W2M4	FBgn0034583	**CG10527**	0.513288498	1.92032282
A0A0B4KFL2	FBgn0033844	**bbc**	1.031865597	1.912737478
M9PHL6	FBgn0014133	**bif**	0.50838089	1.911459818
T2FFB7	FBgn0027094	**Aats-ala**	0.342301846	1.904721408
X2J935	FBgn0032725	**Nedd8**	0.629517555	1.901673656
P20240	FBgn0266420	**Ote**	0.738916397	1.895296063
Q9W1I8	FBgn0034913	**Snap29**	0.589827538	1.894275713
H0RN81	FBgn0013954	**FK506-bp2**	0.747964859	1.89303944
Q9W1I7	FBgn0034914	**CG5554**	0.60035038	1.885528143
Q9W022	FBgn0035334	**CG8993**	1.166200638	1.880161318
O77410	FBgn0025582	**eIF3e**	0.575661659	1.877870054
H0RND2	FBgn0035944	**CG5021**	1.300353527	1.876496484
Q9U6R9	FBgn0028552	**gammaSnap1**	0.591356754	1.873763622
M9PCV9	FBgn0036740	**Vps60**	0.547489643	1.873528152
X2JGG6	FBgn0001225	**Hsp26**	1.039604664	1.872262948
Q8T410	FBgn0015622	**Cnx99A**	0.480555534	1.871076778
Q9VFU7	FBgn0038156	**side-IV**	0.755821705	1.869352757
E0R905	FBgn0265276	**l(3)neo38**	1.569259167	1.864891316
Q9VF39	FBgn0038360	**CG9590**	0.454161644	1.86250128
M9PG62	FBgn0261396	**Rpn3**	0.432284355	1.861283168
M9PC95	FBgn0259111	**Ndae1**	1.057449341	1.859416919
A0A0B4K7R1	FBgn0039172	**Spase22-23**	2.425182343	1.85891333
Q8SX35	FBgn0052850	**RNF-11**	0.81262064	1.852691521
A0A0B4KFB8	FBgn0261641	**CG31367**	0.619298935	1.851877477
Q9Y134	FBgn0001986	**Mtr4**	0.817131519	1.850253205
Q9VEH2	FBgn0038533	**CG7523**	0.87869072	1.849607854
A4V3W8	FBgn0040384	**CG2716**	1.075758457	1.846788157
Q9VQM2	FBgn0031505	**ND-B14.5B**	0.692460537	1.842114922
M9PDX2	FBgn0031306	**CG4577**	1.219779015	1.838339953
O16043	FBgn0022893	**Df31**	1.067259312	1.836700205
Q9VDE6	FBgn0038856	**Sec15**	0.43406868	1.836666255
P53624	FBgn0259170	**alpha-Man-Ia**	0.540560246	1.834540342
Q9VFP0	FBgn0038195	**CG3061**	0.655666828	1.834152108
C9DA46	FBgn0034381	**CG15088**	0.441271782	1.831381367
Q9W0H3	FBgn0035206	**CG9186**	0.898369789	1.831309519
Q9W0M4	FBgn0035165	**CG13887**	1.768850327	1.830468248
Q7KKI0	FBgn0010621	**CCT5**	0.408788204	1.829860243
Q9VQR2	FBgn0021967	**ND-PDSW**	0.351341248	1.824233464
Q9W003	FBgn0010905	**Spn**	0.559258938	1.822470572
P22979	FBgn0001229	**Hsp67Bc**	1.161046028	1.821731414

Protein ID	Flybase	Gene Name	Student's T-test Difference ATG5 RNAi_ctrl	-Log Student's T-test p-value ATG5 RNAi_ctrl
Q7KMS3	FBgn0024196	**robl**	0.90882206	1.821656793
M9PD94	FBgn0020309	**crol**	1.463421345	1.81687636
Q9VI04	FBgn0037513	**pyd3**	0.683083534	1.812171851
Q9VRP3	FBgn0035631	**Txl**	0.807836533	1.811020697
Q9VB22	FBgn0040080	**pins**	0.332633495	1.809950395
B6IDR5	FBgn0035471	**Sc2**	1.251073837	1.808463362
Q9VZN2	FBgn0052264	**CG10857**	0.824276447	1.807619107
M9PAY9	FBgn0031257	**CG4133**	0.695230961	1.805248261
Q7JZN0	FBgn0010638	**Sec61beta**	1.014738083	1.804746996
Q9VXF9	FBgn0030753	**rngo**	0.725404739	1.80356264
Q9W3M4	FBgn0027280	**l(1)G0193**	0.755786896	1.799642879
P17336	FBgn0000261	**Cat**	0.517500877	1.796959279
A0A0B4KFD5	FBgn0025720	**Ate1**	1.006466389	1.794018185
D0Z769	FBgn0003277	**RpII215**	0.29711771	1.789741495
Q9V4C4	FBgn0039900	**Syt7**	0.357459068	1.786533408
Q9VC99	FBgn0263398	**Uck**	0.591138363	1.786429701
Q7KLX3	FBgn0021795	**Tapdelta**	0.550311565	1.782538321
A8JNN5	FBgn0035879	**GAPcenA**	0.654286385	1.781634633
Q9VZY9	FBgn0027082	**ProRS-m**	0.666903973	1.78139746
Q7KTP7	FBgn0264743	**CG5822**	0.626621723	1.778949999
M9PBM2	FBgn0004875	**enc**	0.799704552	1.777194209
Q9VCE9	FBgn0039137	**CG13604**	0.395349979	1.776354901
Q9VXV4	FBgn0030638	**CG11655**	1.180544853	1.773751146
Q9W1G7	FBgn0015268	**Nap1**	0.461096764	1.773493898
Q9VQX6	FBgn0031590	**CG3702**	0.8254776	1.773227329
Q8SYJ2	FBgn0052230	**ND-MLRQ**	0.845507622	1.771019361
E1JI22	FBgn0024814	**Clc**	0.722640991	1.769780472
A0A0B4LIJ0	FBgn0015282	**Rpt2**	0.401573181	1.765774957
A0A0B4KHR8	FBgn0043884	**mask**	0.460790157	1.764963326
T2FGC6	FBgn0039827	**CG1544**	0.692691803	1.764276586
M9PIC3	FBgn0037010	**Pss**	1.01265955	1.757365725
Q0E8X8	FBgn0040660	**CG13551**	2.200327396	1.755560156
Q95U32	FBgn0051344	**Rpb7**	0.519425392	1.753592582
Q0KHZ4	FBgn0083969	**CG34133**	0.527424812	1.75301273
Q8SYD9	FBgn0034433	**EndoB**	0.408331871	1.75120656

Appendix Table S2 – related to Fig. 5+6

All significantly downregulated proteins upon MB-specific *Atg5* knockdown

Protein ID	Flybase	Gene Name	Student's T-test Difference ATG5 RNAi_ctrl	-Log Student's T-test p-value ATG5 RNAi_ctrl
Q5BIC0	FBgn0026439	**Eaat1**	-1.247505665	4.659274569
P27864	FBgn0004584	**Rrp1**	-0.567914963	4.430452075
Q9W441	FBgn0029837	**Tsp5D**	-2.02828455	4.263844286
A0A0K0K2F3	FBgn0262952	**ND4**	-1.587227821	4.247844187
Q9VJ14	FBgn0086442	**mib2**	-1.990968704	4.236590545
Q8T3K1	FBgn0039508	**CG3368**	-1.58000946	4.19234362
Q9VMR6	FBgn0031703	**CG12512**	-0.706595898	4.021937091
P53034	FBgn0260985	**RfC4**	-2.592122555	3.904658868
Q8SX37	FBgn0030670	**Pis**	-0.994651794	3.846582873
Q8T0U6	FBgn0000711	**flw**	-0.734861374	3.843885702
Q9VEX2	FBgn0250823	**gish**	-2.604410172	3.764658161
Q9VI13	FBgn0267698	**Pak**	-0.629755497	3.711834207
Q9VFB7	FBgn0038303	**SIDL**	-0.436401844	3.659612867
M9PEA7	FBgn0035677	**CG13293**	-0.852367401	3.528129472
Q9VF17	FBgn0051183	**CG11846**	-0.751546383	3.469857442
F0JAN9	FBgn0036257	**RhoGAP68F**	-1.319040775	3.453029706
Q9VCF4	FBgn0039132	**AP-1sigma**	-1.151524544	3.442318436
Q967S7	FBgn0044442	**gag**	-1.089314938	3.416688077
Q9VSK2	FBgn0020224	**Cbl**	-0.647787094	3.390520328
Q8IP22	FBgn0261999	**Ca-Ma2d**	-0.58769083	3.38784495
M9PBU9	FBgn0031414	**eys**	-2.670945644	3.373617598
Q9VIU8	FBgn0032798	**CG10132**	-0.851373672	3.356271814
Q86P01	FBgn0024182	**waw**	-1.13344717	3.352076433
Q9XYZ5	FBgn0260962	**pic**	-0.160964966	3.32771686
A0A0B4LGA1	FBgn0034902	**CG5532**	-1.981283665	3.268703821
A4V4I0	FBgn0001105	**Gbeta13F**	-1.259864807	3.114774063
M9NES0	FBgn0000120	**Arr1**	-1.826866627	3.100356232
Q9W3R7	FBgn0029943	**Atg5**	-0.550779343	3.09778862
E1JGX3	FBgn0026401	**Nipped-B**	-0.831085682	3.045760182
E8NHB1	FBgn0011836	**Taf2**	-0.833092213	3.010712155
Q9VTW1	FBgn0036272	**Sms**	-0.515211582	2.996665794
Q9VEC5	FBgn0261532	**cdm**	-1.753203869	2.952048145
P21521	FBgn0004242	**Syt1**	-0.721171379	2.930820829
Q8IQV9	FBgn0031078	**Nup205**	-0.758240223	2.917044839
A8JQT1	FBgn0085386	**CG9783**	-0.678024769	2.901060547
Q5BIA9	FBgn0033924	**CG8613**	-1.099391937	2.884304755
E1JIV7	FBgn0003429	**slo**	-0.692589283	2.88195284
A0A0B4LH50	FBgn0000046	**Act87E**	-0.658251762	2.868406468
Q9VVX5	FBgn0026630	**nes**	-0.855999947	2.860734213
Q9V463	FBgn0021761	**Nup154**	-0.720492363	2.857560376
A1ZAN2	FBgn0034180	**Ehbp1**	-0.975770473	2.855098884
Q9W1F4	FBgn0034939	**thoc5**	-0.974275589	2.835545417
Q24253	FBgn0010380	**AP-1-2beta**	-1.138159275	2.823150595
X2J516	FBgn0000053	**Gart**	-1.48904705	2.820591985
Q24160	FBgn0015737	**Hmu**	-1.437750816	2.805018387
E8NHB5	FBgn0000038	**nAcRbeta-64B**	-0.695033073	2.762121778
A8JR71	FBgn0013759	**CASK**	-0.684382439	2.736437184
Q9VYT3	FBgn0030344	**Nrd1**	-0.286151409	2.7348801
A0A0B4KFE5	FBgn0010222	**Nmdmc**	-0.668919086	2.718900046
Q9VVM8	FBgn0036772	**CG5290**	-1.05629921	2.705810615
P55162	FBgn0011771	**Hem**	-0.660661697	2.681256859
U3PV94	FBgn0261787	**brun**	-0.514210701	2.670596852
Q9VNH5	FBgn0037372	**CG2091**	-0.796307087	2.656647483
E2QD75	FBgn0022942	**Cbp80**	-1.094027519	2.649171289
Q8MRI4	FBgn0030336	**CG1578**	-1.044643402	2.646895998
Q9NHV8	FBgn0040493	**grsm**	-0.416300297	2.645497985
Q9U787	FBgn0028408	**Drep2**	-0.563411236	2.642475076
Q9VAK1	FBgn0086361	**alph**	-0.601245403	2.634017568

Protein ID	Flybase	Gene Name	Student's T-test Difference ATG5 RNAi_ctrl	-Log Student's T-test p-value ATG5 RNAi_ctrl
Q8SX39	FBgn0033958	**jef**	-0.440877914	2.631644361
Q9V429	FBgn0040070	**Trx-2**	-2.380078316	2.619776461
Q9VM10	FBgn0025697	**santa-maria**	-0.901993752	2.615365915
Q8IMQ8	FBgn0039464	**CG6330**	-1.210037708	2.612729477
Q9VB69	FBgn0029155	**Men-b**	-0.558570862	2.601484433
A1Z9E3	FBgn0024556	**mEFTu1**	-1.06567812	2.566179505
Q9V3Y4	FBgn0027786	**Mtch**	-0.797914982	2.554027216
M9NGR8	FBgn0085387	**shakB**	-1.006640434	2.549061965
A1YK86	FBgn0039004	**Nup133**	-0.741430759	2.541561192
Q03445	FBgn0004619	**GluRIA**	-2.264136314	2.530845195
Q7PLS8	FBgn0262117	**IntS3**	-0.787387371	2.509293735
Q9W1X7	FBgn0034795	**MED23**	-1.019356251	2.507576314
A0A0B4LIA3	FBgn0010379	**Akt1**	-1.650306225	2.500552255
A0A0B4LH53	FBgn0266464	**Nsf2**	-0.77220726	2.486788346
Q9VP27	FBgn0264561	**Glg1**	-1.522875309	2.469828469
Q9XZU1	FBgn0022213	**Cse1**	-0.69940424	2.45828017
Q9VIM1	FBgn0263355	**CG11017**	-0.644228935	2.444213347
M9MQH9	FBgn0260439	**Pp2A-29B**	-0.440115452	2.438934202
M9PFZ6	FBgn0000045	**Act79B**	-1.237658978	2.404278477
Q9VWB0	FBgn0052226	**Pex23**	-0.460317612	2.394464263
Q24241	FBgn0011747	**Ank**	-0.264620781	2.379480119
B3DMZ9	FBgn0028667	**CG32090**	-2.665279388	2.371990422
Q7JWW6	FBgn0026602	**Adk3**	-0.573194981	2.366622844
Q9VU39	FBgn0036337	**Adk2**	-0.597268105	2.365727673
Q7K1C5	FBgn0034269	**Hyccin**	-0.659865379	2.349449961
A4V3W2	FBgn0003371	**sgg**	-1.623731613	2.334329994
A1YK08	FBgn0034310	**Nup75**	-0.794615746	2.3268552
Q9NHP1	FBgn0260799	**p120ctn**	-0.952807903	2.324366758
A0A0C4FEI4	FBgn0037228	**CG1092**	-0.682971478	2.316194634
Q9W4X4	FBgn0267350	**Pi4KIIIalpha**	-0.381973267	2.314023633
P40421	FBgn0265959	**rdgC**	-0.614055157	2.292467876
A0A0B4K664	FBgn0027948	**msps**	-0.560742855	2.277792271
M9MRG0	FBgn0028704	**Nckx30C**	-0.564293861	2.273057493
A4V0U4	FBgn0032721	**CG10602**	-1.300421715	2.261745351
X2JB48	FBgn0003360	**sesB**	-0.334412575	2.259784432
A0A0B4KI25	FBgn0066101	**LpR1**	-0.472026825	2.243762222
Q9VE62	FBgn0023083	**fray**	-0.811131477	2.233470074
A1Z945	FBgn0033766	**Nup188**	-0.600279331	2.230173728
Q9VK44	FBgn0032467	**Ube4B**	-0.219268322	2.226250666
A0A0B4KGW0	FBgn0039140	**Miro**	-1.117631435	2.202312071
A0A0B4LIE3	FBgn0262869	**Gfrl**	-0.318349361	2.20055696
Q9V492	FBgn0039930	**CG11077**	-1.36037302	2.200464915
Q7K511	FBgn0023507	**D2hgdh**	-1.03046751	2.198565709
Q94883	FBgn0015664	**Dref**	-0.500582695	2.165806431
A1Z942	FBgn0261625	**GLS**	-0.768095493	2.163764563
A8JNU6	FBgn0010352	**Nc73EF**	-0.443123817	2.162581596
Q9W0H2	FBgn0035207	**Herc4**	-0.396798134	2.160712368
A0A0B4KF57	FBgn0037218	**aux**	-0.222950935	2.152560554
O44116	FBgn0022960	**vimar**	-0.363367081	2.144412772
A0A0B4KHS9	FBgn0003134	**Pp1alpha-96A**	-1.100615978	2.143491008
Q7K0H4	FBgn0261041	**stj**	-0.400728703	2.139285567
A8JMD5	FBgn0085385	**bma**	-0.512637615	2.134061986
Q8T6L6	FBgn0039157	**Myo95E**	-0.39434576	2.120891507
Q9W196	FBgn0034989	**CG3356**	-0.585351944	2.120617847
X2JDI1	FBgn0004177	**mts**	-0.724621296	2.115730937
A0A0B4LFX0	FBgn0000578	**ena**	-1.293921471	2.108414447
Q9VPY7	FBgn0031320	**CG5126**	-0.363037586	2.101176862
Q9VFE7	FBgn0038274	**Nup93-2**	-0.919201374	2.099613991
P91938	FBgn0020653	**Trxr-1**	-0.589828491	2.07510714
Q9VXH7	FBgn0030740	**CG9917**	-2.244744778	2.063294471
Q59E09	FBgn0012034	**AcCoAS**	-1.027160168	2.057836085
Q9VPW9	FBgn0031304	**TBC1D23**	-0.838861465	2.055472266
B3STG5	FBgn0002774	**MLE**	-0.360711575	2.046459547

Protein ID	Flybase	Gene Name	Student's T-test Difference ATG5 RNAi_ctrl	-Log Student's T-test p-value ATG5 RNAi_ctrl
A1Z783	FBgn0033244	**CG8726**	-1.058238029	2.035536227
Q9VW22	FBgn0260655	**l(3)76BDm**	-0.420752048	2.035286837
M9PDK5	FBgn0027779	**VhaSFD**	-0.87620163	2.0338341
Q9VC93	FBgn0039183	**Dis3**	-0.540194511	2.024545698
P25991	FBgn0003559	**su(f)**	-0.810555935	2.018510433
M9NF25	FBgn0263111	**cac**	-0.863296509	2.010708299
A0JL24	FBgn0036134	**FoxK**	-0.742759705	1.981464999
Q9VN86	FBgn0037298	**Sccpdh1**	-0.659210205	1.972334289
E8NH00	FBgn0027554	**CG8042**	-1.970517635	1.964907263
Q9W299	FBgn0034688	**CG11474**	-0.438964367	1.964498919
Q9VAH7	FBgn0260990	**yata**	-0.327300072	1.963113394
Q9W1R5	FBgn0003977	**vir**	-0.724261761	1.952902372
P23625	FBgn0004435	**Galphaq**	-0.408500671	1.945485332
X2JCY2	FBgn0266452	**CTPsyn**	-0.520254612	1.942734205
A1ZBD6	FBgn0034389	**Mctp**	-0.759168148	1.94200176
A1Z6H7	FBgn0266580	**Gp210**	-0.209863663	1.9366225
Q9VL71	FBgn0024285	**Srp54**	-0.863901615	1.927949105
M9NDM4	FBgn0040066	**wds**	-0.870414734	1.926976619
H5V8C9	FBgn0260237	**CG42511**	-1.188139439	1.920636753
Q59E36	FBgn0261573	**CoRest**	-0.949168682	1.917424717
X2J9F3	FBgn0262647	**Nup160**	-0.720887184	1.915750167
Q9VBL2	FBgn0039380	**CG5890**	-2.055403233	1.907342004
F0JAM7	FBgn0243486	**rdo**	-0.69709444	1.896643317
Q9W542	FBgn0023509	**mip130**	-0.340179443	1.889066615
C9QP42	FBgn0001942	**eIF4A**	-0.566495895	1.881729038
Q9NGV3	FBgn0035710	**SP1173**	-0.850805283	1.880680631
Q9VC89	FBgn0039187	**CG6454**	-0.434412479	1.866892293
A0A0B4KI69	FBgn0261988	**Gprk2**	-0.511816978	1.857406514
P25171	FBgn0002638	**Rcc1**	-0.741811752	1.848802055
Q4V6M1	FBgn0051713	**Datp**	-0.812245369	1.842345916
Q9VM33	FBgn0263133	**mEFG1**	-0.666275501	1.841598639
Q9VUH9	FBgn0036451	**Helz**	-0.597185612	1.84084139
B7Z0L0	FBgn0262742	**Fas1**	-3.47694397	1.839438271
Q7K569	FBgn0022160	**Gpo1**	-0.686201096	1.834471446
Q7KUB0	FBgn0001248	**Idh**	-0.476302624	1.832610802
Q24560	FBgn0003887	**betaTub56D**	-0.467756271	1.828112185
Q9VMI9	FBgn0025742	**mtm**	-1.18538332	1.824782873
Q9VNF5	FBgn0037356	**anon-WO0118547.367**	-0.879839897	1.818402021
E1JGU6	FBgn0266377	**Pde8**	-0.74241066	1.814293792
Q9W259	FBgn0034723	**CG13506**	-1.344730854	1.812545131
Q8MRR5	FBgn0034069	**CG8401**	-0.765070915	1.81210293
B7YZT0	FBgn0050361	**mtt**	-0.444045544	1.811698732
A0A0B4JDG2	FBgn0261550	**CG15684**	-0.281820297	1.809513968
Q9VXR5	FBgn0030672	**CG9281**	-0.683750629	1.805267129
A0A0B4K7K6	FBgn0039417	**CG6073**	-0.669909477	1.802354111
Q9VBT7	FBgn0039311	**CG10513**	-0.345255375	1.795749023
A0A0S0WNN6	FBgn0020497	**emb**	-0.827349663	1.772368255
A0A126GUU0	FBgn0260444	**Not10**	-1.25229311	1.771092858
A0A0B4KFT3	FBgn0003091	**Pkc53E**	-0.71807766	1.764520478
Q9NF74	FBgn0040348	**EG:BACR7A4.15**	-0.33954525	1.762076575
C0HL67	FBgn0004828	**His3.3B**	-0.612571239	1.756500109
