# Supplementary material for: Local autophagy impairment triggers brain-wide presynaptic remodeling and resilience
Source: EMBO J. 2026 Jul 8;45(17):6054–85. doi: 10.1038/s44318-026-00847-4 (PMC13534471; doi:10.1038/s44318-026-00847-4)
Supplement: Supplementary file 3 [file 44318_2026_847_MOESM2_ESM.pdf]

THE EMBO JOURNAL

Local autophagy impairment triggers brain-wide presynaptic remodeling and resilience

David Toppe, Sheng Huang, Janine Lützkendorf, Pin-Lian Jiang, Raquel Suárez-Grimalt, Alexander Neumann, Zhiying Zhao, Péter Lőrincz, Lisa Scheunemann, Gábor Juhász, Fan Liu, Marta Maglione, and Stephan Sigrist

Corresponding author(s): Stephan Sigrist (stephan.sigrist@fu-berlin.de)

Review Timeline:

Submission Date:	6th Aug 25
Editorial Decision:	28th Sep 25
Revision Received:	4th Apr 26
Editorial Decision:	6th May 26
Revision Received:	19th May 26
Accepted:	28th May 26

Editor: Ioannis Papaioannou

Transaction Report:

(Note: With the exception of the correction of typographical or spelling errors that could be a source of ambiguity, letters and reports are not edited. Depending on transfer agreements, referee reports obtained elsewhere may or may not be included in this compilation. Referee reports are anonymous unless the Referee chooses to sign their reports.)

Dear Stephan,

Thank you again for the submission of your manuscript EMBOJ-2025-122093 for consideration by The EMBO Journal, and for your patience during peer review. Your manuscript has now been seen by three experts in the field, and we have received the full set of their reports, which are included below.

I am very pleased to say that the referees recognize that this is an important topic and indicate interest in your findings, pointing out that the study is well-performed and the results are clearly and appropriately presented and interpreted. They acknowledge that this work provides new insights into the importance of autophagy in Drosophila mushroom body neurons with links to brain homeostasis and ageing. They also raise a number of conceptual and technical concerns, however, and they make a number of suggestions that would increase the solidity of the work, provide more mechanistic insight, and improve the clarity of presentation.

In light of the largely positive referees' comments and recommendations, I would like to invite you to submit a revised version of your manuscript taking the referees' suggestions on board, along with a detailed point-by-point response addressing all referees' comments. Please note that it is The EMBO Journal policy to allow only a single round of major revision, and acceptance of your manuscript will therefore depend on the completeness of your responses in this revised version.

Please let me know if you have any questions or comments that you would like to discuss with me; it would be useful to discuss your revision plan aready during the initial phase of your revisions. You are very welcome to share with me a draft point-by-point response letter/revision plan explaining if there are any points you do not agree with or cannot address, or alternatively we could arrange a video call, if you prefer.

We generally allow three months as standard revision time (December 27, 2025). As a matter of policy, competing manuscripts published during this period will not negatively impact our assessment of the conceptual advance presented by your study. However, we request that you contact us as soon as possible upon publication of any related work, to discuss how to proceed. Should you foresee a problem in meeting this three-month deadline, please let us know in advance and we will be able to grant an extension.

Thank you for the opportunity to consider your work for publication in The EMBO Journal. I look forward to your revision.

Best regards,

Ioannis

Ioannis Papaioannou, PhD
Editor, The EMBO Journal
i.papaioannou@embojournal.org

*****
Instructions for preparing your revised manuscript
*****

1. When you are ready to submit the revision, please upload:

- A Word file of the manuscript text (including legends of main Figures, EV Figures and Tables). Please make sure that changes are highlighted (or "tracked") to be clearly visible.

- Individual production-quality figure files (one file per figure). When assembling your figures, please refer to our figure preparation guidelines in order to ensure proper formatting and readability in print as well as on screen:
https://bit.ly/EMBOPressFigurePreparationGuideline
If the data shown in a figure are obtained from n {less than or equal to} 2, please use scatter plots showing the individual data points.
Please also include scale bars in all microscopy images.
The following points must be specified in each figure legend:
i. the name of the statistical test used to generate error bars and P values
ii. the number (n) of independent experiments (please specify technical or biological replicates) underlying each data point
(discussion of statistical methodology can be reported in the Materials and Methods section, but figure legends should contain a basic description of n, P, and the test applied)

iii. the nature of the bars and error bars (s.d., s.e.m.).
See also guidelines for figure legends: https://www.embopress.org/page/journal/14602075/authorguide#figureformat

- A point-by-point response to the referees' comments, with a detailed description of the changes made (as a word file). All referees' concerns must be fully addressed and their suggestions taken on board. When preparing your letter of response to the referees' comments, please bear in mind that this will form part of the Review Process File and will therefore be available online to the community. Please note that you have the possibility to opt out of the transparent process at any stage prior to publication by letting the editorial office know (contact@embojournal.org); if you do opt out, the Review Process File link will point to the following statement: "No Review Process File is available with this article, as the authors have chosen not to make the review process public in this case.". For more details on our Transparent Editorial Process, please visit our website: https://www.embopress.org/page/journal/14602075/authorguide#transparentprocess

- Expanded View (EV) files (replacing Supplementary Information) that are collapsible/expandable online. A maximum of 5 EV Figures can be typeset. EV Figures should be cited as "Figure EV1, Figure EV2" etc. in the text, and their respective legends should be included in the manuscript file after the legends of regular figures. See detailed instructions regarding Expanded View files here:
https://www.embopress.org/page/journal/14602075/authorguide#expandedview

- For the figures that you do NOT wish to display as Expanded View figures, they should be bundled together with their legends in a single PDF file called "Appendix", which should start with a short Table of Contents (including page numbers). Appendix figures should be referred to in the main text as: "Appendix Figure S1, Appendix Figure S2" etc. Please see detailed instructions here: https://www.embopress.org/page/journal/14602075/authorguide#expandedview

- A complete author checklist, which you can download from our author guidelines (https://www.embopress.org/page/journal/14602075/authorguide). Please note that the checklist will also be part of the Review Process File.

2. Please note that no statistics should be calculated and shown in Figures if n=2. Please also note that each p value should be reported as an exact value.

3. Before submitting your revision, primary datasets (and computer code, where appropriate) produced in this study need to be deposited in appropriate public databases (see https://www.embopress.org/page/journal/14602075/authorguide#dataavailability). In particular, all mass spectrometry and RNA sequencing data generated in your study must be deposited in an appropriate repository. The accession numbers, database, and the specific URLs (links) should be listed in a formal "Data availability" section (placed after Methods), following the example below:

"The RNA-seq datasets produced in this study are available in the following database:
Gene Expression Omnibus GSE46843 (https://www.ncbi.nlm.nih.gov/geo/query/acc.cgi?acc=GSE46843)"

*** All links should resolve to a page where the data can be accessed. ***

*** Please remember to provide in the Data availability section of your revised manuscript reviewer passwords if the datasets are not yet public. ***

*** The Data Availability Section is restricted to new primary data that are part of this study. In case you have no data that require deposition in a public database, please state so instead of referring to the database: "Our study includes no data deposited in public repositories." under the heading "Data availability". ***

4. The materials and methods need to be described in the manuscript using our structured methods format, which is now required for all research articles. According to this format, the Methods section includes a single "Reagents and Tools Table" - listing key reagents, experimental models, software and relevant equipment including their sources and relevant identifiers- followed by a "Methods and Protocols" section describing the methods. Please download and fill our Reagents and Tools Table template (.docx), which you can find in our author guide:
https://www.embopress.org/page/journal/14602075/authorguide#structuredmethods. When submitting your revised manuscript, please do not include the Reagents and Tools Table in the Methods section of the manuscript but instead upload it as a separate file choosing the file type "Reagent Table".

5. Please check that the title and the abstract of the manuscript are brief, yet explicit, even to non-specialists. The length of the title should not exceed 100 characters, and the abstract should be a single paragraph not exceeding 175 words.

6. Please also note our reference format: https://www.embopress.org/page/journal/14602075/authorguide#referencesformat.

7. At EMBO Press we ask authors to provide source data for the main manuscript figures. You will receive a separate email with instructions for providing source data with your revised manuscript, including how to upload and organize the files.

8. Please remember: digital image enhancement is acceptable practice, as long as it accurately represents the original data and conforms to community standards. If a figure has been subjected to significant electronic manipulation, this must be noted in the figure legend or in the "Materials and Methods" section. The editors reserve the right to request original versions of figures and the original images that were used to assemble the figure.

9. Our journal encourages inclusion of data citations in the reference list to directly cite datasets that were obtained from public databases. Data citations in the article text are distinct from normal bibliographical citations and should directly link to the database records from which the data can be accessed. In the main text, data citations are formatted as follows: "Data ref: Smith et al, 2001" or "Data ref: NCBI Sequence Read Archive PRJNA342805, 2017". In the Reference list, data citations must be labeled with "[DATASET]". A data reference must provide the database name, accession number/identifiers, and a resolvable link to the landing page from which the data can be accessed at the end of the reference. Further instructions are available at: https://www.embopress.org/page/journal/14602075/authorguide#referencesformat.

10. We request authors to consider both actual and perceived competing interests. Please review our policy (https://www.embopress.org/page/journal/14602075/authorguide#conflictsofinterest) and update your competing interests statement if necessary. Please name this section 'Disclosure and competing interests statement' and place it after the Acknowledgements section.

11. Please note that all corresponding authors are required to provide an ORCID ID upon submission of a revised manuscript (https://orcid.org/). Please find instructions on how to link your ORCID ID to your account in our manuscript tracking system in our Author guidelines
(https://www.embopress.org/page/journal/14602075/authorguide#authorshipguidelines).

12. We use CRediT to specify the contributions of each author in the journal submission system. CRediT replaces the author contribution section, which should be removed from the manuscript. Please use the free text box to provide more detailed descriptions. See also guide to authors:
https://www.embopress.org/page/journal/14602075/authorguide#authorshipguidelines.

13. Further information is available in our Guide For Authors: https://www.embopress.org/page/journal/14602075/authorguide

14. We would also welcome the submission of cover suggestions or motifs to be used by our Graphics Illustrator in designing a cover.

15. Please use the link below to submit your revision:
https://emboj.msubmit.net/cgi-bin/main.plex

Referee #1:

In this manuscript the authors follow up on their previous work showing that inhibition of autophagy in Drosophila mushroom body (MB) neurons causes brain-wide increases in the active zone protein Brp. They use RNAi to inhibit autophagy in MB neurons through knockdown of Atg5 and analyse the effects on markers of synaptic plasticity, sleep and lifespan. They show a temporal requirement for Atg5 in MB neurons during early ageing, which correlates with perturbed sleep and increases in lifespan. Importantly, MB-specific expression of Atg7 in Atg7 mutant flies is sufficient to restore normal sleep. The increased lifespan caused by MB-specific loss of Atg5, although small, required Brp expression since a heterozygous Brp mutation abrogates the lifespan extension. Proteomic analysis of brain tissue from MB neuron-specific Atg5 knockdown flies showed pronounced changes in endoplasmic reticulum and chaperone proteins. Increases in specific active zone proteins were also observed while changes in general synaptic vesicle components were much less apparent. Finally, MB-specific Atg5 knockdown caused an intriguing accumulation of Ref(2)P/P62 aggregates throughout the brain.

This is a well-executed study that provides further insight into the importance of autophagy in MB neurons in relation to maintenance of brain homeostasis and brain ageing. The data are clear and appropriately interpreted. The proteomic data provide general insight into the brain-wide molecular changes caused by MB-specific Atg5 knockdown, however they are somewhat preliminary, descriptive and lack detailed follow-up regarding the non-cell autonomous causes of these proteomic changes. Similarly, the brain wide accumulation of Ref(2)P/P62 aggregates is an intriguing result but the mechanisms that enable MB neurons to effect such widespread non cell autonomous molecular changes are not clear. Extending the study to include more insight into the mechanisms involved would increase the significance.

I have the following comments:
1. It is shown in Figure 2E,F that MB specific expression of Atg7 rescues the sleep phenotypes caused by an Atg7 null mutation. Do Atg7 mutants have reduced lifespan and if so does MB specific Atg7 expression rescue the lifespan phenotype of the mutant flies?

2. Please include more details of how proteomic data analyses were performed in the main text and figure legends. How old were the flies used for the proteomics? Please state the statistical cutoff used and whether a fold change cutoff was used. In the figure legends for the volcano plots describe what the lighter dots represent. Presumably these are below the statistical cutoff? The fold changes of the presynaptic AZ proteins labelled in Figure 4A are very small. It would be interesting to include volcano plots highlighting the proteins with the greatest fold change/p value. Presumably many of these are endoplasmic reticulum and chaperone proteins. Tables with details of the proteins and their fold change/p value would also be helpful. Please include the details of the significantly upregulated and downregulated proteins in the proteomic analysis as supplemental tables and upload the raw proteomic data to a publicly accessible database.

3. Is there increased ubiquitination of any of the proteins whose levels are altered in MB-specific Atg5 knockdown flies?

4. There are clear parallels between this study and recent work showing non cell autonomous integrated stress response activation due to inhibition of photoreceptor neuron activity in Drosophila (PMID: 39747204), which also observed non cell autonomous accumulation of Ref(2)p. Apart from the increased expression of ER stress response proteins that is reported from the proteomics data, can the authors test whether there is direct evidence of ISR activation cell autonomously or throughout the brain with MB-specific Atg5 knockdown? The authors should also discuss the links between the current study and the findings in PMID: 39747204.

Referee #2:

Toppe et al., in this report build on the work of the Sigrist lab identifying an accumulation of pre-synaptic active zone (AZ) proteins increasing sleep need in aging flies termed 'PreScale'. This accumulation appears to be a mechanism preserving synaptic function and a possible homeostasis adjustment supporting aging. Critically it can be activated in the mushroom body (MB), a structure critical to learning and memory and sleep need, and activation in the MB can trigger a pan-CNS 'PreScale' (PS) accumulation of AZ proteins. Previous work has dissected the phenomenon with expression and deletion copies of bruchpilot (brp) globally and in the MBs. Here, the group extend their findings and lay the ground for molecular mechanisms.

In this paper, the group manipulate autophagy in the MB to incur or reverse PS. On the face of it, and on my first reading, the block of autophagy would induce accumulation of brp. However, the local induction and activation of autophagy in MBs, and the global appearance of PS and sleep need became more convincing, particularly in sensitised backgrounds of 'sleepless' or rescue of autophagy in the MBs in an essentially autophagy null background (ATG7). The organismal work then moved to proteomics and transcriptomics, identifying little change in the transcriptome, but a change in calcium channel subunits (down) and potassium channels (up) which appear to make sense in terms of an activity adjustments corresponding to a homeostatic regulation. There is then a move to describing the spread and nature of the autophagy identifying a global up regulation of Ref(2)P/P62 across the brain when PS is induced by KD of autophagy.

The paper appears overall very clean and polished. I have some minor critiques:

1) is brp a direct target of autophagy? I think this would be good to show.
2) for the oxidative stress test (feeding H_2O_2) - I suspect that this test challenges the gut of the fly and may kill the fly via gastrointestinal activity but does not report on ROS responses in the CNS. Can it be shown that this feeding induces protective signaling mechanisms (NRF2, AP1/Fos/Jun) in the CNS?
3) For the transcriptome, there is changes in 6% of of the transcriptome, how does this compare with the proteome? IT may be a simple stat, but please make the comparison in the text.
4) for the dissected ok107-ATG5-RNAi adult brains, what age are they? state in the text (it is stated in the methods, please state in the main text)
5) Figure 2: Panel B - bring theirs of the two controls together, and show if they are NS when compared together, and the green bar (experimental) moves to the right.
6) As a region that can globally induce PS, the MB is now well studied. I haven't seen any data to date that shows if other structures have been tested such as the Fan shaped body, central complex, ellipsoid body? If not, might it be optimal to tone down the centrality of the MBs to this mechanism?
7) In the discussion, the mention of Alzheimer's seems a little non-specific - it may be better to turn the discussion to the ALS/FTD field, where mutations in genes critical for proteostasis/autophagy, particularly Ref(2)P/P62 itself (the sequestome), and including VCP, Ubiquilin2, C9ORF72, OPTN and CHMP2B - the intersection of these mutations with age may make a better area for immediate relevance due to the known molecular players.

Referee #3:

Toppe et al present an extension of previous findings related to brain aging in the fly. Overall, this is potentially a strong paper with interesting findings. However, there are a number of technical and conceptual concerns that would need to be addressed to make this a suitable candidate for publication.

Conceptual concern: The authors make a statement which is, for me, somewhat contradictory. "resilience to local autophagy impairment
by increasing sleep and extending lifespan" and "MB-specific autophagic disruption also caused non-cell
autonomous accumulation of autophagic substrates across the brain, revealing systemic propagation of proteostatic stress". This is confusing as it implies that the local disruption is both 'good' and 'bad'. I understand that things are rarely this simple (ie good or bad) but, further experimentation is required to provide insight.

One of the major technical concerns that I have relates to Fig 3:

It has long been known that longevity studies are challenging to control and susceptible to genetic background differences between test and control lines:

https://www.nature.com/articles/450165a

The longevity studies should be validated with inducible system(s), e,g, GeneSwitch or auxin-inducible. I am not convinced that the data presented is sufficient to support the claim in the abstract "promotes resilience to local autophagy impairment
by increasing sleep and extending lifespan".

Furthermore, there is an added benefit of using an inducible gene expression system (beyond comparing flies from the same cohort with and without inducer); gaining temporal control would be advantageous given the authors model. I will elaborate below.

The H202 exposure data is interesting. But, unless I'm mistaken the authors haven't examined consumption. This is critical as altered consumption could be a factor underlying sensitibity to H2O2.

If the authors model is correct, wouldn't early life upregulation of BRP promote longevity? Showing this with an inducible system would significantly increase the impact fo the study,

Fig. 4 the compensatory induction of chaperones and small hsps seems to be a plausible explanation for any reported prolongevity effects? This should at least be discussed. Would this contradict the authors model??

Fig 6 data is fascinating and very interesting.

Taken on face value, how does this data fit with model that local MB-specific autophagy impairment is protective?? If the impairment is found throughout the brain?

Again, I have a suggestion to significantly improve the impact of the paper. I think a short-term early life KD of Atg5 in MBs with inducible system would be revealing. As noted above, early life induction of BRP would also be revealing in light of the authors model.

The Fig 6 data is indeed intriguing. Can the authors exclude the possibility that general impairment of the MBs will lead to non-autonomous collapse of proteostasis?
Is this observed if MBs are challenged in other ways? or even ablated?? Is this unique to disruption of autophagy in MBs?

We would like to thank the Editor and the three Reviewers for their careful evaluation of our manuscript and for their constructive feedback. We believe that the comments and questions raised have substantially improved the depth and overall significance of our work. We are therefore pleased to submit our revised manuscript for further consideration.

Below, we provide a detailed, point-by-point response to the reviewers' comments.

Changes made in the manuscript have been highlighted, and new data from revision experiments have been incorporated as main Figure 4 and Figure 8, as well as into Expanded View Figures 1-4 and Appendix Figures S1,S2 and S3.

Referee #1:

In this manuscript the authors follow up on their previous work showing that inhibition of autophagy in Drosophila mushroom body (MB) neurons causes brain-wide increases in the active zone protein Brp. They use RNAi to inhibit autophagy in MB neurons through knockdown of Atg5 and analyse the effects on markers of synaptic plasticity, sleep and lifespan. They show a temporal requirement for Atg5 in MB neurons during early ageing, which correlates with perturbed sleep and increases in lifespan. Importantly, MB-specific expression of Atg7 in Atg7 mutant flies is sufficient to restore normal sleep. The increased lifespan caused by MB-specific loss of Atg5, although small, required Brp expression since a heterozygous Brp mutation abrogates the lifespan extension. Proteomic analysis of brain tissue from MB neuron-specific Atg5 knockdown flies showed pronounced changes in endoplasmic reticulum and chaperone proteins. Increases in specific active zone proteins were also observed while changes in general synaptic vesicle components were much less apparent. Finally, MB-specific Atg5 knockdown caused an intriguing accumulation of Ref(2)P/P62 aggregates throughout the brain. This is a well-executed study that provides further insight into the importance of autophagy in MB neurons in relation to maintenance of brain homeostasis and brain ageing. The data are clear and appropriately interpreted.

The proteomic data provide general insight into the brain-wide molecular changes caused by MB-specific Atg5 knockdown, however they are somewhat preliminary, descriptive and lack detailed follow-up regarding the non-cell autonomous causes of these proteomic changes. Similarly, the brain wide accumulation of Ref(2)P/P62 aggregates is an intriguing result but the mechanisms that enable MB neurons to effect such widespread non cell autonomous molecular changes are not clear. Extending the study to include more insight into the mechanisms involved would increase the significance.

We thank the reviewer for highlighting this important point. The non-cell autonomous accumulation of Ref(2)P/p62-positive aggregates represents an important finding of our study. While a full mechanistic dissection is beyond the scope of this work, we performed several additional experiments to better understand the underlying processes.

First, we tested whether protein aggregates can redistribute from MB neurons to other brain regions by expressing GFP-tagged Ref(2)P specifically in MB Kenyon cells in combination with *Atg5* knockdown (Fig. 8E). Under these conditions, Ref(2)P-GFP-positive puncta became detectable outside the MB, supporting partial spreading of aggregates from the targeted neurons.

Second, we assessed the degradative context of these non-cell autonomous aggregates. While overall ATG8a lipidation in whole-brain lysates was not reduced, indicating that autophagosome formation remains largely intact outside the MB (Fig. 7D), Ref(2)P/P62-positive aggregates frequently co-localized with ATG8a-positive structures (Fig. 8A-C). Ultrastructural analysis further revealed their association with degradative compartments (Fig. 8D).

Importantly, treatment with the autophagy inducer spermidine could significantly reduce the size and intensity of aggregates outside the MB, but did not modulate MB KC aggregates to the same extend (Figure EV4).

Together, these findings suggest that non-cell autonomous accumulation of Ref(2)P/P62 arises from a combination of aggregate redistribution and increased proteostatic burden in surrounding neurons. In this context, the accumulation of aggregates likely reflects a condition in which degradative capacity is challenged relative to cargo load, rather than a complete failure of autophagy.

We are further elaborating this interpretation in our answers to Reviewer 3 and included statements in the manuscript at lines 28-30 (Abstract), 91-93 (introduction), 444-470 (Results), 506-511 (Discussion), 513-516 (Discussion), 530-538 (Discussion).

I have the following comments:

1. It is shown in Figure 2E,F that MB specific expression of Atg7 rescues the sleep phenotypes caused by an Atg7 null mutation. Do Atg7 mutants have reduced lifespan and if so does MB specific Atg7 expression rescue the lifespan phenotype of the mutant flies?

We thank the reviewer for this important question.

$Atg7^{d77}$ mutants exhibit a severely reduced lifespan (Appendix Figure S2) (Juhasz et *al.,* 2007). Re-expression of *Atg7* specifically in MB Kenyon cells resulted in a mild but statistically significant extension of lifespan compared to mutant controls (Appendix Figure S2). This relatively modest rescue is consistent with the restricted number of neurons targeted (~4000 MB Kenyon cells) in an otherwise autophagy-deficient organism, while still indicating a functionally relevant contribution of MB neurons to lifespan regulation. In contrast, pan-neuronal re-expression of *Atg7* in *$Atg7^{d77}$* mutants led to a substantially stronger rescue of lifespan (Appendix Figure S2), highlighting the broader contribution of neuronal autophagy to organismal survival. Importantly, while MB-specific re-expression of *Atg7* results in only a limited rescue of lifespan, it robustly restores normal sleep behavior, underscoring a particularly prominent role of autophagy in MB Kenyon cells for sleep regulation (Fig. 2E, F). These data have been included as Appendix Fig. S2 (related to Fig. 3) and are described in the revised manuscript in lines 181-189. These findings very well complement our pan-neuronal and MB-specific autophagy knockdown longevity data presented in Fig. 3 and 4 and further support a key role of neuronal, and specifically MB autophagy in the regulation of lifespan in *Drosophila*.

2. Please include more details of how proteomic data analyses were performed in the main text and figure legends. How old were the flies used for the proteomics? Please state the statistical cutoff used and whether a fold change cutoff was used. In the figure legends for the volcano plots describe what the lighter dots represent. Presumably these are below the statistical cutoff? The fold changes of the presynaptic AZ proteins labelled in Figure 4A are very small. It would be interesting to include volcano plots highlighting the proteins with the greatest fold change/p value. Presumably many of these are endoplasmic reticulum and chaperone proteins. Tables with details of the proteins and their fold change/p value would also be helpful. Please include the details of the significantly upregulated and downregulated proteins in the proteomic analysis as supplemental tables and upload the raw proteomic data to a publicly accessible database.

We thank the reviewer for pointing out these important missing informations. We included information about the age of the flies used for proteomics in the main text (Line 269-271) as well as in the figure legends for Fig. 5, 6 and 7. For Proteomics, we used 10d old flies raised at 29°C to stay consistent with the experimental conditions used in our previous publication (Bhukel et al, 2019). We also added a sentence stating the statistical cutoff used in the corresponding figure

legends of Fig 5,6 and 7.

We further included Tables of all significantly up- and down regulated protein hits with their p-values and fold changes (Appendix Tables S1 and S2), as well as volcano plots highlighting the Top20 up- and downregulated protein hits with the highest p-value in the Appendix Fig. S4. Indeed, multiple ER related proteins (ergic53, CaBP1, ScpX) as well as the AZ protein Liprin-alpha were among the Top20 upregulated upon MB-specific *Atg5* knockdown. We added this information in Lines 317-318.

The Raw proteomic data will be uploaded to a publicly accessible database (https://www.ebi.ac.uk/pride/) on the 9th of April 2026. We sincerely apologize this delay, as we previously uploaded the wrong data set from another experiment. Below are the accession and log-in details.

Furthermore, an excel file with processed proteomics data displaying all identified proteins with their p-value and fold-change was also included in the source data for Figure 6.

Submission details:

Project Name: Autophagy-driven Presynaptic Reorganization as a Molecular Signature of Brain Resilience

Project accession: PXD076163

Project DOI: Not applicable

Reviewer access details

Log in to the PRIDE website using the following details:

Project accession: PXD076163

Token: 6ROGCuUQaCm1

Alternatively, reviewer can access the dataset by logging in to the PRIDE website using the following account details:

Username: reviewer_pxd076163@ebi.ac.uk

Password: DiixYd39g5aS

3. Is there increased ubiquitination of any of the proteins whose levels are altered in MB-specific Atg5 knockdown flies?

In order to answer the reviewers question, we performed a proteomic approach for the specific detection of ubiquitinated proteins using a commercially available Ubiquitination enrichment kit (https://www.cellsignal.com/products/proteomic-analysis-/products/hs-ubiquitin-sumo-remnant-motif-k-epsilon-gg-kit/59322).

For this purpose, we homogenized 10 day old *ok107*-Gal4>UAS-*Atg5*-RNAi fly heads as well as *ok107*-Gal4/+ controls in the presence of proteasomal and deubiquitinase inhibitors and subjected the S1 fraction for DiGG enrichment as well as for global proteomic MS analysis without prior enrichment. With this approach we could successfully identify around 15.000 ubiquitination sites. Importantly, significantly more ubiquitinated peptides were detected in the *Atg5* knockdown group compared to the control (Reviewer Fig. 1A) consistent with our previous IHC data (Fig. 7C). After data processing and normalization of the ubiquitinome data to the global proteome using linear regression, we identified approximately 450 peptides that showed significantly increased ubiquitination levels in the *Atg5* MB knockdown group compared to the control. Importantly,

among these, we detected two hyperubiquitinated peptides mapping to BRP, as well as a single ubiquitination site mapping to Unc13 (Reviewer Fig. 1B, C).

While these preliminary findings highlight the potential of this dataset to provide valuable insights into ubiquitination dynamics in the context of MB autophagy impairment, as well as in the regulation of ubiquitination of specific AZ proteins, we are concerned that inclusion of these data would substantially increase the complexity of the manuscript and risk overloading its central narrative. We have therefore chosen not to include this dataset at this stage. However, should the reviewers consider its inclusion essential, we would be happy to incorporate these data into the revised manuscript.

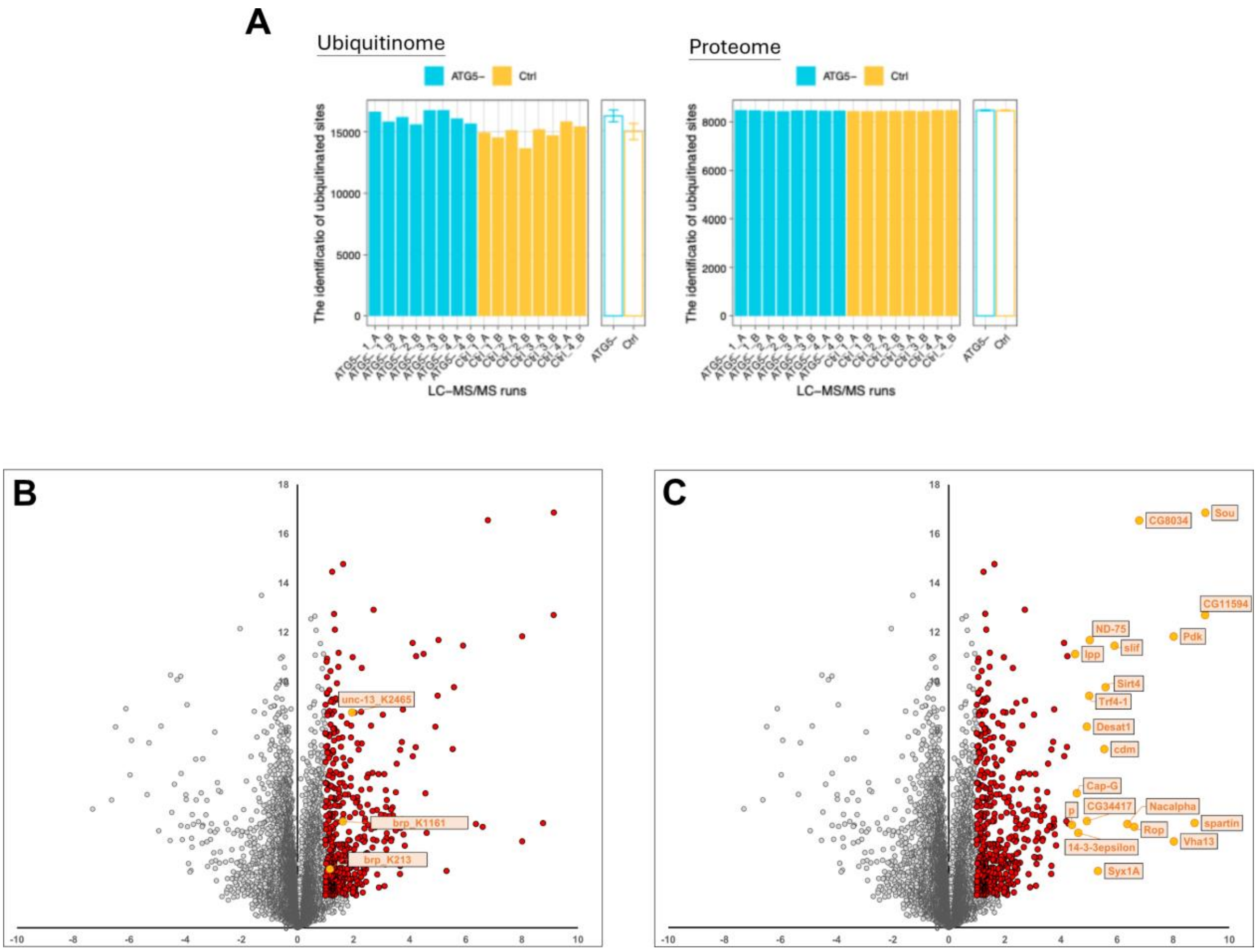

Reviewer Figure 1: Preliminary results from Ubiquitinome proteomics of MB autophagy impaired flies

4. There are clear parallels between this study and recent work showing non cell autonomous integrated stress response activation due to inhibition of photoreceptor neuron activity in Drosophila (PMID: 39747204), which also observed non cell autonomous accumulation of Ref(2)p. Apart from the increased expression of ER stress response proteins that is reported from the proteomics data, can the authors test whether there is direct evidence of ISR activation cell autonomously or throughout the brain with MB-specific Atg5 knockdown? The authors should also discuss the links between the current study and the findings in PMID: 39747204.

We thank the reviewer for highlighting this important connection.

We agree that our findings show conceptual parallels to recent work demonstrating non-cell autonomous proteostatic responses upon neuronal perturbation (PMID: 39747204). In that study, inhibition of photoreceptor activity induced the formation of Ref(2)P-positive, membrane-less

granules in distant neurons, accompanied by activation of the integrated stress response (ISR), including increased phosphorylation of eIF2α and accumulation of ATF4 inside stress granules.

To directly address this point, we assessed ISR activation in our system. While we observe widespread, non-cell autonomous accumulation of Ref(2)P/P62-positive structures upon MB-specific autophagy impairment (Figure EV3A-F), we did not detect increased phosphorylation of eIF2α or a general upregulation of ATF4 (Figure EV3A-E), indicating that the observed phenotype does not involve a global activation of the ISR. Nevertheless, we could observe an accumulation of ATF4 (Figure EV3F), but not of the RNA granule marker me31B (Figure EV3I), in some local populations of Ref(2)P/P62 aggregates.

In our interpretation, these findings suggest that the observed brain-wide proteostatic remodeling is not primarily driven by a canonical ISR program, but instead may reflect a mechanistically distinct form of non-cell autonomous proteostasis regulation. This view is further supported by the properties of the observed aggregates, which appear to lack key features of canonical stress granules, as described in the revised manuscript. We nevertheless acknowledge that alternative interpretations are possible and would welcome the reviewers' perspective on this point.

More broadly, our results suggest that non-cell autonomous proteostatic responses can arise through multiple, context-dependent mechanisms, including ISR-driven signaling as well as autophagy-linked redistribution of proteostatic load. Importantly, in our system, this redistribution is associated with adaptive organismal outcomes, including increased sleep (Fig. 2) and extended lifespan (Fig. 3, 4), consistent with a context-dependent adaptive response to proteostatic stress.

To further address this point, we have included the ISR-related data as Expanded View Figure 3 and have incorporated its description in both the Results (lines 405-419) and Discussion sections (lines 521-526).

Referee #2:

Toppe et al., in this report build on the work of the Sigrist lab identifying an accumulation of pre-synaptic active zone (AZ) proteins increasing sleep need in aging flies termed 'PreScale'. This accumulation appears to be a mechanism preserving synaptic function and a possible homeostasis adjustment supporting aging. Critically it can be activated in the mushroom body (MB), a structure critical to learning and memory and sleep need, and activation in the MB can trigger a pan-CNS 'PreScale' (PS) accumulation of AZ proteins. Previous work has dissected the phenomenon with expression and deletion copies of bruchpilot (brp) globally and in the MBs. Here, the group extend their findings and lay the ground for molecular mechanisms. In this paper, the group manipulate autophagy in the MB to incur or reverse PS. On the face of it, and on my first reading, the block of autophagy would induce accumulation of brp. However, the local induction and activation of autophagy in MBs, and the global appearance of PS and sleep need became more convincing, particularly in sensitised backgrounds of 'sleepless' or rescue of autophagy in the MBs in an essentially autophagy null background (ATG7). The organismal work then moved to proteomics and transcriptomics, identifying little change in the transcriptome, but a change in calcium channel subunits (down) and potassium channels (up) which appear to make sense in terms of an activity adjustments corresponding to a homeostatic regulation. There is then a move to describing the spread and nature of the autophagy identifying a global up regulation of Ref(2)P/P62 across the brain when PS is induced by KD of autophagy. The paper appears overall very clean and polished.

I have some minor critiques:

1) is brp a direct target of autophagy? I think this would be good to show.

We thank the reviewer for this insightful and constructive suggestion.

To directly assess whether BRP undergoes autophagic degradation, we performed a GFP conversion (liberation) assay to monitor its autophagic-lysosomal turnover in the context of MB-specific autophagy impairment. In this assay, delivery of GFP-tagged proteins to acidic compartments such as lysosomes and autolysosomes results in the release of free GFP, detectable as a ~27 kDa band by Westernblot (PMID: 24667416; PMID: 25481477).

Using flies carrying two additional genomic copies of GFP-tagged BRP (170 kDa isoform), we observed a pronounced reduction of the free GFP signal upon MB-specific *Atg5* knockdown, indicating slowed lysosomal degradation of BRP (Figure EV2). These results strongly support the conclusion that BRP is degraded via the autophagic-lysosomal pathway and represents a direct target of autophagy.

Consistent with our findings, recent work (PMID: 36640763) identified BRP as an autophagy substrate in dorsal cluster neurons of the developing nervous system, based on co-localization of a GFP-tagged BRP construct with the autophagosomal marker ATG8a.

These data have now been included as Expanded View Figure 2 and are described in the revised manuscript in lines 333-343.

2) for the oxidative stress test (feeding H2O2) - I suspect that this test challenges the gut of the fly and may kill the fly via gastrointestinal activity but does not report on ROS responses in the CNS. Can it be shown that this feeding induces protective signaling mechanisms (NRF2, AP1/Fos/Jun) in the CNS?

We thank the reviewer for this important and insightful comment. We agree that systemic oxidative stress paradigms, such as H_2O_2 feeding, may primarily affect peripheral tissues and do not necessarily reflect ROS responses within the central nervous system. To directly address this point, we assessed activation of stress-responsive signaling pathways in the brain following H_2O_2 treatment. Specifically, we performed Western blot analysis of phosphorylated JNK (p-JNK), a key mediator of oxidative stress signaling, in brain lysates from 5-day-old flies with MB-specific *Atg5* knockdown (*vt30559*-Gal4>UAS-*Atg5*-RNAi) and corresponding genetic controls (*vt30559*-Gal4/+). Flies were exposed to 2% H_2O_2 for 24 h or 48 h prior to dissection. Under these

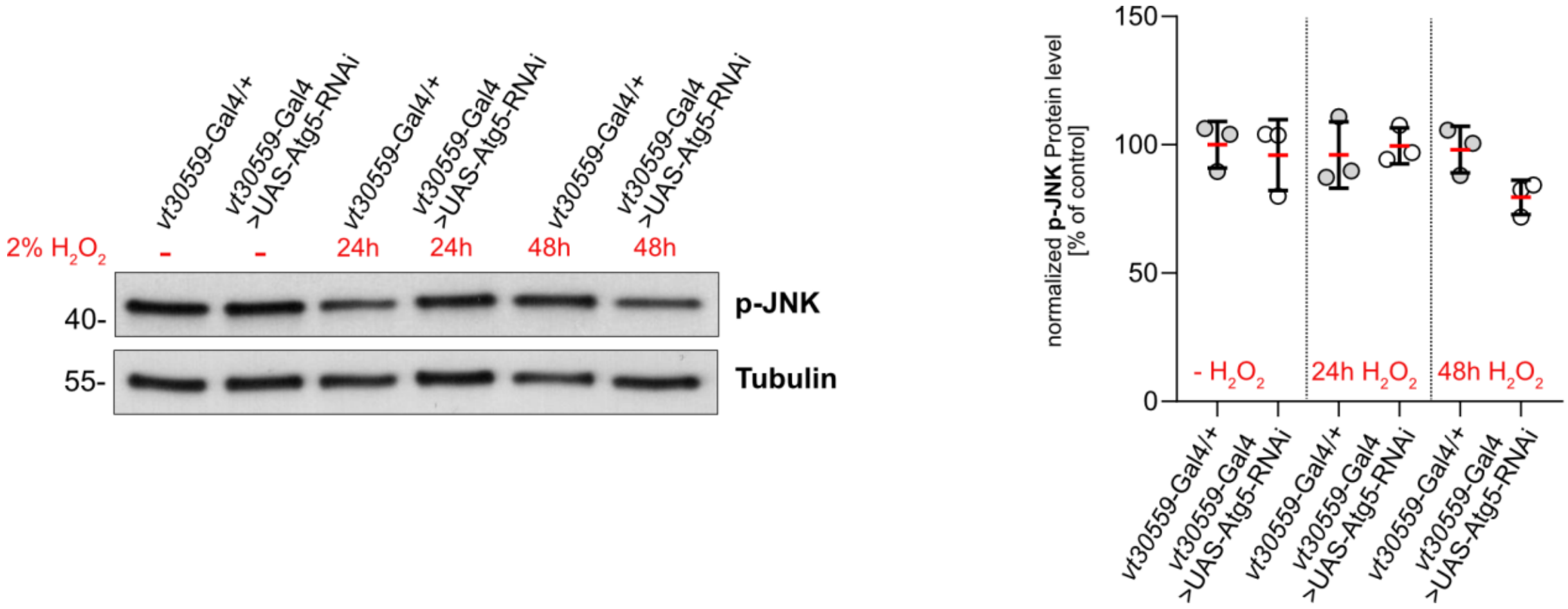

conditions, we did not detect a significant increase in p-JNK levels in the brain in either genotype, indicating that H_2O_2 feeding does not robustly induce JNK-dependent stress signaling in the CNS in this experimental context (see Reviewer Figure 2)

Reviewer Figure 2: Analysis of brain p-JNK levels upon H_2O_2 treatment in MB autophagy impaired flies.

Importantly however, these results also suggest that MB-specific autophagy impairment itself does not lead to chronic activation of JNK stress signaling in the brain, despite the pronounced proteostatic imbalance observed in our study. This further supports the notion that these flies maintain a resilient state at the level of neuronal stress responses.

3) For the transcriptome, there is changes in 6% of of the transcriptome, how does this compare with the proteome? IT may be a simple stat, but please make the comparison in the text.

For label-free quantitative proteomics of hand-dissected brain homogenates, we detected 3518 individual proteins (present in ≥3 of 4 replicates), from which 13% (471 proteins) were significantly upregulated, and 5% (172 proteins) significantly downregulated in response to MB-specific *Atg5* knockdown.
For RNA Seq Data acquired from whole heads and performed by the Lexogen company, we identified a total of 10262 transcripts, from which only 2,2% (228) were significantly up-, and 2,6% (270) were significantly downregulated.

The 6% refers to a cross comparison of proteomic and transcriptomic hits, revealing that only 6% of all significantly changed proteins showed corresponding significant alterations at the transcript level.

We added all the above information in the proteomics (lines 272-273) and RNA Seq result part (Lines 349-351) and hope that this will clarify the results of the different experiments.

4) for the dissected ok107-ATG5-RNAi adult brains, what age are they? state in the text (it is stated in the methods, please state in the main text)

We are sorry for the omission. For proteomics, we used 10d old flies raised at 29°C to stay consistent with the experimental conditions used in our previous publication (Bhukel et al, 2019). We added this information in the main text (Lines 269-271) as well as in the Figure legends for Fig. 5,6 and 7.

5) Figure 2: Panel B - bring theirs of the two controls together, and show if they are NS when compared together, and the green bar (experimental) moves to the right.

We have revised the figure in accordance with the reviewers' suggestion and included statistical comparisons between the two control groups. Despite extensive backcrossing of all fly lines into the same w^{1118} background for at least six generations, the two control groups shown in Figure 2A,B exhibited significant differences in both daytime and nighttime sleep when compared to each other. However, these differences were no longer apparent when considering total 24-hour sleep. The corresponding statistical analyses have now been added to the figure.

Importantly, the differences in sleep between the two control groups do not affect the overall interpretation of the data. Flies with MB-specific autophagy impairment (*vt30559*-Gal4>UAS-*Atg5*-RNAi) consistently displayed a significant increase in total, daytime, and nighttime sleep compared to both control groups.

6) As a region that can globally induce PS, the MB is now well studied. I haven't seen any data to date that shows if other structures have been tested such as the Fan shaped body, central complex, ellipsoid body? If not, might it be optimal to tone down the centrality of the MBs to this mechanism?

We thank the reviewer for this important and constructive point.

To directly address whether the observed PreScale response is specific to MB neurons or can also be triggered from other brain regions, we performed additional experiments in which autophagy was impaired in distinct neuronal populations outside the MB. Specifically, we knocked down *Atg5* in R2/R5 neurons (*R58H05*-Gal4), dorsal fan-shaped body neurons (*R23E10*-Gal4), and helicon cells (*R24B11*-Gal4), and assessed BRP levels by immunohistochemistry in comparison to age-matched genetic controls (Appendix Figure S1).

These analyses were performed in 15-day-old flies, a time point at which MB-induced PreScale is most pronounced in our system (Fig. 1). In contrast to MB-specific *Atg5* knockdown, we did not observe a comparable brain-wide increase in BRP levels upon autophagy impairment in any of these neuronal populations. Instead, BRP levels were unchanged or even reduced (notably in R2/R5 neurons and helicon cells).

These results indicate that the MB has a distinct capacity to induce non-cell autonomous PreScale, rather than representing a general property of neuronal autophagy impairment across the brain. Consistent with this, previous work by Bhukel et al., 2019 (PMID: 30899013) also did not observe PreScale activation upon autophagy disruption in olfactory projection neurons (*GH146*-Gal4).

We have included these data as Appendix Figure S1 and adjusted the manuscript text accordingly (lines 133-139 (Results), lines 552-554 (Discussion)), moderating the generality of our conclusions while highlighting the specific role of the MB in this process.

Taken together, we believe that these findings complement and reinforce our claim that the MB, and the functional status of MB autophagy, acts as a major regulator of brain-wide PreScale activation.

7) In the discussion, the mention of Alzheimer's seems a little non-specific - it may be better to turn the discussion to the ALS/FTD field, where mutations in genes critical for proteostasis/autophagy, particularly Ref(2)P/P62 itself (the sequestome), and including VCP, Ubiquilin2, C9ORF72, OPTN and CHMP2B - the intersection of these mutations with age may make a better area for immediate relevance due to the known molecular players.

We thank the reviewer for this insightful comment and for highlighting the broader relevance of our findings. In response, we have revised the Discussion as follows: "The identification of a non-cell autonomous synaptic resilience program centered on presynaptic remodeling in response to autophagy impairment has important implications beyond basic neurobiology. Notably, many neurodegenerative disorders are associated with mutations in genes involved in proteostasis and autophagy, including p62/SQSTM1, as well as VCP, UBQLN2, OPTN, and C9ORF72—several of which have been implicated in amyotrophic lateral sclerosis (ALS) and frontotemporal dementia (FTD) and emerge as key factors of stress and aging." (lines 590-596).

Referee #3:

Toppe et al present an extension of previous findings related to brain aging in the fly. Overall, this is potentially a strong paper with interesting findings. However, there are a number of technical and conceptual concerns that would need to be addressed to make this a suitable candidate for publication.

Conceptual concern: The authors make a statement which is, for me, somewhat contradictory. "resilience to local autophagy impairment by increasing sleep and extending lifespan" and "MB-specific autophagic disruption also caused non-cell autonomous accumulation of autophagic

substrates across the brain, revealing systemic propagation of proteostatic stress". This is confusing as it implies that the local disruption is both 'good' and 'bad'. I understand that things are rarely this simple (ie good or bad) but, further experimentation is required to provide insight.

We thank the reviewer for raising this important conceptual point.

We agree that the coexistence of beneficial organismal effects (increased sleep and lifespan) with widespread non-cell autonomous accumulation of proteostatic markers appears counterintuitive. We interpret this as a context-dependent adaptive response. Importantly, based on our previous work (Bhukel et al., 2019), MB-specific *Atg5* knockdown does not increase apoptosis markers (e.g., DCP1) nor does it reduce MB neuron number, indicating that neurons tolerate the accumulation of ubiquitinated protein aggregates without overt neurodegeneration in this context. Moreover, our ultrastructural analyses (Fig. 8D) reveal the presence of autolysosomal compartments associated with these aggregates in neurons outside the MB, indicating active engagement of degradative pathways rather than a complete failure of proteostasis (see also answer to Reviewer 1)

Together, these findings support a model in which local autophagy impairment induces a redistribution of proteostatic burden that is tolerated and actively processed. In this framework, the observed phenotype is consistent with a hormesis-like response, in which moderate proteostatic stress enhances engagement of degradation pathways and may enhance the efficiency of cellular detoxification, thereby contributing to organismal resilience.

We adjusted our statements in the abstract accordingly (Lines 28-30) and also further elaborate this interpretation in the results (Lines 444-470) and discussion (Lines 530-538) sections.

One of the major technical concerns that I have relates to Fig 3: It has long been known that longevity studies are challenging to control and susceptible to genetic background differences between test and control lines: https://www.nature.com/articles/450165a The longevity studies should be validated with inducible system(s), e,g, GeneSwitch or auxin-inducible. I am not convinced that the data presented is sufficient to support the claim in the abstract "promotes resilience to local autophagy impairment by increasing sleep and extending lifespan". Furthermore, there is an added benefit of using an inducible gene expression system (beyond comparing flies from the same cohort with and without inducer); gaining temporal control would be advantageous given the authors model. I will elaborate below.

We thank the reviewer for this important and insightful comment regarding the robustness and interpretation of our longevity experiments.

We minimized potential genetic background effects by extensive isogenization (≥6 generations) of all fly lines used for behavioral and lifespan analyses. However, we fully agree that background effects cannot be entirely excluded and therefore implemented an independent validation strategy using an inducible system.

To this end, we employed the auxin-inducible gene expression system (AGES), which allows temporally controlled activation of Gal4-dependent transgenes without the need for temperature shifts. Based on our analysis of PreScale dynamics (Fig. 1), we focused on the early adult period (day 1–15), during which brain-wide BRP upregulation is most pronounced.

Using this system, we induced MB-specific *Atg5* knockdown during development and early adult life until day 15. This intervention robustly triggered PreScale, as evidenced by increased brain-wide BRP levels at day 15 (Fig.4B, C), and was sufficient to significantly extend lifespan in both male and female flies (Fig. 4E, F).

To further test whether PreScale itself is sufficient to promote longevity, independent of autophagy impairment, we employed a FlySAM2.0-based approach to induce BRP upregulation. The BRP FlySam2.0 vector combines a UAS-controlled dCas9 variant with a *brp* promoter specific gRNA for transcriptional activation of the *brp* gene locus, and this genetic approach robustly increased BRP levels at the larval neuromuscular junction (driven by *ok6*-Gal4) (Appendix Figure S3A, B) as well as in the adult brain (*elav*-Gal4) (Appendix Figure S3C, D). The Auxin-dependent temporal induction of BRP expression in early adult life (day 1–15 post-eclosion) mildly increased brainwide BRP levels (Fig. 4H, I), and resulted in a significant extension of lifespan compared to untreated genetic controls (Fig. 4L, M).

Together, these results provide independent validation of our longevity findings and demonstrate that early-life activation of PreScale is sufficient to promote lifespan extension, thereby strengthening our conclusion that this mechanism contributes to organismal resilience.

These data have been included as the new Fig. 4 and are described in the revised manuscript Lines 26-27 (Abstract), Lines 89-91 (Introduction), Lines 230-256 (Results), Lines 1133-1144 (Methods and Protocols).

The H202 exposure data is interesting. But, unless I'm mistaken the authors haven't examined consumption. This is critical as altered consumption could be a factor underlying sensitibity to H2O2.

We thank the reviewer for this important and insightful comment.

We agree that differences in food intake could potentially confound the interpretation of H_2O_2 sensitivity, as well as other behavioral phenotypes such as sleep and lifespan. To directly address this, we quantified feeding behavior using an automated FlyPad system (PMID: 25087594).

We did not detect major differences in food consumption between MB-specific *Atg5* knockdown flies (*vt30559*-Gal4>UAS-*Atg5*-RNAi; *ok107*-Gal4>UAS-*Atg5*-RNAi) and their respective genetic controls (*vt30559*-Gal4/+; *ok107*-Gal4/+), as measured by the number of sips per hour at 5 days of age. Importantly, this was consistent both under normal feeding conditions (Expanded View Figure 1F) and following exposure to 2% H_2O_2 (Expanded View Figure 1E).

These results exclude altered feeding behavior as a confounding factor and support the conclusion that the observed differences in stress sensitivity, sleep, and lifespan are a direct consequence of MB-specific autophagy impairment and the associated activation of PreScale.

These data have been included as Expanded View Fig. 1E, F and are described in the revised manuscript in Lines 206-211 (Results) and Lines 939-951 (Methods and Protocols)

If the authors model is correct, wouldn't early life upregulation of BRP promote longevity? Showing this with an inducible system would significantly increase the impact for the study,

We thank the reviewer for this insightful suggestion.

Indeed, we directly tested this hypothesis using an inducible system and found that early-life upregulation of BRP is sufficient to promote longevity. Specifically, using the AGES system, we induced pan-neuronal BRP expression during early adult life (day 1–15 post-eclosion), without additional manipulation of autophagy.

Indeed, this temporally restricted increase in BRP levels resulted in a significant extension of lifespan compared to genetic controls without auxin supplementation, while auxin treatment of

control flies did not have the same pro-longevity effect (Fig. 4L, M). These findings demonstrate that early-life activation of PreScale is sufficient to enhance organismal survival.

These data have been included as Fig. 4 and are described in the revised Lines 26-27 (Abstract), Lines 89-91 (Introduction), Lines 230-256 (Results), and represent a key mechanistic validation of our model.

The compensatory induction of chaperones and small hsps seems to be a plausible explanation for any reported prolongevity effects? This should at least be discussed. Would this contradict the authors model??

We thank the reviewer for this important and insightful comment.

We agree that the induction of chaperones and small heat shock proteins represents a plausible mechanism contributing to the observed pro-longevity effects, and we have now incorporated this aspect into the Discussion (Lines 572-575).

At the same time, our data indicate that PreScale activation, and in particular BRP upregulation, plays a central and causal role in mediating these effects. Specifically, genetic reduction of BRP levels in the context of MB-specific *Atg5* knockdown attenuates the lifespan extension phenotype (Fig. 3I,J), demonstrating that sufficient BRP levels are required for the pro-longevity effect.

Importantly, using an inducible system, we further show that early-life upregulation of BRP alone is sufficient to significantly extend lifespan, independent of direct manipulation of autophagy (Fig. 4H-M). This provides strong causal support for a central role of PreScale in promoting longevity

We therefore propose that chaperone induction and PreScale activation are not mutually exclusive but may act in parallel as part of a coordinated adaptive response to proteostatic stress. In this framework, PreScale represents a key driver of the longevity phenotype, while chaperones and small heat shock proteins may further support proteostasis and contribute to the overall resilience of the system.

Fig 6 data is fascinating and very interesting. Taken on face value, how does this data fit with model that local MB-specific autophagy impairment is protective?? If the impairment is found throughout the brain?

We thank the reviewer for this insightful and important question. We agree that, at first glance, the brain-wide occurrence of Ref(2)P-positive aggregates may appear difficult to reconcile with a protective, MB-specific autophagy impairment.

We have clarified this point in the revised manuscript (Discussion, Lines 530-538, Lines 511-516). Specifically, we now emphasize that autophagy is selectively disrupted within MB Kenyon cells, whereas degradative pathways remain active in neurons outside the MB. As stated in the Discussion, “the widespread Ref(2)P/P62 aggregates in non-targeted brain areas were often associated with mCherry-ATG8a-positive structures and could be identified in degradative lysosomal compartments […], suggesting that autophagic processes remain active in these cells.” We further note that “the accumulation of Ref(2)P/P62-positive material indicates an imbalance between proteostatic load and degradative capacity, rather than a complete block in autophagic flux” .

Furthermore, we highlight that “moderate, spatially distributed proteostatic stress elicits an adaptive response,” (Lines 531-532) which we interpret as consistent with a hormesis-like mechanism that supports organismal resilience despite the presence of aggregates.

Together, these points support our interpretation that MB-specific autophagy impairment induces a system-level response combining local PreScale activation with a broader redistribution of proteostatic load, while degradation remains functional outside the MB.

Again, I have a suggestion to significantly improve the impact of the paper. I think a short-term early life KD of *Atg5* in MBs with inducible system would be revealing. As noted above, early life induction of BRP would also be revealing in light of the authors model.

Using the Auxin-inducible system, we aimed to post-developmentally knock down *Atg5* specifically within the MB KCs (*vt30559*-Gal4,AGES>UAS-*Atg5*-RNAi) between day 1 until day 15. Unfortunately, this intervention did not result in any apparent buildup of autophagic defects such as Ref(2)P/P62 aggregates, not even cell-autonomously within the MB KCs. While this might be due to a very slow turnover rate of ATG5 limiting the efficacy of the *Atg5*-RNAi, we decided to not further test the longevity in this experimental context.

However, as described above, when we induced MB-specific *Atg5* knockdown by auxin supplementation already during development until day 15, this intervention robustly triggered PreScale, as evidenced by increased brain-wide BRP levels at day 15, and was sufficient to significantly extend lifespan in both male and female flies (Fig. 4A-F), providing further evidence that activation of PreScale in response to MB autophagy impairment at an early time-point in life (i.e. 15d) is important to promote longevity.

The Fig 6 data is indeed intriguing. Can the authors exclude the possibility that general impairment of the MBs will lead to non-autonomous collapse of proteostasis? Is this observed if MBs are challenged in other ways? or even ablated?? Is this unique to disruption of autophagy in MBs?

To address whether the observed non-cell autonomous proteostasis imbalance is a general consequence of MB dysfunction, we considered both previous work and new experimental approaches.

Previous studies (Bhukel et al., 2019) manipulated MB neuronal activity using UAS-Shibire^{ts} and UAS-dTrpA1 approaches. However, these interventions caused pronounced developmental defects, including accumulation of BRP within MB Kenyon cell bodies, making them unsuitable for assessing non-cell autonomous proteostasis effects in a controlled manner.

To more directly test this question, we induced proteostatic stress in MB neurons by independent means during revision. Specifically, we overexpressed aggregation-prone mutant proteins (Hsc70.3^{D231S} and NinaE69D) using the *vt30559*-Gal4 driver. Especially Hsc70.3^{D231S} overexpression resulted in robust accumulation of Ref(2)P/P62 within MB Kenyon cells, confirming strong local proteostatic stress (Reviewer Figure 3C).

Importantly, however, we did not observe a comparable increase in Ref(2)P/P62 aggregates in other brain regions (Reviewer Figure 3A, B). As degradation pathways (including autophagy) inside the MB remain functional upon Hsc70.3^{D231S} overexpression, these findings underline that the non-cell autonomous spreading of Ref(2)P is rather a specific consequence in response to impaired autophagy, rather than to general proteostatic stress.

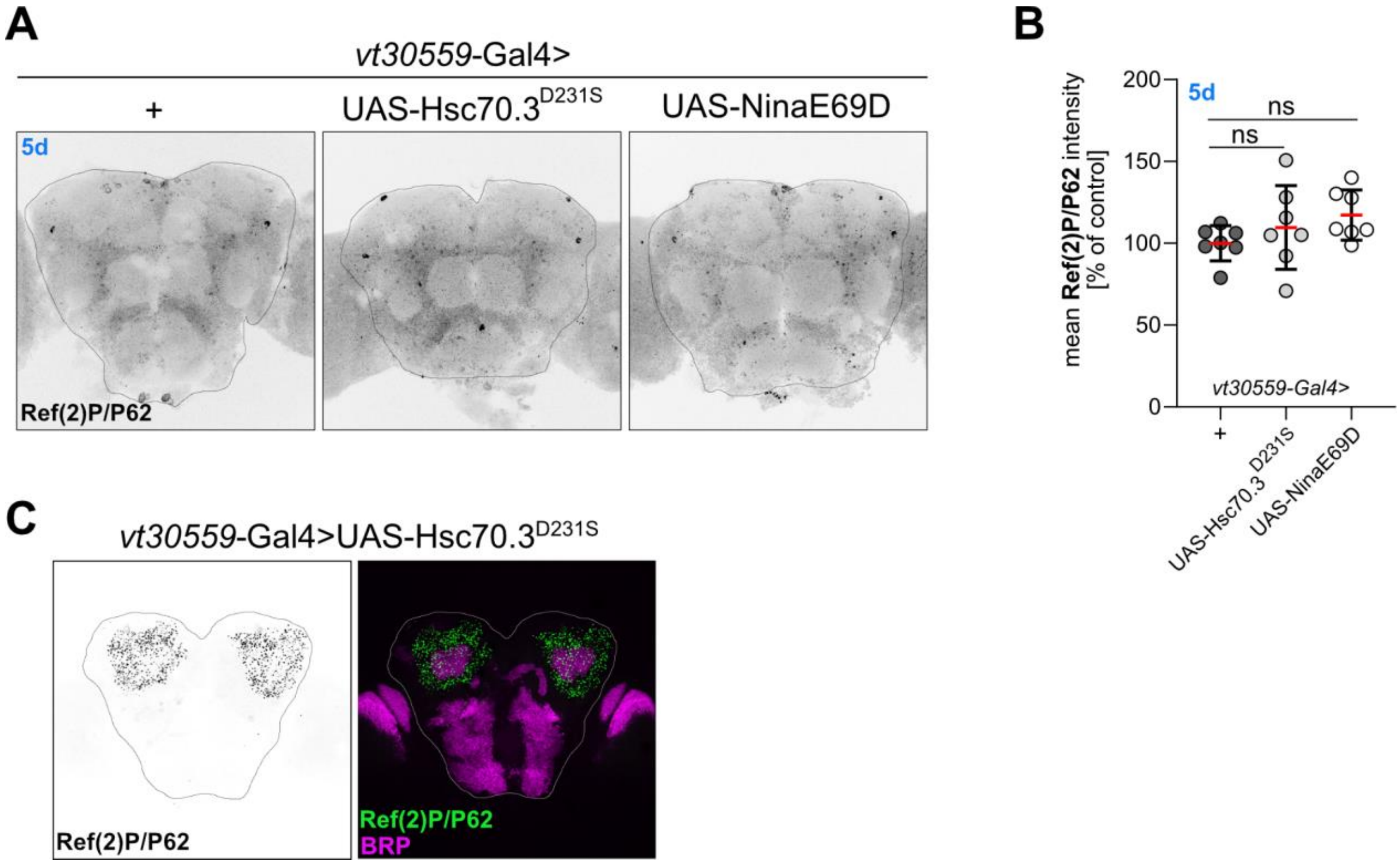

Reviewer Figure 3: MB-specific induction of proteostatic stress by overexpression of aggregate-prone proteins.

1st Revision - Editorial Decision **6th May 2026**

Dear Stephan,

Thank you for the submission of your revised manuscript (EMBOJ-2025-122093R) to The EMBO Journal and your patience during re-review. Your revision has now been seen by the three original referees, who had previously reviewed the first version of the manuscript, and we have received their comments, which are appended below.

As you will see, all three referees are satisfied with the depth and quality of the revision, and they find the new version of the manuscript substantially improved and the majority of their initially raised criticisms and concerns adequately addressed. Referees #1 and #2 only raise a couple of minor points that could optionally be added to the Discussion of the final version of the manuscript. Referee #3, on the other hand, still have some remaining concerns regarding some of the data shown in Figure 4 - mostly with regard to the control used in panels 4B-4F and its appropriateness. We would kindly request that you fully address the remaining concerns in a final version of the manuscript, either by clarifying or by adding new data, as necessary. Please also submit along with your revised manuscript a detailed point-by-point response addressing all remaining referees' comments.

From the editorial side, there are also a few changes we need you to make in the final version of your manuscript, before we can move forward with the next steps towards publication in The EMBO Journal:

- Figures should be removed from the manuscript file (only remain uploaded to our system as individual files); only their legends should remain in the manuscript, placed below the References list.

- The nomenclature for Expanded View Figures should be "Figure EV1-EV4" (instead of "Expanded View Figure 1-4") throughout the manuscript.

- Please change heading "Literature" to "References".

- Please change heading "Methods and Protocols" to "Methods".

- Thank you for depositing your mass spectrometry data to a public repository. The RNA-sequencing data produced in this study should also be deposited in an appropriate database, and the database, dataset ID, and specific URL should be provided in the Data availability section of the revised manuscript. Please note that the URLs provided in this section should be the specific URLs of the deposited datasets, not the homepage URLs of the databases.

- Figure callouts for the panels of each Figure should be listed in the manuscript sequentially.

- We noticed that callouts for Figure 3A-B are missing.

- The title page of the Appendix PDF file should contain heading "Appendix for", followed by the manuscript's title and a Table of Contents including page numbers for all listed items; nomenclature should be corrected to "Appendix Figure S#" and "Appendix Table S#" throughout the Appendix and the manuscript.

- Please note that EMBO press papers are accompanied online by:
A) a short (2 sentences) summary of the findings and their significance,
B) 2-5 short bullet points highlighting the key results, and
C) a synopsis image in .jpg or .png format that is exactly 550 pixels wide x 300 pixels high. Please note that all text needs to be legible at this size.
Please upload this information along with your revised manuscript (the text for A and B should be provided in a separate Word file).

- Our data editors have checked your Figures and their legends, and raised the following queries. Please address all points below completely in your revised manuscript (all changes should be highlighted or "tracked"):
1. Please provide the exact p-values in the legends of Figures 1A', B', D', E'; 2B, D, F; 3B, D, F, H, J; 4C, F, K, M; 6, D; EV1 F, EV2 B, EV3 C, H; EV4 C, D.
2. Please indicate the statistical test used for data analysis in the legends of Figures 5A-D.
3. Please note that information related to "n" is missing in the legend of Figure 7A.

- The manuscript sections need to be named and ordered as follows: Title page - Abstract - Introduction - Results - Discussion - Methods - Data Availability - Acknowledgements - Disclosure and Competing Interests Statement - References - Figure Legends - main Tables (if there are any) - Expanded View Figure Legends.

- Please also note that as part of the EMBO Press transparent editorial process, The EMBO Journal publishes online a Peer Review File along with each accepted manuscript. This File will be published in conjunction with your paper and will include the

referee reports, your point-by-point responses and all pertinent correspondence relating to the manuscript. Your Author's Checklist will also be published at the end of the Peer Review File. Please let us know in case you want to remove any data or figures from your point-by-point responses before they are published as part of the Peer Review File. Retaining unpublished data in the Peer Review File means that these count as published and that the Peer Review File would need to be referenced in future publications. Please let the editorial office know in case you want to remove any data from this file (contact@embojournal.org).

We look forward to seeing a final version of your manuscript as soon as possible. Please let us know if you have any questions and use this link to submit your revision: https://emboj.msubmit.net/cgi-bin/main.plex.

Best regards,

Ioannis

Ioannis Papaioannou, PhD
Editor, The EMBO Journal
i.papaioannou@embojournal.org

Referee #1:

The revised manuscript addresses my previous comments and is substantially improved. There are some very interesting additional observations and I am fully satisfied with the revised version.

Regarding the accumulation of ATF4 in some local populations of Ref(2)P/P62 aggregates, this is very interesting and may suggest that specific types/populations of neurons are sensitised to accumulate ATF4. Investigating this is beyond the scope of the current work but is something that could be addressed in future studies by using single cell/single nuclear RNA-sequencing of brain or head tissue in this model.

Referee #2:

As per my comments in previous iterations, and not to repeat too much, the Sigrist lab have further explored their observation that impairment of autophagy in mushroom bodies generates a wider modification of active zone accumulation across the brain, improving sleep parameters and lifespan. This is explored at the molecular level with proteomic and transcriptomic changes characterised. The observation that degradative components from the MBs spread across the brain is interesting and although not mechanistically linked as yet to the active zone component increase, further describes aspects of the MB autophagy block as it affects the whole brain. The inclusion of autophagy block on other identifiable structure (particularly related to sleep) with induction of the brain-wide effect further ratifies the MBs as critical in this novel mechanism in neuronal homeostasis and aging. They have undertaken a number of novel experiments at the suggestion of the reviewers that have sharpened and refined the data. If anything, the dataset is now rather extensive, the authors have gone the extra yards. The paper is to my mind robust, of strong interest and high scientific merit. I have one v. minor comment, (which is optional) when discussing the lack of ISR response, to also add that the directed experiments are also supported by the lack of ISR signature in the proteomics data. Other than that, I have no further suggestions or comments.

Referee #3:

The new additions, in general, are welcomed and improve the paper.

However, there are some concerns that remain. I will focus attention on Figure 4.
-In the first place, it seems that the Atg RNAi was induced during both development and adult. Was post-development RNAi tested?
If so, this should be shown.

-The major concern is with Figures 4B-4F. It seems the 'control' is a gfp transgene with auxin. This doesn't make sense. The control should be the Atg RNAi with and without auxin. At the very least, this data should be included. The strength of the inducible system is to compare the effects of induced expression. Hence, it is critical to show the Atg RNAi with and without auxin induction.

That said, the data in Fig4L and 4M appears robust and very interesting.

Referee #1: The revised manuscript addresses my previous comments and is substantially improved. There are some very interesting additional observations and I am fully satisfied with the revised version. Regarding the accumulation of ATF4 in some local populations of Ref(2)P/P62 aggregates, this is very interesting and may suggest that specific types/populations of neurons are sensitised to accumulate ATF4. Investigating this is beyond the scope of the current work but is something that could be addressed in future studies by using single cell/single nuclear RNA-sequencing of brain or head tissue in this model.

We sincerely thank the reviewer for the positive feedback and fully agree that a deeper understanding of the interplay between Ref(2)P/P62 aggregates and ATF4 in different brain areas represents an interesting avenue for future investigations in our experimental model.

Referee #2: As per my comments in previous iterations, and not to repeat too much, the Sigrist lab have further explored their observation that impairment of autophagy in mushroom bodies generates a wider modification of active zone accumulation across the brain, improving sleep parameters and lifespan. This is explored at the molecular level with proteomic and transcriptomic changes characterised. The observation that degradative components from the MBs spread across the brain is interesting and although not mechanistically linked as yet to the active zone component increase, further describes aspects of the MB autophagy block as it affects the whole brain. The inclusion of autophagy block on other identifiable structure (particularly related to sleep) with induction of the brain-wide effect further ratifies the MBs as critical in this novel mechanism in neuronal homeostasis and aging. They have undertaken a number of novel experiments at the suggestion of the reviewers that have sharpened and refined the data. If anything, the dataset is now rather extensive, the authors have gone the extra yards. The paper is to my mind robust, of strong interest and high scientific merit. I have one v. minor comment, (which is optional) when discussing the lack of ISR response, to also add that the directed experiments are also supported by the lack of ISR signature in the proteomics data. Other than that, I have no further suggestions or comments.

We sincerely thank the reviewer for the positive feedback and for the suggested discussion point concerning the lack of ISR signatures in the proteomics data. We added this information in the discussion in lines 541-542.

Referee #3: The new additions, in general, are welcomed and improve the paper. However, there are some concerns that remain. I will focus attention on Figure 4. -In the first place, it seems that the Atg RNAi was induced during both development and adult. Was post-development RNAi tested? If so, this should be shown. -The major concern is with Figures 4B-4F. It seems the 'control' is a gfp transgene with auxin. This doesn't make sense. The control should be the Atg RNAi with and without auxin. At the very least, this data should be included. The strength of the inducible system is to compare the effects of induced expression. Hence, it is critical to show the Atg RNAi with and without auxin induction. That said, the data in Fig4L and 4M appears robust and very interesting.

We sincerely thank the reviewer for the positive feedback and for emphasizing the importance of appropriate controls especially for the inducible AGES experiments shown in Fig. 4A-F. We fully agree that, in principle, a direct comparison of *vt30559*-Gal4,AGES>UAS-*Atg5*-RNAi flies with and without Auxin supplementation would represent the most ideal experimental design to assess the specific effects of induced *Atg5*-RNAi expression in our experimental context.

To address the mentioned point, we first tested whether a temporally restricted, post-developmental induction of *Atg5*-RNAi would be sufficient to induce detectable autophagy impairment in the Mushroom Body (MB) Kenyon cells (KCs). Specifically, *vt30559*-Gal4,AGES>UAS-*Atg5*-RNAi flies were supplemented with 10 mM Auxin between day 1 and day 15 post-eclosion. Under these conditions however, we did not detect apparent Ref(2)P/P62 aggregate formation in the brain, including in MB KCs (see new Appendix Fig. S3). As 10 mM Auxin has been shown to be an effective and relatively high dose for the adult AGES system (PMID: 35363137), this could potentially be explained by a slow turnover rate of the ATG5 protein, consequently limiting the efficacy of the *Atg5*-RNAi to sufficiently reduce gene expression and to induce autophagy failure in the MB. We have now included these data in the revised manuscript as Appendix Fig. S3, and clarified this point in the Results section (Lines 233-238). As no detectable autophagy-deficient phenotype was observed, we did not further pursue lifespan analyses under these conditions.

We therefore aimed to increase the efficacy of the *Atg5*-RNAi by initiating Auxin supplementation already during development. However, previous studies have reported that Auxin exposure during larval development can delay developmental time in *Drosophila* (PMID: 35363137; PMID: 38240746), a phenomenon that we also observed in our experimental context (see Reviewer Figure 1). Consequently, a direct comparison of *vt30559*-Gal4,AGES>UAS-*Atg5*-RNAi flies raised with or without Auxin would compare animals with different developmental histories and physiological baselines, thereby confounding lifespan analyses. We have now mentioned this consideration in the Results section lines 239-241.

To overcome this limitation, we therefore focused our analysis on cohorts raised under identical Auxin conditions throughout development and until day 15 post-eclosion (Fig. 4A). For this purpose, we compared *vt30559*-Gal4,AGES>UAS-*Atg5*-RNAi flies to *vt30559*-Gal4,AGES>UAS-*mcd8*-GFP controls, as both UAS transgenes (BDSC #34899 and #32184) are inserted at the same attP2 genomic landing site and therefore have the same transgenic background. We would also like to emphasize that both lines were additionally backcrossed for 6 generations to minimize genetic background effects. We have now elaborated our experimental design in more detail in the Results part lines 242-245.

While we agree that this experimental design does not permit a strict within-genotype ±Auxin comparison of *vt30559*-Gal4,AGES>UAS-*Atg5*-RNAi flies, we are convinced that these experiments nevertheless provide important independent confirmation of both the PreScale and longevity phenotypes described in Fig. 1 and Fig. 3, respectively. In addition, they provide a valuable gain in temporal control by refining the time window of *Atg5*-RNAi induction to development and early adulthood.

Importantly, the observed phenotypes are consistent with the independent constitutive MB autophagy inhibition experiments (Fig. 3E–H) and are further supported by the temporally restricted survival paradigm shown in Fig. 4L–M. Together, we hope these findings adequately address the reviewer's concern and support our interpretation that early PreScale activation until day 15 post-eclosion is sufficient to promote survival.

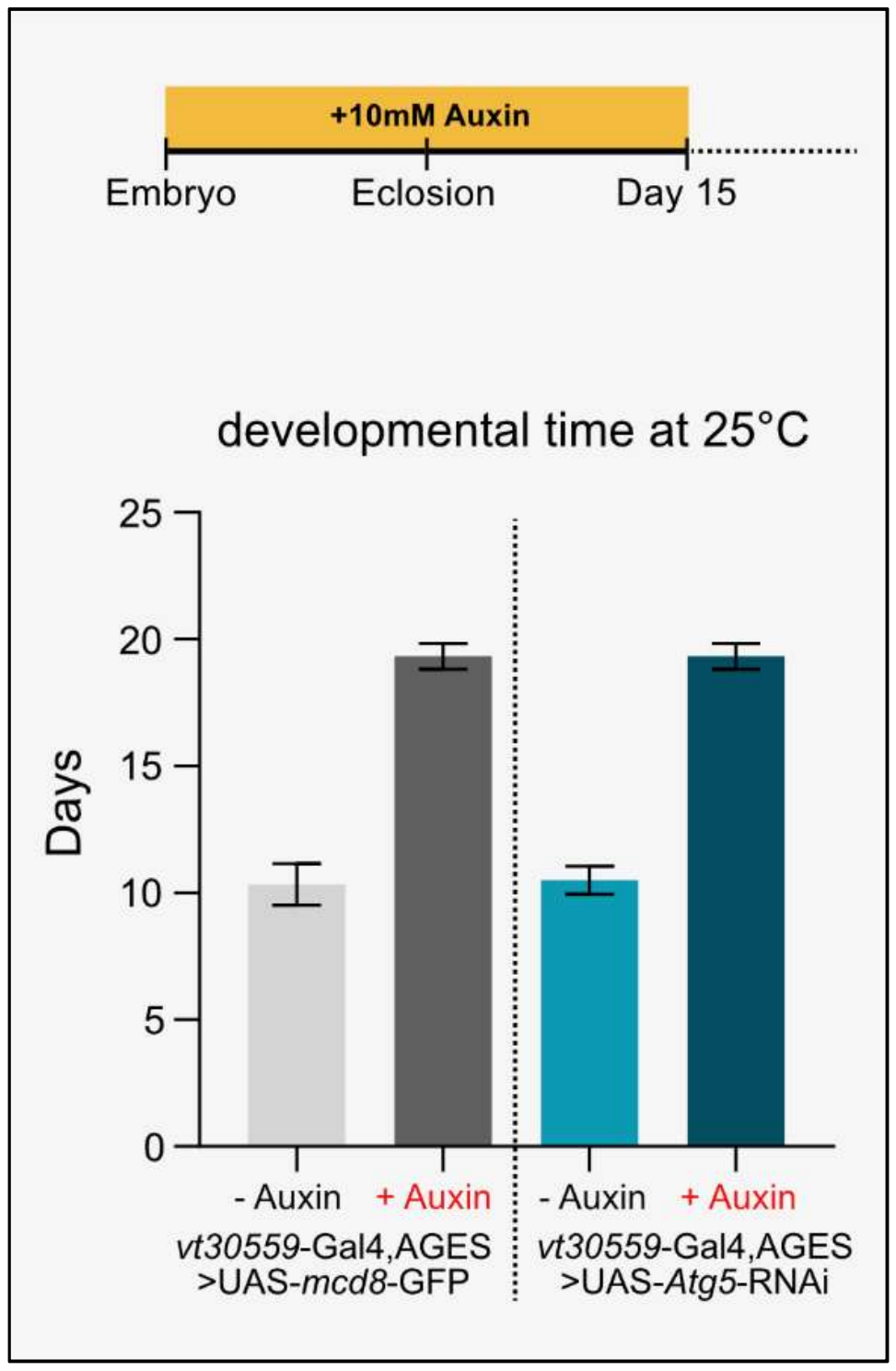

Reviewer Figure 1: Larval Auxin supplementation delays developmental time to eclosion in both experimental genotypes.

2nd Revision - Editorial Decision 28th May 2026

Dear Stephan,

Thank you for submitting your revised manuscript for our consideration. I am very pleased to inform you that referee #3 is satisfied with your responses to the remaining concerns from the last review round (the referee's comments are appended below).

In light of this input, I am happy to inform you that your manuscript has now been accepted for publication in The EMBO Journal. Congratulations on an excellent manuscript and thanks for comprehensively addressing the initially raised referees' concerns and our editorial requests for changes and corrections.

Your manuscript will be processed for publication by EMBO Press. It will be copy edited and you will receive page proofs prior to publication. Please note that you will be contacted by Springer Nature Author Services to complete licensing and payment information.

You may qualify for financial assistance for your publication charges - either via a Springer Nature fully open access agreement or an EMBO initiative. Check your eligibility: https://link.springer.com/journal/44318/how-to-publish-with-us

Should you be planning a Press Release on your article, please notify the production team via this form in order to coordinate publication and release dates: https://forms.cloud.microsoft/e/eH4A8JVP6g

If you have any questions, please do not hesitate to contact the Editorial Office. Thank you for your contribution to The EMBO Journal. Working with you has been a pleasure.

Best regards,

Ioannis

Ioannis Papaioannou, PhD
Editor, The EMBO Journal
i.papaioannou@embojournal.org

Referee #3:

I fully understand that there can be limitations to certain technical approaches. The authors have done a good job of explaining the rationale for their experimental data.

*****

Please note that it is The EMBO Journal policy for the transcript of the editorial process (containing referee reports and your response letters) to be published as an online supplement to each paper. If you should prefer removal of any referee-only figures included in the point-by-point response(s), e.g. because they may still be used for future publication or because they have been reproduced from published work by others, please do let us know immediately via response email.
More information is available here: https://link.springer.com/partners/embo-press/editorial-policies#Peer%20review

** Click here to be directed to your login page: https://emboj.msubmit.net

EMBO Press Author Checklist

Corresponding Author Name: Stephan J. Sigrist	
Journal Submitted to: The EMBO Journal	
Manuscript Number: EMBOJ-2025-122093	

USEFUL LINKS FOR COMPLETING THIS FORM
The EMBO Journal - Author Guidelines
EMBO Reports - Author Guidelines
Molecular Systems Biology - Author Guidelines
EMBO Molecular Medicine - Author Guidelines

Reporting Checklist for Life Science Articles (updated January
This checklist is adapted from Materials Design Analysis Reporting (MDAR) Checklist for Authors. MDAR establishes a minimum set of requirements in transparent reporting in the life sciences (see Statement of Task: 10.31222/osf.io/9sm4x). Please follow the journal's guidelines in preparing your
Please note that a copy of this checklist will be published alongside your article.

Abridged guidelines for figures
1. Data
The data shown in figures should satisfy the following conditions:

- ➡ the data were obtained and processed according to the field's best practice and are presented to reflect the results of the experiments in an accurate and unbiased manner.
- ➡ ideally, figure panels should include only measurements that are directly comparable to each other and obtained with the same assay.
- ➡ plots include clearly labeled error bars for independent experiments and sample sizes. Unless justified, error bars should not be shown for technical
- ➡ if n<5, the individual data points from each experiment should be plotted. Any statistical test employed should be justified.
- ➡ Source Data should be included to report the data underlying figures according to the guidelines set out in the authorship guidelines on Data

2. Captions
Each figure caption should contain the following information, for each panel where they are relevant:

- ➡ a specification of the experimental system investigated (eg cell line, species name).
- ➡ the assay(s) and method(s) used to carry out the reported observations and measurements.
- ➡ an explicit mention of the biological and chemical entity(ies) that are being measured.
- ➡ an explicit mention of the biological and chemical entity(ies) that are altered/varied/perturbed in a controlled manner.
- ➡ the exact sample size (n) for each experimental group/condition, given as a number, not a range;
- ➡ a description of the sample collection allowing the reader to understand whether the samples represent technical or biological replicates (including how many animals, litters, cultures, etc.).
- ➡ a statement of how many times the experiment shown was independently replicated in the laboratory.
- ➡ definitions of statistical methods and measures:
  - common tests, such as t-test (please specify whether paired vs. unpaired), simple χ2 tests, Wilcoxon and Mann-Whitney tests, can be unambiguously identified by name only, but more complex techniques should be described in the methods section;
  - are tests one-sided or two-sided?
  - are there adjustments for multiple comparisons?
  - exact statistical test results, e.g., P values = x but not P values < x;
  - definition of 'center values' as median or average;
  - definition of error bars as s.d. or s.e.m.

Please complete ALL of the questions below.
Select "Not Applicable" only when the requested information is not relevant for your study.

Materials

Newly Created Materials	Information included in the manuscript?	In which section is the information available? (Reagents and Tools Table, Materials and Methods, Figures, Data Availability Section)
New materials and reagents need to be available; do any restrictions apply?	Yes	Reagents and Tools Tabe; Data availability Section

Antibodies	Information included in the manuscript?	In which section is the information available? (Reagents and Tools Table, Materials and Methods, Figures, Data Availability Section)
For **antibodies** provide the following information:<br>- Commercial antibodies: RRID (if possible) or supplier name, catalogue number and or/clone number<br>- Non-commercial: RRID or citation	Yes	Reagents and Tools Table

DNA and RNA sequences	Information included in the manuscript?	In which section is the information available? (Reagents and Tools Table, Materials and Methods, Figures, Data Availability Section)
**Short novel DNA or RNA including primers, probes**: provide the sequences.	Yes	Material and Methods

Cell materials	Information included in the manuscript?	In which section is the information available? (Reagents and Tools Table, Materials and Methods, Figures, Data Availability Section)
**Cell lines:** Provide species information, strain. Provide accession number in repository **OR** supplier name, catalog number, clone number, and/**OR** RRID.	Not Applicable	
**Primary cultures:** Provide species, strain, sex of origin, genetic modification status.	Not Applicable	
Report if the cell lines were recently **authenticated** (e.g., by STR profiling) and tested for mycoplasma contamination.	Not Applicable	

Experimental animals	Information included in the manuscript?	In which section is the information available? (Reagents and Tools Table, Materials and Methods, Figures, Data Availability Section)
**Laboratory animals or Model organisms:** Provide species, strain, sex, age, genetic modification status. Provide accession number in repository **OR** supplier name, catalog number, clone number, **OR** RRID.	Yes	Reagents and Tools Table
**Animal observed in or captured from the field:** Provide species, sex, and age where possible.	Not Applicable	
Please detail **housing and husbandry conditions**.	Yes	Material and Methods

Plants and microbes	Information included in the manuscript?	In which section is the information available? (Reagents and Tools Table, Materials and Methods, Figures, Data Availability Section)
**Plants:** provide species and strain, ecotype and cultivar where relevant, unique accession number if available, and source (including location for collected wild specimens).	Not Applicable	
**Microbes:** provide species and strain, unique accession number if available, and source.	Not Applicable	

Human research participants	Information included in the manuscript?	In which section is the information available? (Reagents and Tools Table, Materials and Methods, Figures, Data Availability Section)
If collected and within the bounds of privacy constraints report on age, sex and gender or ethnicity for all study participants.	Not Applicable	

Core facilities	Information included in the manuscript?	In which section is the information available? (Reagents and Tools Table, Materials and Methods, Figures, Data Availability Section)
If your work benefited from core facilities, was their service mentioned in the acknowledgments section?	Yes	Acknowledgements and Material and Methods

Design

Study protocol	Information included in the manuscript?	In which section is the information available? (Reagents and Tools Table, Materials and Methods, Figures, Data Availability Section)
If study protocol has been **pre-registered, provide DOI in the manuscript**. For clinical trials, provide the trial registration number **OR** cite DOI.	Not Applicable	
Report the **clinical trial registration number** (at ClinicalTrials.gov or equivalent), where applicable.	Not Applicable	

Laboratory protocol	Information included in the manuscript?	In which section is the information available? (Reagents and Tools Table, Materials and Methods, Figures, Data Availability Section)
Provide DOI OR other citation details if **external detailed step-by-step protocols** are available.	Not Applicable	

Experimental study design and statistics	Information included in the manuscript?	In which section is the information available? (Reagents and Tools Table, Materials and Methods, Figures, Data Availability Section)
Include a statement about **sample size** estimate even if no statistical methods were used.	Not Applicable	
Were any steps taken to minimize the effects of subjective bias when allocating animals/samples to treatment (e.g. **randomization procedure**)? If yes, have they been described?	Not Applicable	
Include a statement about **blinding** even if no blinding was done.	Not Applicable	
Describe **inclusion/exclusion criteria** if samples or animals were excluded from the analysis. Were the criteria pre-established?<br><br>If sample or data points were omitted from analysis, report if this was due to attrition or intentional exclusion and provide justification.	Not Applicable	
For every figure, are **statistical tests** justified as appropriate? Do the data meet the assumptions of the tests (e.g., normal distribution)? Describe any methods used to assess it. Is there an estimate of variation within each group of data? Is the variance similar between the groups that are being statistically compared?	Yes	Figure Legends and Material and Methods

Sample definition and in-laboratory replication	Information included in the manuscript?	In which section is the information available? (Reagents and Tools Table, Materials and Methods, Figures, Data Availability Section)
In the figure legends: state number of times the experiment was **replicated** in laboratory.	Yes	Figure Legends
In the figure legends: define whether data describe **technical or biological replicates**.	Yes	Figure Legends

Ethics

Ethics	Information included in the manuscript?	In which section is the information available? (Reagents and Tools Table, Materials and Methods, Figures, Data Availability Section)
Studies involving **human participants**: State details of **authority granting ethics approval** (IRB or equivalent committee(s), provide reference number for approval.	Not Applicable	
Studies involving **human participants**: Include a statement confirming that **informed consent** was obtained from all subjects and that the experiments conformed to the principles set out in the WMA Declaration of Helsinki and the Department of Health and Human Services Belmont Report.	Not Applicable	
Studies involving **human participants:** For publication of **patient photos**, include a statement confirming that consent to publish was obtained.	Not Applicable	
Studies involving experimental **animals**: State details of **authority granting ethics approval** (IRB or equivalent committee(s), provide reference number for approval. Include a statement of compliance with ethical regulations.	Not Applicable	
Studies involving **specimen and field samples:** State if relevant **permits** obtained, provide details of authority approving study; if none were required, explain why.	Not Applicable	

Dual Use Research of Concern (DURC)	Information included in the manuscript?	In which section is the information available? (Reagents and Tools Table, Materials and Methods, Figures, Data Availability Section)
Could your study fall under dual use research restrictions? Please check biosecurity documents and list of **select agents and toxins** (CDC): https://www.selectagents.gov/sat/list.htm	Not Applicable	
If you used a select agent, is the security level of the lab appropriate and reported in the manuscript?	Not Applicable	
If a study is subject to dual use research of concern regulations, is the name of the **authority granting approval and reference number** for the regulatory approval provided in the manuscript?	Not Applicable	

Reporting

The MDAR framework recommends adoption of discipline-specific guidelines, established and endorsed through community initiatives. Journals have their own policy about requiring specific guidelines and recommendations to complement MDAR.

Adherence to community standards	Information included in the manuscript?	In which section is the information available? (Reagents and Tools Table, Materials and Methods, Figures, Data Availability Section)
State if relevant guidelines or checklists (e.g., **ICMJE, MIBBI, ARRIVE, PRISMA**) have been followed or provided.	Not Applicable	
For **tumor marker prognostic studies,** we recommend that you follow the **REMARK** reporting guidelines (see link list at top right). See author guidelines, under 'Reporting Guidelines'. Please confirm you have followed these guidelines.	Not Applicable	
For **phase II and III randomized controlled trials**, please refer to the **CONSORT** flow diagram (see link list at top right) and submit the CONSORT checklist (see link list at top right) with your submission. See author guidelines, under 'Reporting Guidelines'. Please confirm you have submitted this list.	Not Applicable	

Data Availability

Data availability	Information included in the manuscript?	In which section is the information available? (Reagents and Tools Table, Materials and Methods, Figures, Data Availability Section)
Have **primary datasets** been deposited according to the journal's guidelines (see 'Data Deposition' section) and the respective accession numbers provided in the Data Availability Section?	Yes	Data Availability Section
Were **human clinical and genomic datasets** deposited in a public access-controlled repository in accordance to ethical obligations to the patients and to the applicable consent agreement?	Not Applicable	
Are **computational models** that are central and integral to a study available without restrictions in a machine-readable form? Were the relevant accession numbers or links provided?	Not Applicable	
If publicly available data were reused, provide the respective **data citations in the reference list**.	Not Applicable	
